# Exploring the Electrochemistry of Iron Dithiolene and Its Potential for Electrochemical Homogeneous Carbon Dioxide Reduction

**DOI:** 10.1002/celc.202200610

**Published:** 2022-08-04

**Authors:** Craig G. Armstrong, Mark Potter, Thomas Malcomson, Ross W. Hogue, Sapphire M. Armstrong, Andrew Kerridge, Kathryn E. Toghill

**Affiliations:** ^1^ Department of Chemistry Lancaster University Lancaster LA1 4YB United Kingdom; ^2^ Department of Chemistry School of Natural Sciences The University of Manchester Manchester M13 9PL United Kingdom; ^3^ Leiden Institute of Chemistry LIC/Energy & Sustainability Gorlaeus Laboratories Einsteinweg 55 2333 CC Leiden

**Keywords:** CO_2_ reduction, density functional theory, homogeneous catalysis, iron dithiolene, mechanistic insights

## Abstract

In this work, the dithiolene complex iron(III) bis‐maleonitriledithiolene [Fe(mnt)_2_] is characterised and evaluated as a homogeneous CO_2_ reduction catalyst. Electrochemically the Fe(mnt)_2_ is reduced twice to the trianionic Fe(mnt)_2_^3−^ state, which is correspondingly found to be active towards CO_2_. Interestingly, the first reduction event appears to comprise overlapping reversible couples, attributed to the presence of both a dimeric and monomeric form of the dithiolene complex. In acetonitrile Fe(mnt)_2_ demonstrates a catalytic response to CO_2_ yielding typical two‐electron reduction products: H_2_, CO and CHOOH. The product distribution and yield were governed by the proton source. Operating with H_2_O as the proton source gave only H_2_ and CO as products, whereas using 2,2,2‐trifluoroethanol gave 38 % CHOOH faradaic efficiency with H_2_ and CO as minor products.

## Graphical abstract

**The first iron‐based dithiolene complex**, iron(III) bis‐maleonitriledithiolene, is characterised for carbon dioxide reduction activity and stability. Operating with 2,2,2‐trifluoroethanol proton source in acetonitrile electrolyte, the homogeneous catalyst gave 38 % CHOOH faradaic efficiency with H_2_ and CO minor products. The catalyst performance is presently limited by parasitic side‐reactions with the electrolyte and catalyst decomposition.

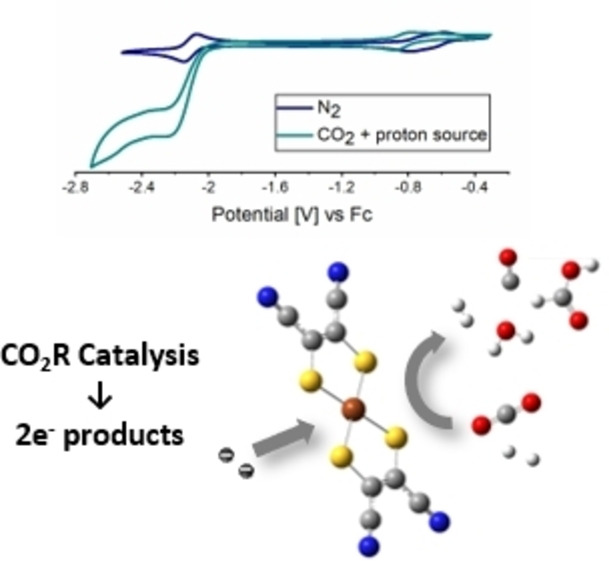

## Introduction

Many molecular catalysts have been developed over the past few decades targeting electrochemical CO_2_ reduction (CO_2_R), with CO and CHOOH being the most common products formed.^[1]^ Due to the unique coordination environments of metal coordination compounds (MCCs), molecular catalysts generally display enhanced product selectivity because their properties are inherently tuneable by modification of the ligand structures and the reaction conditions.^[2]^ The catalyst activity (reaction rates and overpotentials) and selectivity (including hydrogen evolution reaction (HER) suppression) can be synthetically controlled by modifying the reactant‐adduct coordination site.

For CO_2_R the highest performing molecular catalysts thus far have featured undesirably expensive noble metals such as rhenium,[^3^, ^4^] rhodium^[5]^ and ruthenium.[^6^, ^7^] Recent efforts have pursued more earth‐abundant elements in the interest of cost and sustainability, such as those comprised of nickel,[^8^, ^9^] manganese,^[10]^ cobalt[^8^, ^9^] and most notably iron.[^11^, ^12^, ^13^, ^14^] Complexes such as the bio‐mimetic Fe‐porphyrin derivatives, first pioneered by Savéant and co‐workers,^[13]^ display promising catalytic activity and may in principal be derived from biological feedstocks making their application appealing from an industrial perspective. Further noteworthy examples of Fe‐based catalysts include Fe carbonyl clusters,^[14]^ phenanthrolines^[15]^ and cyclopentadienones.^[11]^ Yet surprisingly few Fe‐based molecular catalysts have been explored compared to those utilising platinum group metal centres.

In the search of more diverse Fe‐based CO_2_R molecular catalysts, we identified that dithiolene‐based complexes have been underexplored with no Fe‐dithiolene derivatives reported so far.^[16]^ Bidentate dithiolene ligands (composed of two thiolate donors) display redox non‐innocence whereby the ligands are complicit in redox activity about the metal centre.^[17]^ Metal dithiolene complexes are known to be catalytically active towards HER,[^18^, ^19^, ^20^, ^21^, ^22^, ^23^, ^24^, ^25^] making their application in CO_2_R catalysis interesting, given that the HER could be selectively suppressed. The redox character of these complexes arises from the central metal‐sulfur core (MS_4_ or MS_6_) which is analogous and bio‐mimetic of the iron‐sulfur clusters found in many enzymes.[^26^, ^27^] Such reaction centres are known to conduct a wide range of redox reactions, including CO‐dehydrogenases which catalyse the conversion of CO_2_ to CO.^[28]^

In 2018 Fogeron et al. first reported on the application of dithiolene complexes in CO_2_R using bio‐mimetic molybdenum pyranopterin‐dithiolene^[29]^ and nickel molybdopterin‐dithiolene[^30^, ^31^] as homogeneous catalysts. These catalysts achieved relatively high faradaic efficiencies for CHOOH production of 60 % and 39 % respectively, with CO and H_2_ as minor products. The catalytic activity of these square‐planar complexes was attributed to the MS_4_ coordination environment which is simultaneously electron‐rich and valence‐deficient, thus allowing facile formation of a CO_2_‐adduct at the metal centre and subsequent reaction. While the complexes above showed promising CO_2_R activity, their syntheses and experimental conditions were complicated with mercury used as an electrode material,^[31]^ presumably to further supress parasitic HER.

**Figure 1:**
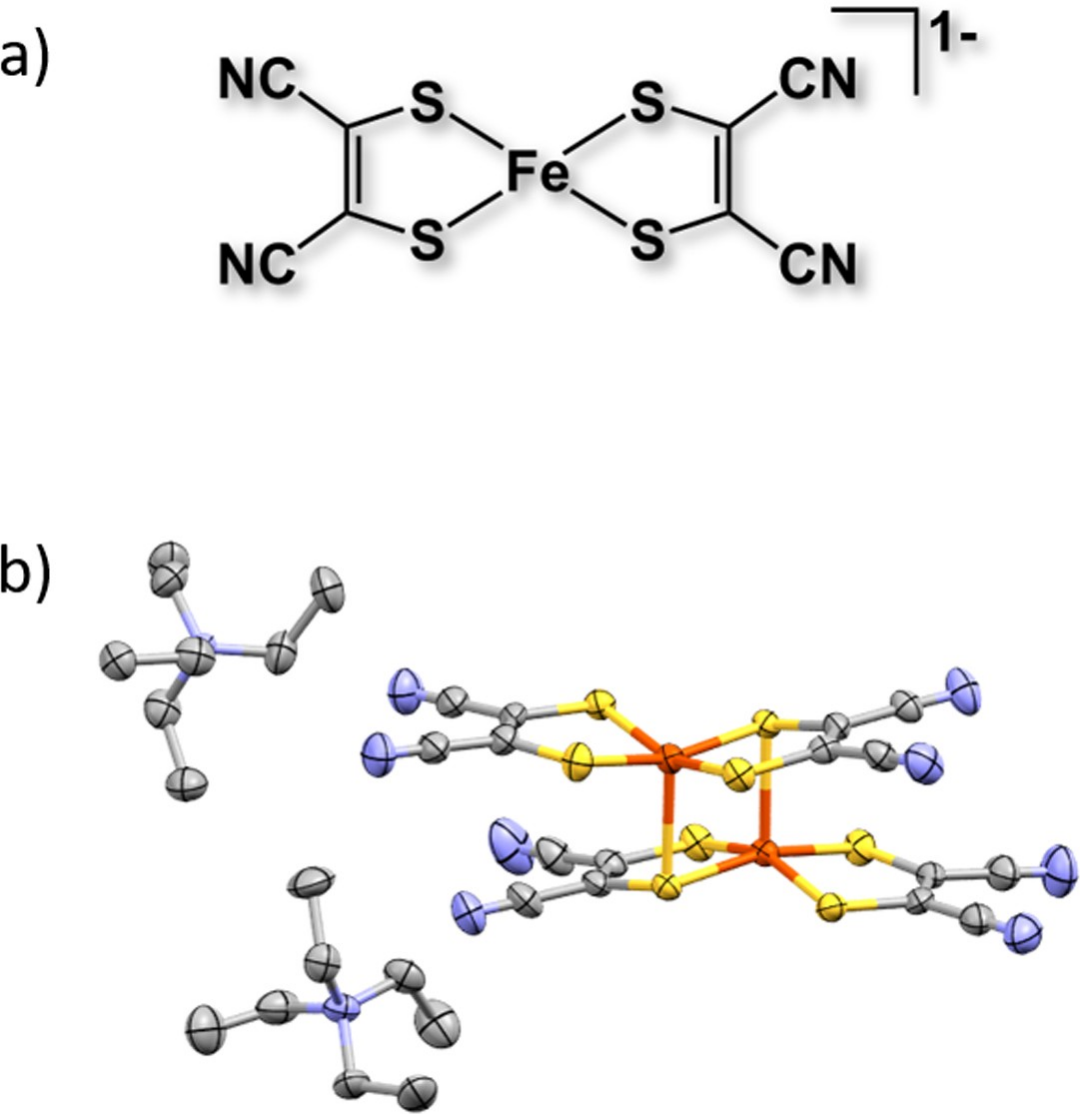
a) Structure of the iron(III) bis‐maleonitriledithiolene complex Fe(mnt)_2_^1−^. b) Crystal structure of (TEA)_2_[Fe_2_(mnt)_4_] obtained from single crystal XRD analysis; ellipsoids set at 50 % probability. Hydrogen atoms have been omitted for clarity. CIF files can be found in the SI.

In the present work we evaluated the response of a range of simpler metal bis‐maleonitriledithiolene complexes for HER and CO_2_R activity in non‐aqueous conditions. Of the complexes studied the Fe(mnt)_2_^1−^ which possesses an analogous square‐planar FeS_4_ coordination (Figure 1a) was highly responsive to the presence of CO_2_ and underwent further characterisation and evaluation as a CO_2_R molecular catalyst. In addition, a mechanistic study by use of density functional theory (DFT) was conducted to rationalise the selectivity of Fe(mnt)_2_.

## Results and Discussion

### Synthesis and Characterisation

The preparation of the iron(III) bis‐maleonitriledithiolene catalyst proceeds via a simple three step method starting from low toxicity and inexpensive starting materials. Initially, the ligand was prepared by reaction of chloroacetonitrile with sulfur and then subsequent dimerisation of the cyanodithioformate intermediate. The complexation of iron(III) chloride with excess ligand at room temperature yields (TEA)[Fe(mnt)_2_] as a crystalline powder (TEA=tetraethylammonium cation). In the present work, yields of 31, 21 and 66 % were obtained for the cyanodithioformate intermediate, maleonitriledithiolate ligand and (TEA)[Fe(mnt)_2_] complex, respectively. In comparison, higher yields have been demonstrated in the literature giving 71, 97 and 54 %,[^32^, ^33^, ^34^] respectively. The catalyst was isolated as the dihydrate (TEA)[Fe(mnt)_2_] ⋅ 2H_2_O, as determined by elemental analysis (Supporting Information, SI). The presence of two stoichiometric quantities of water is inconsequential in the current study as proton source concentrations of at least 0.1 M 2,2,2‐trifluoroethanol (TFE) or 1 M H_2_O were used which far exceeds the 0.002 M water originating from the catalyst material.

Crystals suitable for powder and single‐crystal X‐ray diffraction (XRD) analysis were grown by slow recrystallisation from EtOH to give black anhydrous needles (SI). Single‐crystal XRD analysis shows that the Fe(mnt)_2_ catalyst exists as the binuclear dimer [Fe_2_(mnt)_4_]^2−^ in the solid phase whereby each Fe centre adopts a square pyramidal coordination with five sulfur donors, giving a biologically relevant Fe_2_S_8_ core, shown in Figure 1b. This was consistent with previous works[^35^, ^36^] and in addition, our experimentally obtained powder XRD pattern matched well with simulated data from the single‐crystal analysis (Figure S1).

### Voltammetric Characterisation

#### Metal dithiolene complex response to water and CO_2_

**Figure 2:**
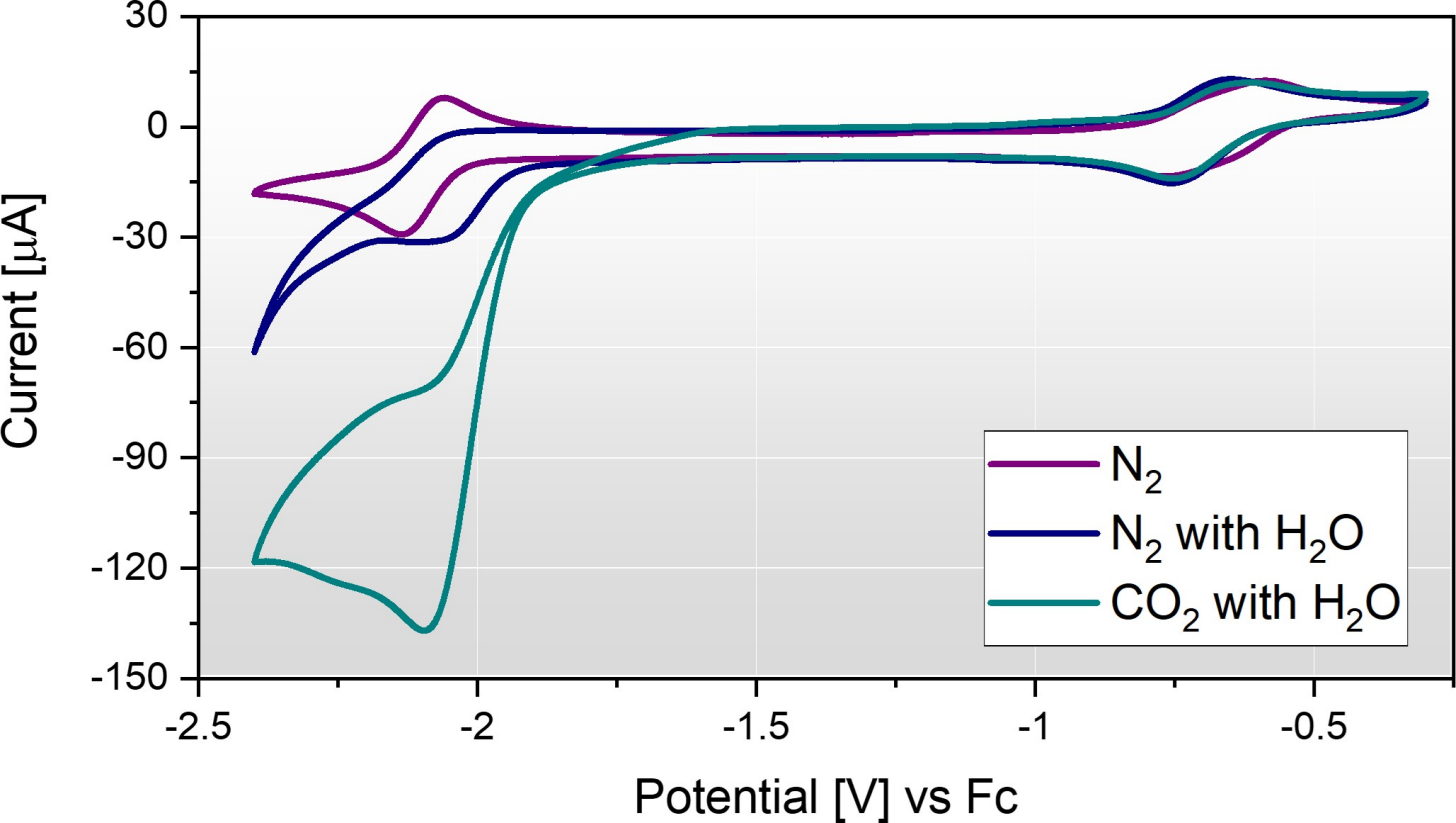
Cyclic voltammetry of Fe(mnt)_2_^1−^ at a glassy‐carbon (GC) electrode under N_2_ in the absence of a proton source, and under both N_2_ and CO_2_ in the presence of 3 M water (100 mV s^−1^, second scans shown). The electrolyte was composed of 1 mM (TEA)[Fe(mnt)_2_] with 100 mM tetrabutylammonium hexafluorophosphate (TBAPF_6_) supporting electrolyte in acetonitrile (MeCN) solvent.

Building on our previous work employing metal dithiolenes in redox flow batteries,^[37]^ we screened the complexes Fe(mnt)_2_^1−^, Co(mnt)_2_^2−^, Ni(mnt)_2_^2−^ and Cu(mnt)_2_^2−^ as molecular catalysts for HER and CO_2_R due to accessible redox states at suitably negative potentials (Figure S5). The Ni and Cu complexes showed no response to the presence of CO_2_, with the voltammetric trace overlaying that of N_2_ and a proton source. Co(mnt)_2_^2−^ showed a slight increase in cathodic current when in the presence of CO_2_, but the response was considered catalytically negligible (Figure S5a). The onset of HER is also not tied to the reduction of the complex, indicating no catalytic activity. The Fe complex stood out as possessing a high catalytic response towards CO_2_R as shown in Figure 2. Thus, we targeted Fe(mnt)_2_^1−^ for full characterisation. Herein the electrochemical properties of Fe(mnt)_2_ are discussed as a redox material in aprotic electrolyte and as a catalyst for HER and CO_2_R in protic media.

#### Voltammetric characterisation of Fe(mnt)_2_^1−^

Although the Fe(mnt)_2_^1−^ complex was first prepared and studied over 50 years ago, its electrochemistry has been essentially unreported. Indeed, the complex was only briefly characterised in the work of Yamaguchi *et al*. in 2009 and by us in 2019.^[37]^ Therefore, a more thorough voltammetric investigation was conducted here to evaluate its properties in MeCN electrolyte.

**Figure 3:**
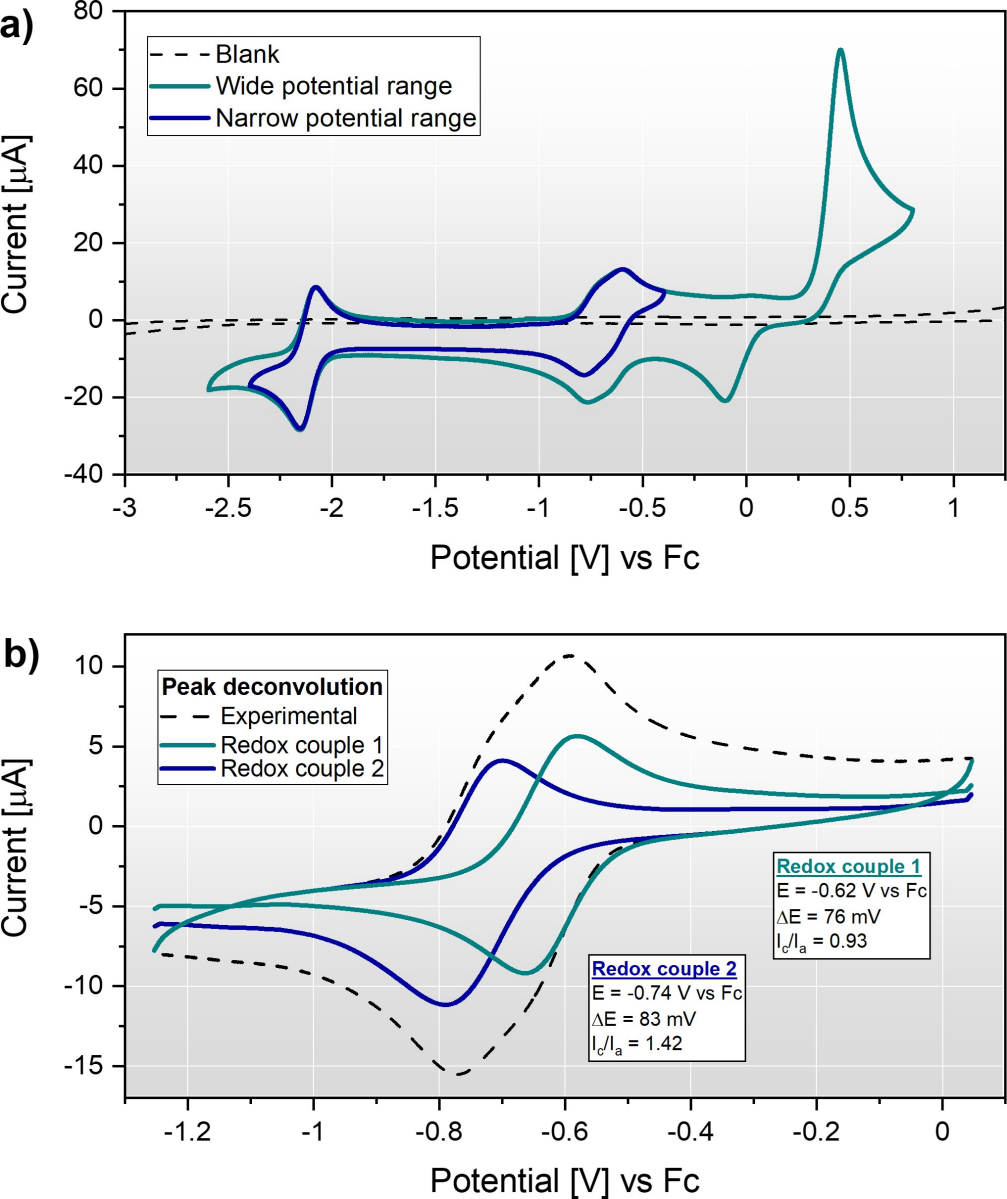
Cyclic voltammetry of Fe(mnt)_2_^1−^ at a GC electrode under N_2_ and in the absence of a proton source (100 mV s^−1^, second scans shown). a) Behaviour of Fe(mnt)_2_^1−^ over a range of applied potentials. b) Peak deconvolution of the Fe(mnt)_2_^1−/2−^ redox couple into two diffusion‐limited redox couples. The electrolyte was composed of 1 mM (TEA)[Fe(mnt)_2_] with 100 mM TBAPF_6_ supporting electrolyte in MeCN solvent.

Under inert conditions and in the absence of a proton source, the as synthesised anionic Fe(mnt)_2_^1−^ species undergoes two sequential one‐electron reductions to the trianionic Fe(mnt)_2_^3−^ oxidation state at −0.69 and −2.11 V vs ferrocene (Fc) as shown in Figure 3a. At anodic potentials the complex is also redox active, giving a large oxidation peak at 0.46 V vs. Fc. This redox wave lacks a symmetrical back‐reduction peak in the expected potential range (0.25 to 0.40 V vs. Fc) and instead a broad reduction peak is observed at −0.1 V vs Fc. It is attributed to oxidative decomposition of the complex which most likely causes dissociation of the dithiolene ligands and formation of an unknown solvated Fe(III) species. Throughout the present study, the working electrode was operated at more negative potentials (<0 V vs. Fc) to avoid this irreversible decomposition of the catalyst. Furthermore, a membrane was employed to separate the catalyst solution from the counter electrode during electrolysis.

Peak analysis of the Fe(mnt)_2_^2−/3−^ redox couple gave a high peak current ratio close to unity (0.89) and a peak‐to‐peak separation of 77 mV (at a scan rate of 100 mV s^−1^) and a modest increase in peak separation at higher scan rates confirms quasi‐reversible kinetics. In contrast, the Fe(mnt)_2_^1−/2−^ couple shows more complicated voltammetry, illustrated in Figure 3b. Here the waveform is most consistent with the superposition of two different redox reactions close in potential. Peak deconvolution yields two quasi‐reversible redox couples at −0.62 and −0.74 V vs. Fc displaying comparable peak currents and peak separations of 76 and 83 mV, respectively (the reconstructed curve is provided in Figure S6 for comparison to the experimental data). This suggests the presence of two distinct chemical species in the electrolyte with similar electrochemical properties. Integration of the assumed Fe(mnt)_2_^1−/2−^ process gives a passed charge comparable to the Fe(mnt)_2_^2−/3−^ couple (Figure S7) suggesting that the redox events are stoichiometrically one‐electron processes.

To account for the observed two diffusion‐limited redox couples which are identified in the peak deconvolution we postulate that the dimer and monomer forms of the dithiolene complex are both present in solution. As shown in Figure 1b the Fe(mnt)_2_ catalyst exists as the binuclear dimer Fe_2_(mnt)_4_^2−^ in the solid phase due to more favourable square pyramidal coordination. Yet, in solution phase it was thought that the dimer is dissociated into monomer units^[17]^ based upon magnetic susceptibility and electron paramagnetic resonance spectroscopy experiments performed by Williams *et al*.^[38]^ This work measured the spin state (S) of the complex in solution as S=^3^/_2_ (quartet) and in the solid state as S=^1^/_2_ (doublet), however the cause of this disparity was not formally identified and may result from a change in electronic structure. Considering that the Fe−S bond length between Fe(mnt)_2_ units is only 0.2 Å longer than Fe−S bond lengths within each Fe(mnt)_2_ unit, the dimer binding is evidently strong. Therefore, we hypothesise that the Fe_2_(mnt)_4_^2−^ dimer formally exists in solution or dominates a fast‐exchanging equilibrium with the monomeric form:

$$\left[\mathrm{Fe}{}_{2}\left(\mathrm{mnt}\right){}_{4}\right]{}^{2-}{}_{\left(\mathrm{aq}\right)}\vec{\leftarrow }2\left[\mathrm{Fe}\left(\mathrm{mnt}\right){}_{2}\right]{}^{1-}{}_{\left(\mathrm{aq}\right)}$$

The co‐existence of the dimer and monomer species in solution of other Fe‐dithiolene complexes has been previously reported,[^39^, ^40^] though not reported voltammetrically to the best of our knowledge. The two species are assumed to exhibit slightly different redox potentials with the dimer more easily reduced, as observed in the voltammetry, yet possess *similar* diffusion coefficients and electrode kinetics (parameters could not obtained in the present work). The electronic stoichiometry is preserved because the integrated charge of the Fe(mnt)_2_^1−/2−^ and Fe(mnt)_2_^2−/3−^ redox couples are both dependent on the total quantity of Fe(mnt)_2_ units at the electrode interface, irrespective of monomeric or dimeric form. Based on the similar peak currents of the deconvoluted peaks, we propose that the redox processes are sequential, and each constitute half of the total Fe(mnt)_2_ units in the diffusion layer. As such, the reduction of Fe_2_(mnt)_4_^2−^ proceeds to the trianionic Fe_2_(mnt)_4_^3−^ dimer. This rapidly dissociates to give Fe(mnt)_2_^2−^ and the Fe(mnt)_2_^1−^ monomer, which is then further reduced at the lower redox potential. Here we hypothesise that the Fe_2_(mnt)_4_^3−^ dissociation is a dynamic equilibrium driven by unfavourable Fe−S bond lengthening between Fe(mnt)_2_ units due to addition of electron density in the Fe−S core upon reduction. Overall, the proposed reactions are:

Redox couple 1:

$${\left[{Fe}_{2}{\left(mnt\right)}_{4}\right]}^{2-}+e^{-}\vec{\leftarrow }{\left[{Fe}_{2}{\left(mnt\right)}_{4}\right]}^{3-}$$

Dimerisation/dissociation:

$${\left[{Fe}_{2}{\left(mnt\right)}_{4}\right]}^{3-}\vec{\leftarrow }{\left[Fe{\left(mnt\right)}_{2}\right]}^{2-}+{\left[Fe{\left(mnt\right)}_{2}\right]}^{1-}$$

Redox couple 2:

$${\left[Fe{\left(mnt\right)}_{2}\right]}^{1-}+e^{-}\vec{\leftarrow }{\left[Fe{\left(mnt\right)}_{2}\right]}^{2-}$$

Overall:

$${\left[{Fe}_{2}{\left(mnt\right)}_{4}\right]}^{2-}+2e^{-}\vec{\leftarrow }{2\;\left[Fe{\left(mnt\right)}_{2}\right]}^{2-}$$

**Figure 4:**
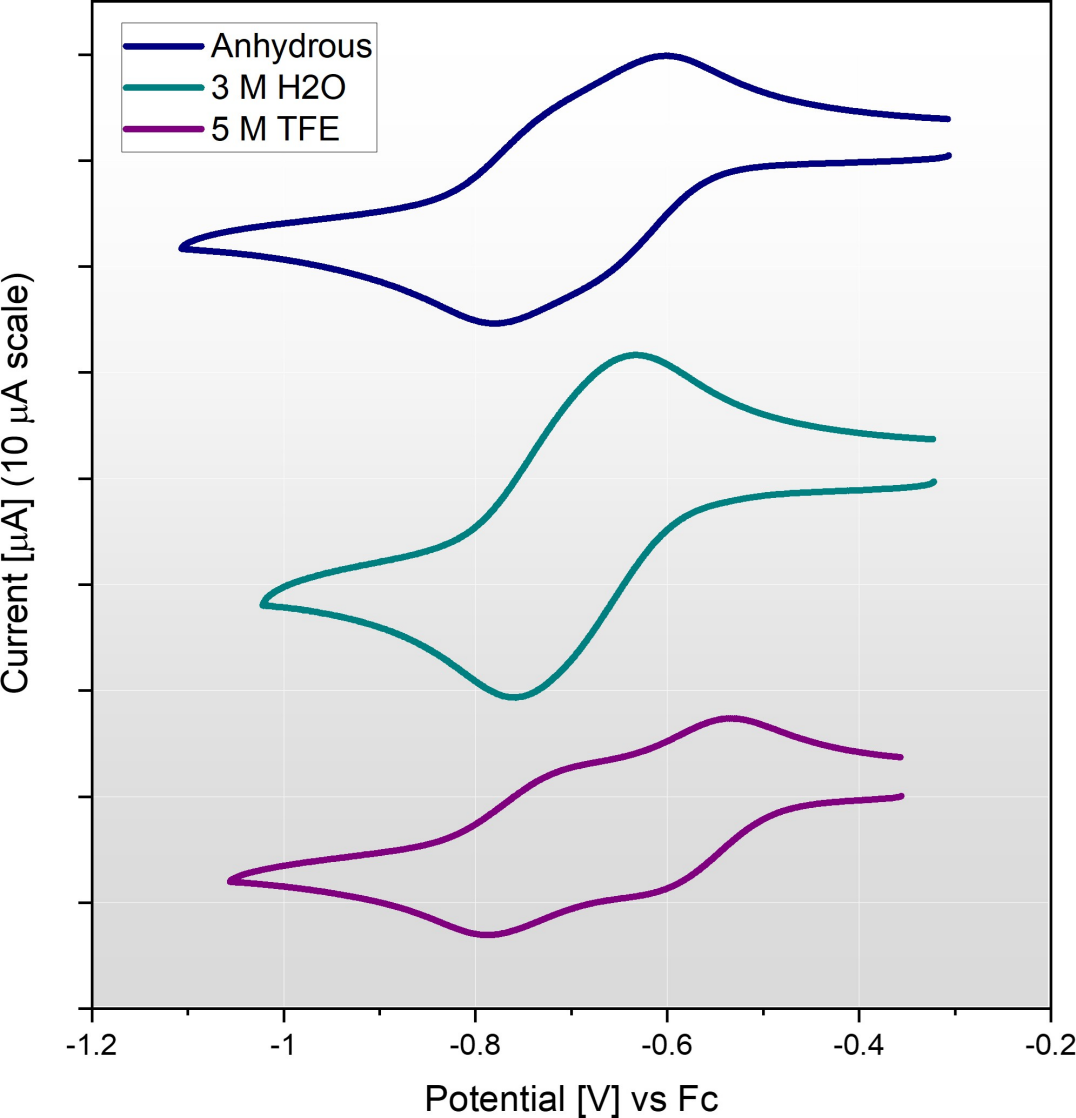
Cyclic voltammetry of the Fe(mnt)_2_^1−/2−^ redox couple at 100 mV s^−1^ at a GC electrode, under N_2_ (first scan shown). An anhydrous electrolyte is compared to one containing 3 M H_2_O and one containing 5 M TFE. The electrolyte was composed of 1 mM (TEA)[Fe(mnt)_2_] with 100 mM TBAPF_6_ supporting electrolyte in MeCN solvent.

Interestingly, when dissolved in a concentrated hydrous electrolyte (3 M H_2_O), we do not observe the two‐peak waveforms exhibited in Figure 3b. Instead, a single redox couple is found (comparison given in Figure 4, peak analysis given in Figure S11) with larger peak currents and comparatively smaller peak separation of 128 mV (at 100 mV s^−1^). To account for this behaviour, we hypothesise that the Fe(mnt)_2_^1−^ species coordinates with water to give a pseudo‐octahedral geometry which causes more favourable dissociation of the Fe_2_(mnt)_4_^2−^ dimer. Thus, the reduction of Fe(mnt)_2_^1−^ proceeds in a single step one‐electron process. In stark contrast, voltammetry performed in an electrolyte containing 5 M TFE caused further separation of the superimposed redox couples as shown in Figure 4. It is therefore evident that the electrochemistry of the Fe(mnt)_2_^1−/2−^ redox couple is heavily dependent on the solvation and electrolyte composition. To investigate this further, voltammetry using different supporting electrolytes was conducted in the absence of proton source and under N_2_ (Figure S14). The use of highly coordinating halide anions caused the Fe(mnt)_2_^1−/2−^ waveform to revert to a classical fully reversible redox couple exhibiting a small peak separation of 72 mV and accompanied negative shift of ∼100 mV. This result indicates a shift of the equilibrium towards full dissociation of the dimer due to coordination of halide ligands.

The electronic structure of the Fe(mnt)_2_ in these solvation environments was then probed using UV/vis spectroscopy. It was found that in all electrolytes examined the iron dithiolene solution showed high intensity bands in the UV region corresponding to spin‐allowed intraligand ($\pi -\pi^{{}^{*}})$ transitions (Figure S25 and Figure S26). A lower energy peak at 450 nm was also observed for the anhydrous MeCN and in the presence of 5 M TFE, the latter being more intense. However, in the presence of 3 M H_2_O or with highly coordinating halide ions present, the peak at 450 nm splits into two broader peaks either side of 450 nm with much lower intensity. Evidently, there is significant change in the electronic structure associated with dissociation of the dimer in solution.

#### Electrocatalytic activity

**Figure 5:**
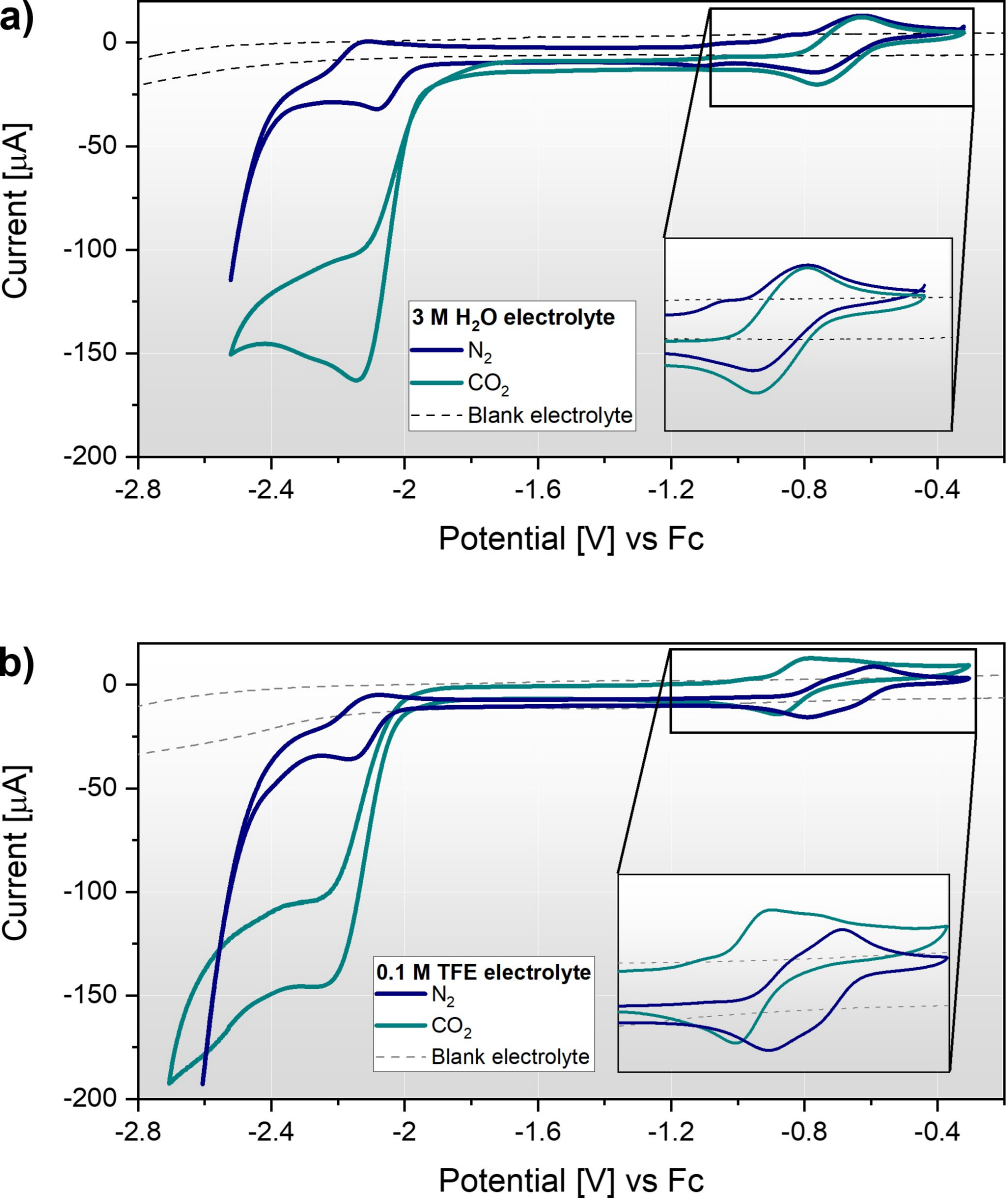
Cyclic voltammograms of 1 mM (TEA)[Fe(mnt)_2_] in 100 mM TBAPF_6_/MeCN electrolyte at a GC electrode, recorded at 100 mV s^−1^ (second scans shown). The catalytic activity towards HER and CO_2_R are compared for electrolytes containing a) 3 M H_2_O and b) 100 mM TFE proton sources. The blank electrolyte response under CO_2_ is shown by dashed black lines. In each graph a magnified view of the Fe(mnt)_2_^1−/2−^ redox couple is shown in the corresponding inset graphs.

In the presence of CO_2_ the Fe(mnt)_2_^3−^ catalyst displays a loss of reversibility due to a coupled homogeneous chemical reaction with CO_2_ as shown in Figure S13. This promising behaviour indicated likely catalytic activity, facilitated by the trace water in the electrolyte originating from the synthesised catalyst dihydrate (proton source diffusion limitation). However, upon addition of an excess of a suitable proton source, the Fe(mnt)_2_^3−^ catalytic activity towards CO_2_R is evident, as shown in Figure 2 and Figure 5. The compound has been reported as catalytic to proton reduction,^[25]^ however it is the dianionic species that is considered HER active in analogous photocatalytic studies.^[40]^ Figure 5a shows the catalytic response of Fe(mnt)_2_ towards CO_2_R and HER with H_2_O as the proton source. Also shown is the electrochemical response in the absence of catalyst recorded at a GC electrode which indicated no significant heterogenous catalysis at the carbon electrode. In the presence of 3 M H_2_O under CO_2_ (Figure 5a) a large cathodic peak is observed at −2.15 V vs Fc which corresponds to the previously described reduction of Fe(mnt)_2_^2−^ to Fe(mnt)_2_^3−^ at the same potential (Figure 3a). However, in contrast to the reversible waveform in Figure 3a, this characteristically electrocatalytic peak generates an order of magnitude more cathodic current. This peak is therefore attributed to electrocatalytic CO_2_R because the reduced Fe(mnt)_2_^3−^ species is rapidly chemically oxidised by dissolved CO_2_ and reduced again within the interface, hence the peak displays CO_2_ diffusion limitation rather than Fe(mnt)_2_ diffusion limitation. The solubility of CO_2_ in the MeCN‐based electrolyte is approximately 0.3 M^[41]^ and far exceeds the catalyst concentration. By examining the current response under N_2_, the onset of HER is observed beyond −2.25 V vs. Fc. Therefore, a favourably high HER overpotential of approximately 300 mV exists under these conditions. This suggests that electrolysis conducted between −1.9 and −2.2 V vs. Fc should display high selectivity towards CO_2_R products and produce minimal quantities of H_2_.

Close inspection of the HER voltammogram (Figure 5a) highlights a negative shift of the Fe(mnt)_2_^3−^ oxidation peak by ∼30 mV when under N_2_ (relative to the control voltammogram in Figure 3a) which indicates a structural change in the catalyst. We attribute this to a protonated intermediate which is produced during HER and then oxidised at the electrode. To examine this further, we surveyed the catalyst response with varying concentrations of H_2_O as shown in Figure S15. Above concentrations of ∼2 M H_2_O, the two‐peak Fe(mnt)_2_^1−/2−^ voltammogram coalesces into a single redox couple, whereas at high concentrations (>5 M H_2_O) the redox chemistry significantly changes to yield multiple new oxidation and reduction peaks (Figure S16). The deterioration of the voltammetry response is exacerbated when the electrode is swept to lower vertex potentials. These effects indicate that the catalyst is decomposed somewhat by the HER reaction. Despite this, long‐term scanning (100 scans, >1 h experiment time) of the catalyst HER and CO_2_R reactions with 3 M H_2_O (shown in Figure S19) indicated good stability of the catalyst over the applied potential range. The catalytic CO_2_R peak increases with the concentration of proton donor until CO_2_ diffusion limitation is established at ∼5 M H_2_O content, however the HER overpotential continually decreases with increasing water content. We therefore conclude that a water content of ≤3 M provides a suitable balance between CO_2_R current response, HER overpotential and catalyst stability.

The catalytic activity of Fe(mnt)_2_ was also studied using TFE which possesses a relatively low pKa of 12.46 compared to water and other alcohols. As a result, the acidic proton of TFE is significantly dissociated and therefore more labile for protonation of catalytic intermediates. Figure 5b shows the HER and CO_2_R catalysis in the presence of 0.1 M TFE recorded under otherwise identical conditions. Here we observe comparable behaviour to Figure 5a with a 150 μA CO_2_R peak current at −2.20 V vs. Fc. In TFE the onset of HER occurs at less negative potentials such that the waveform differs in Figure 5a as the CO_2_R peak merges with the HER. Approximately only 200 mV of HER overpotential exists and HER begins to dominate the catalysis beyond −2.5 V vs. Fc. By varying the TFE content (Figure S18), the competing HER reaction was found to become very problematic beyond concentrations of 1 M. As such, TFE concentrations of ≤0.5 M appear most suitable for mitigating the HER. Under N_2_, the HER voltammogram shows a significant negative shift of the Fe(mnt)_2_^1−/2−^ redox couple on the second scan onwards (Figure S17). This new redox couple displays only one cathodic and anodic peak with a significantly smaller peak separation of 72 mV. Evidently, a new species is generated during the HER mechanism which displays a long lifetime and is fully reversible.

To benchmark the potential catalytic performance of the Fe(mnt)_2_ complex, cyclic voltammetry of the system was analysed using the approach outlined by Costentin and Savéant.[^42^, ^43^] This method determines the performance of molecular electrocatalysts by means of a modified Tafel analysis that evaluates the voltammetric electrocatalytic response. It was selected as a means to get a maximum turn over frequency (TOF_max_) for the complex independent of cell geometry and reaction conditions that normally influence bulk electrolysis studies. The technique accounts for the fact that the catalytic reaction rapidly turns over the complex at the electrode interface, rather than engaging all of the catalyst dissolved in the solution.

Here, the reaction pathway is assumed to be that of an EECC mechanism, as the catalytic wave is observed upon the second reduction while the first reduction remains reversible in the presence of substrate, yet both reductions are necessary to achieve the two‐electron reduction of the CO_2_. Ideally, the catalytic wave would exhibit a superimposed S‐shaped trace that is independent of scan rate, however it is common for side phenomena to add diffusion limited character. Figure S22 shows the scan rate analysis of the Fe dithiolene in an MeCN solution saturated with CO_2_ in the presence of TFE as a proton source. At high scan rates diffusion limited peak shaped character dominated the trace, while at low scan rates the trace coalesced towards an S‐shaped wave. This implies the catalytic reaction kinetics are moderate, such that a high scan rate is limited by the diffusion of the dithiolene complex, CO_2_ or proton source_,_ but at slower scan rates a steady‐state response is observed. Due to the overlap of the HER and CO_2_R processes a meaningful current plateau could not be obtained, so foot of the wave analysis (FOWA) was used to estimate the (TOF_max_).

FOWA is done using Equation 1 by plotting the diffusion normalised current against 1/(1+exp[f(E−E_1/2_)]) such that the gradient of this line can be used to determine k, which is equal to the TOF_max_ under these conditions. As with Tafel analysis, only the gradient of the initial linear portion of the line should be used to estimate the TOF_max_, where side phenomena including mass transport limitation are assumed to be negligible. Using this value, it is then possible to determine the TOF at zero overpotential (TOF_0_) so that the performance of the catalyst can be compared fairly with other catalysts. However, this requires determining the standard potential for the specific reaction taking place, and as a transient method cyclic voltammetry offers no insight into the product(s) of the reduction and so does not entirely avoid the need for bulk electrolysis.

(1) $$\frac{i}{i_{p}}=\frac{2.24n_{c}\sqrt{\frac{k}{fv}}}{1+exp\left[f\left(E-E_{\frac{1}{2}}\right)\right]}$$

Applying this methodology to data from cyclic voltammetry performed on a solution of 1 mM Fe(mnt)_2_ with 0.1 M TFE and 0.27 M CO_2_ at a scan rate of 0.1 V s^−1^ (Figure S24) yields a TOF_max_ of 21.7 s^−1^. This value is of course specific to the exact reaction conditions. The rate constant *k* is the overall rate constant for the reduction reaction(s) and cannot be deconvoluted from the substrate concentration without knowing its involvement in the rate limiting step. This is compounded when multiple products are created, each with their own rate limiting step. A relatively low turnover frequency would explain why diffusion character dominates the cyclic voltammogram at fast scan rates, as on these timescales only a small portion of the current is provided by catalytic cycling. A catalyst with a high turnover frequency, such as the widely reported Fe‐porphyrin complexes, would possess a TOF_max_ of magnitude 10^4^ s^−1^.^[44]^

### CO_2_ Electrolysis Studies

To identify the products from the Fe(mnt)_2_ catalysed CO_2_R reaction, bulk electrolysis experiments were conducted to quantify the gaseous and liquid products as a function of proton source. Representative results and analysis are shown in Figure S36 and Figure S37 for H_2_O and TFE electrolytes respectively. Here, electrolysis was conducted to a total charge threshold of 100 C and the working electrode area was adjusted as needed to produce comparable currents (∼15 mA) and total electrolysis time of approximately 1.5 to 2 h. These measures ensured that the moles of product and associated concentrations were comparable between experiments so detection and quantification would be unbiased as peaks would reside above instrument‐associated detection limits. As a further consequence, the catalyst utilisation was approximately 3.5 in each experiment; assuming no parasitic loses and two‐electron reduction in each instance (calculated by considering Faraday's law of electrolysis). Therefore, the imposed charge limit ensured that the entire quantity of catalyst was reduced at least threefold to assess stability. Critically, the catalyst persisted to completion of 100 C in each instance which proves that the catalyst is not consumed during reaction with dissolved CO_2_.

**Figure 6:**
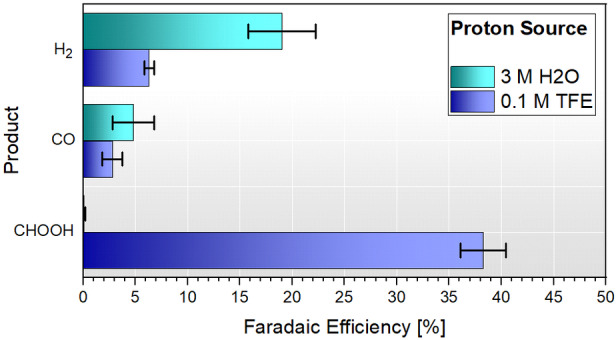
Faradaic efficiencies of CO_2_R products from electrolysis experiments using 3 M H_2_O and 100 mM TFE as proton sources. Each bar represents the mean yield of the triplicate experiment. The standard deviation of each mean result is represented by error bars.

As shown by voltammetry, reduction of Fe(mnt)_2_ can provide two‐electrons per molecule for driving catalytic reactions which suggests that the two‐electron products H_2_, CO and CHOOH will be generated. However, the possibility of further‐reduced products being generated should not be immediately ruled out. This is because cyclical regeneration of the catalyst at the electrode interface is plausible, thus resulting in further reductions of intermediates. Despite this, only H_2_, CO and CHOOH were detected in our analysis, whereas other hydrocarbons were not observed (note that the presence of oxalate was ruled out by ion chromatography). The yields of these products are shown in Figure 6, which compares the H_2_O and TFE proton sources. In both cases, electrolysis experiments gave low yields such that the total quantified faradaic efficiency was only 24±5% and 47±4% for H_2_O and TFE respectively. Evidently, the majority of charge passed is unaccounted for in our analysis which is in part attributed to uncompensated cell resistance and product loss. Indeed, achieving a gas‐tight electrolysis cell proved challenging over long‐duration experiments causing losses of gaseous products. To estimate the degree of product loss, we conducted comparable water splitting electrolysis experiments using 1 M sulfuric acid electrolyte. Here, 100 C was passed galvanostatically at 13.8 mA (corresponding to a 2 h electrolysis) giving a H_2_ faradaic yield of 67±2%. This suggests that approximately one‐third of the generated H_2_ gas was lost or remained dissolved in the electrolyte. Consequently, we speculate that gaseous yields in our catalysis experiments were higher than those measured, however this only hypothetically increases total yields to 32 and 50 % for H_2_O and TFE proton sources, respectively.

Considering the product yields in Figure 6, a clear trend is observed whereby H_2_ production is more favoured than CO production for both proton sources. This result is surprising because the applied potentials resided before the onset of HER for both proton sources as indicated in Figure 5. It is therefore evident that Fe(mnt)_2_ is an effective catalyst for HER, as noted by others.^[25]^ Indeed, by running control experiments in the absence of CO_2_, we quantify higher H_2_ yields of 31 and 23 % for H_2_O and TFE proton sources, respectively.

A more striking result shown in Figure 6 is the great disparity in CHOOH production depending on proton source. In the more acidic TFE electrolyte, CHOOH production dominates the catalysis, constituting 38±2% faradaic efficiency and an average turnover number (TON) of ∼1.3. In comparison, CHOOH was not detected when using H_2_O as the proton source. Instead, the Fe(mnt)_2_ catalyst favours H_2_ and CO production with average yields of 19±3 and 5±2% and TONs of 0.7 and 0.2 respectively. This preference towards CHOOH when using TFE was also observed by Fogeron *et al*. for the analogous nickel molybdopterin‐like dithiolene complex.^[31]^ Here they recorded CHOOH yields of 60, 30, 15 and 18 % for solutions containing TFE, H_2_O, methanol, and phenol, respectively. Their work showed that the product distribution was very dependent on the electrolyte acidity with too weakly or too strongly acidic solutions giving lower CHOOH yield than their most optimum 2 M TFE electrolyte. This therefore suggests that careful manipulation of the electrolyte acidity in the present study could in theory tune the product distribution towards higher CHOOH selectivity.

### Catalyst Stability

A catalyst solution stored within a nitrogen glovebox for six months gave near‐identical electrochemical response to that of a freshly prepared solution (Figure S21). In contrast, comparable solutions stored under ambient conditions gave completely unrecognisable voltammetry after only two weeks. This observation suggested that Fe(mnt)_2_ may be a suitable CO_2_R catalyst given that it is protected from oxygen and that the CO_2_R reaction regenerates the Fe(mnt)^1−^ species. Indeed, long term cyclic voltammetry experiments (100 scans, ∼1 h duration) showed minimal changes in current response under various conditions; without proton source under N_2_ (Figure S8), and with 3 M H_2_O (Figure S19) and 100 mM TFE (Figure S20) under N_2_ and CO_2_. These results suggested good electrochemical stability, at least under transient voltammetry conditions.

Employing Fe(mnt)_2_ in CO_2_R electrolysis experiments unfortunately highlighted additional stability issues. During the course of our studies, we noted that Fe(mnt)_2_ degrades during storage via a tendency to react with the electrolyte. This was most evident for electrolytes containing H_2_O proton source, whereby gradual discolouration of the catalyst solution (from dark brown to yellow; Figure S38) was accompanied by precipitation of a yellow‐orange solid and brown staining of the glassware that required strong acid to clean. We attribute this to oxidative decomposition of the complex that produces an insoluble iron oxide. The rate of this degradation was found to accelerate with increasing H_2_O concentration such that 7 M H_2_O solutions completely decomposed in under one day of ambient storage. In comparison, solutions containing the TFE proton source appeared to degrade no faster than those containing only supporting TBAPF_6_ electrolyte, which suggested that water‐reactivity is problematic. Despite this, electrolyte degradation during CO_2_R electrolysis was evident for both proton sources after only 2 h experiments. Electrolyte discolouration was observed for solutions containing 3 M H_2_O or 100 mM TFE, with precipitated solid observed in the post‐electrolysis H_2_O solution. In addition, the electrolysis current response typically decreased with time in all experiments, and cyclic voltammetry performed afterwards showed slightly diminished CO_2_R current response (Figure S36 and Figure S37). These observations indicated only short‐term viability of the catalyst, which limited the duration of our electrolysis experiments to ∼2 h for reliable product quantification. Interestingly, electrolysis experiments with 3 M H_2_O proton source performed under Ar gas caused the most rapid decomposition observed, with entire decomposition occurring in ∼1 h, which implies electrochemically induced decomposition via reactivity with H_2_O. Considering the high reactivity of the Fe(mnt)_2_ catalyst towards the electrolyte, H_2_O and O_2_, we speculate that the low faradaic yields of H_2_, CO and CHOOH measured are likely due to competing parasitic reactions of the catalyst. This is most likely a combination of self‐discharge; whereby the reduced catalyst is oxidised by the electrolyte solvent or supporting salt, and to a lesser extent, decomposition reactions. Here we note that cyclic voltammetry performed post‐CO_2_R electrolysis showed minimal decrease in current response which indicates that only a small portion of catalyst decomposition occurred (Figure S36b and Figure S37b).

### Mechanistic Insights

**Figure 7:**
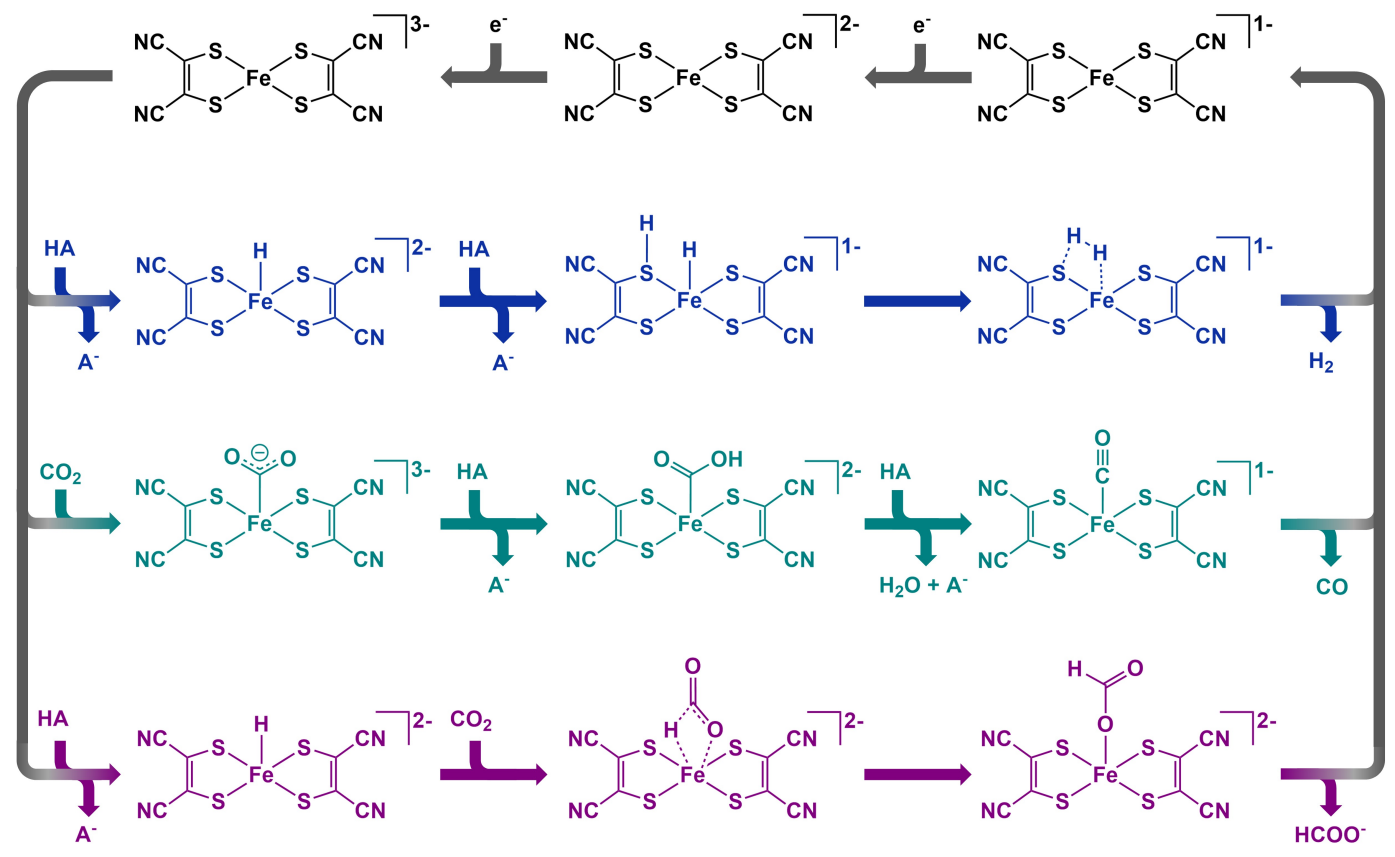
Proposed reaction mechanisms for the production of (blue) H_2_, (green) CO and (purple) CHOO^−^. HA represents a generic proton source such as H_2_O or TFE.

Based on the experimental observations and computational studies, we propose reaction mechanisms as illustrated in Figure 7, which are analogous to those of other dithiolene catalysts.^[16]^ A discussion on how the mechanism was ascertained and the corresponding energy pathways from DFT calculations are provided in the supporting information. In short, the catalytic cycles begin with the electrochemical reduction of the Fe(mnt)_2_ catalyst to form the trianionic state. This activated state then binds a proton to initiate H_2_ or CHOOH production, or CO_2_ to initiate CO production. Despite the poor ability of DFT to provide a reliable, quantitative description of the Fe(mnt)^3−^ state, qualitative analysis of this state along with quantitative analysis of the other structures proposed, allowed for the derivation of a plausible mechanistic route that reflects the product distribution in relation to proton source. Here we assume that reductions occur before bindings, however we acknowledge that these bindings may plausibly form prior to catalyst reduction. Close examination of the first voltammetry scans shown in Figure S19 and Figure S20 shows that the catalyst behaves essentially as unbound in the initial conditions, which suggests that bindings with the monoanionic state do not occur. Despite the possibility of substrate binding to the dianionic species, no catalytic current is observed until a second reduction of the catalyst has taken place; as such, pathways involving dianionic binding were not considered during this work. Though it has been recognised in other photochemical systems that the dianionic species of several other Fe‐dithiolene complexes are catalytic towards HER.^[40]^

Production of H_2_ must proceed by the binding of two protons to the catalyst within sufficient proximity to form a H_2_ molecule, as characterised by its short bond length of 0.74 Å. Therefore, the only plausible pathway proceeds by protonation of the Fe centre and one adjacent S donor. Here, the first protonation most likely proceeds at the Fe centre because the [FeH(mnt)_2_]^2−^ geometry lies 14.7 kcal mol^−1^ lower in energy compared to the [Fe(mnt)(mnt‐H)]^2−^ alternative. In addition, we compute a small intramolecular rearrangement barrier of only 2.7 kcal mol^−1^ from the [Fe(mnt)(mnt‐H)]^2−^ state which suggests facile interconversion. In both cases, the protonation induces minimal change in coordination geometry with near‐planarity maintained. Sequential protonation at a S donor then occurs in the cis conformation which lies at a negligibly higher energy compared to the trans conformation. Investigation of cis‐trans interconversion showed a significant barrier, coupled to a large geometric rearrangement, suggesting the need for dissociation, and rebinding in order to convert from a trans confirmation to a catalytically active cis geometry. We therefore speculate that trans‐deprotonation and subsequent cis‐protonation is likely more facile. Regardless, electrochemical results show that the H_2_ mechanism is facile at high overpotential which suggests that formation of the trans conformation imposes little kinetic limitation. Finally, release of H_2_ proceeds with a small barrier of 20 kcal mol^−1^.

Production of CO proceeds by direct coordination of CO_2_ to the trianionic catalyst state. In examining possible CO_2_ binding modes, we obtain and identify η^1^‐CO_2_ as the lowest energy conformation. Indeed, the η^2^‐CO_2_ and η^1^‐OCO modes could not be obtained because these structures tended to relax in energy to the η^1^‐CO_2_ geometry during optimisation. The [Fe(mnt)_2_(η^1^‐CO_2_)]^3−^ geometry is characterised by a O−C−O angle of 126° and C−O bond elongation from 1.17 to 1.26 Å indicating CO_2_ activation and resemblance with the radical anion CO_2_⋅^−^ (133° bond angle and 1.25 Å bond length). This state is then protonated to give the carboxylate intermediate [Fe(mnt)_2_(C(O)OH)]^2−^ which, in the presence of another proton, undergoes heterolytic C−O bond cleavage releasing water. This then yields [Fe(mnt)_2_(CO)]^1−^ which consequently dissociates CO with a transition state barrier of 11 kcal mol^−1^.

Production of CHOOH may occur by one of three plausible pathways: (1) formation of the intermediate [Fe(mnt)_2_(η^1^‐OCO)]^3−^ and subsequent protonation to [Fe(mnt)_2_(OCHO)]^2−^ followed by CHOO^−^ release as in Ni(cyclam);^[45]^ (2) concerted abstraction of the [FeH(mnt)_2_]^2−^ hydride by CO_2_ as in molybdpterin‐like Ni(dithiolene);^[31]^ (3) hydride insertion of CO_2_ yielding [Fe(mnt)_2_(OCHO)]^2−^ followed by CHOO^−^ release. Considering that the η^1^‐OCO adduct is energetically disfavoured compared to the η^1^‐CO_2_ mode, we conclude that pathway (1) is unlikely. Pathway (2) was investigated; however, a suitable concerted transition state was not obtained, and instead the hydride insertion transition state invoked in (3) was found to be more plausible. Therefore, while we cannot discount the plausibility of (1) and (2), we are able to note the apparent favourability of (3) in our simulations. This conclusion is consistent with our voltammetry results whereby a change of the catalyst structure is evident in the N_2_ voltammograms with H_2_O and, to a greater extent, TFE proton sources (see inset graphs in Figure 5). We speculate that this new species is [FeH(mnt)_2_]^2−^ because under CO_2_ the Fe(mnt)_2_^1−/2−^ redox couple is reminiscent of the catalyst response in the absence of proton source or CO_2_ (Figure 3). Assuming that CHOOH production proceeds via (2) or (3), then the [FeH(mnt)_2_]^2−^ species is expected to be short‐lived in the presence of CO_2_ due to hydride insertion or abstraction. Therefore, the generation of [FeH(mnt)_2_]^2−^ does not poison the catalyst towards CO_2_R but is rather the initiating reaction step for CHOOH production. Evidently, the formation of the initial [FeH(mnt)_2_]^2−^ hydride is crucial in the CHOOH mechanism. Therefore, the observed CHOOH production when using TFE is likely explained by the higher acidity of the electrolyte, which facilitates the formation of a longer‐lifetime hydride.

## Conclusions

The dithiolene complex Fe(mnt)_2_ has been evaluated as a homogeneous electrocatalyst for CO_2_R in a simple MeCN‐based electrolyte for the first time. Despite evidence of iron‐sulfur coordination complexes in CO_2_R demonstrated in the natural world, very few researchers have sought to explore the dithiolene ligand. The hitherto unexplored complex was fully voltammetrically characterised in the absence and presence of CO_2_ and proton sources. Results indicate complex interactions of the catalyst with differing electrolyte components via additional coordination at the Fe center. Indeed, we identify interesting two‐peak redox behaviour which we attribute to the existence of the dimeric [Fe_2_(mnt)_4_]^2−^ species in solution. The prevalence of this dimer appears suppressed during catalysis, however, due to catalyst protonation and CO_2_‐adduct formation.

During electrolysis, the complex typically produced two‐electron reduction products; namely H_2_, CO and CHOOH. Comparing water and TFE as proton sources, the selectivity and performance was markedly different, with the catalyst displaying a comparatively higher selectivity towards CHOOH production when using TFE, whereas none was observed in the presence of water. DFT was used to investigate the catalytic mechanism, wherein the formation of an initial hydride species was identified as critical in the production of CHOOH. This is typical of the mechanism associated with the formation of CHOOH with other homogeneous complexes. Further tuning of solution pH may therefore lead to a further increase in selectivity towards formate. This work represents a promising starting point for Fe‐based dithiolene CO_2_R catalysis, and further study of analogous derivatives.

## Experimental Section

### Chemicals and Reagents

All chemicals and reagents were used without further purification, with the exception of tetrabutylammonium hexafluorophosphate which was recrystallised from hot ethanol. Chloroacetonitrile (98 %), sodium hydroxide (pellets, 98 %), N,N‐dimethylformamide (anhydrous, amine‐free, 99.9 %), isobutanol (99 %) and 2,2,2‐tiflouroethanol (99+%) were purchased from Alfa Aesar. Sulfur (refined, 99.5 %), acetonitrile (anhydrous, extra dry, Acroseal®, 99.9+%) and tetraethylammonium chloride hydrate (99 %) were purchased from Acros Organics. Isopropanol (reagent grade, 99.5 %), ethanol (HPLC grade, 99.8 %) and iron(III) chloride (anhydrous, 99 %) were purchased from Fisher Scientific. Tetrabutylammonium hexafluorophosphate (>98 %) was purchased from Tokyo Chemical Industry UK Ltd. Ferrocene (98 %) was purchased from Sigma Aldrich.

### Synthesis of Fe(mnt)_2_

(TEA)[Fe(mnt)_2_] was prepared by use of the method published in ref^[34]^ with modifications. A full account of the synthetic method is given in the supporting information and can also be found in our previous work.^[37]^ Product analysis was performed via nuclear magnetic resonance spectroscopy, UV‐vis spectroscopy, elemental analysis, mass spectrometry, and X‐ray diffraction. Nuclear magnetic resonance (NMR) spectra were recorded on a Bruker Ultrashield 400 Plus spectrometer at 298 K. Carbon hydrogen nitrogen sulfur (CHNS) elemental analyses were performed on an Elementar vario MICRO cube. High resolution mass assignment was performed using a Shimadzu LCMS‐IT‐TOF with electrospray ionisation (ESI). UV‐vis spectrometry was performed using an Agilent Technologies Cary 60 UV‐vis spectrometer. Single crystal X‐ray diffraction analysis was performed using an Agilent Technologies SuperNova diffractometer.

### Electrochemical Methods

Electrochemical investigations were performed using either a Palmsens EmStat^3+^, Autolab PGSTAT204 or Bio‐Logic SP‐300 potentiostat. Voltammetry investigations were performed using a standard three‐electrode cell (BASI®) with an electrolyte volume of 20 mL. A glassy‐carbon (GC) macro electrode with a 7.05 mm^2^ electroactive area was employed as a working electrode (WE) whereas a platinum wire served as the counter electrode (CE). A silver wire housed in a glass‐fritted tube containing supporting electrolyte was employed as a quasi‐reference electrode (RE). Before use, a layer of AgPF_6_ was deposited on the Ag wire by oxidation in TBAPF_6_/MeCN electrolyte. The quasi‐reference was calibrated against the Ferrocene/Ferrocenium ion (Fc/Fc^+^) redox couple after voltammetry investigations by addition of Fc to the test solution. Prior to conducting voltammetry, saturation of the test solution was ensured by bubbling with either N_2_ or CO_2_ gas for 10 mins, whereas during scans, the headspace was continuously flushed. The GC WE was polished before use using two grades of diamond slurry (3 and 0.25 μM) and alumina slurry (0.05 μM) purchased from Büehler. Electrodes were then sonicated in 50 % v/v iPrOH/H_2_O solution for 5 mins before rinsing with deionised water then iPrOH and finally being air dried with compressed air.

Bulk electrolysis experiments were conducted by use of a custom H‐type glass cell comprised of two electrolyte chambers separated by a Nafion® 117 membrane. High‐purity graphite rods (Goodfellows 99.997 %) served as both the WE and CE, whereas the previously described Ag electrode served as the RE which was placed within close proximity to the WE (∼2 mm separation). The cathode compartment was filled with a 150 mL catalyst solution at 1 mM concentration which was paired with a sacrificial 100 mL Fc electrolyte at 25 mM concentration within the anode compartment. Each electrolyte contained 100 mM TBAPF_6_ supporting salt in MeCN solvent with either 100 mM TFE or 3 M H_2_O as the proton source. Prior to electrolysis, the cathode compartment was saturated with CO_2_ by bubbling through the electrolyte for 20 mins. In contrast, the anode chamber was continuously bubbled with Ar gas during electrolysis to protect the Fc^+^ ion from decomposition due to air sensitivity.^[46]^ Full schematics of the bulk electrolysis cell and photographs are given in the supporting information. Electrolysis was conducted with 85 % dynamic IR compensation (the maximum achievable without inducing potentiostat oscillation) whereas the remaining 15 % was corrected post experiment. The uncompensated resistance was measured by use of electrochemical impedance spectroscopy at the open‐circuit cell potential between 1 MHz and 100 mHz, using a 10 mV amplitude perturbation. From which, the ohmic resistance between the WE and RE was measured from the high frequency intercept in the associated Nyquist plot. The IR drop in the experiments was typically in the range of 2 to 10 Ω, corresponding to a correction of 40 to 200 mV (assuming a typical maximum current of 20 mA), respectively.

### Product Quantification

Gaseous products from CO_2_R electrolysis experiments were analysed by use of a gas chromatograph equipped with a ResTek Sin Carbon ST 80/100 column and barrier ionisation detector (Shimadzu GC‐BID 2030). The instrument was calibrated in the 0–1000 ppm range for gaseous products H_2_, CO, CH_4_ and C_2_H_4_ by use of calibration gas supplied by BOC Ltd. Calibration curves and limits of detection are given in the supporting information. Calibration samples were prepared and introduced into the GC‐BID by use of a sealed pressure vessel assembled from stainless steel fittings (Swagelok Ltd., Supporting Information). For product analysis, a gas‐tight syringe (volume 10, 50 or 100 mL. VICI® precision Sampling Ltd.) was used to sample the electrochemical headspace and dilute the sample concentration into the calibration range (typically a hundredfold dilution) using CO_2_ gas.

Liquid phase products were analysed using H^1^ NMR spectroscopy (Bruker 400 Hz) with a solvent suppression method to account for the non‐deuterated MeCN electrolyte used in electrolysis experiments. Quantification of products was achieved by use of non‐deuterated DMSO as an internal standard at 10 mM concentration; NMR samples were prepared by diluting 2 mL of a 100 mM DMSO solution in MeCN with 18 mL of post‐electrolysis Fe(mnt)_2_ catalyst solution in a 20 mL volumetric flask. The concentration of liquid phase products was then calculated on a proton basis.

### Density Functional Theory Calculations

To aid mechanistic understanding, a DFT study was conducted to obtain key reaction intermediates and transition states. Simulations were performed using the Gaussian 09 (Revision E.01) program^[47]^ with B3LYP exchange‐correlation functional[^48^, ^49^, ^50^, ^51^, ^52^] and cc‐pVDZ basis set.[^53^, ^54^] Structures were optimised using an implicit solvent model (SMD),[^55^, ^56^] for which acetonitrile (*ϵ*=35.688) was chosen as the solvent, consistent with the experiment. Minima and transition state geometries where determined through vibrational frequency analysis such that minima possess solely positive curvature and transition states showed a single vibration of negative curvature.

## Conflict of interest

The authors declare no conflict of interest.

1

## Supporting information

As a service to our authors and readers, this journal provides supporting information supplied by the authors. Such materials are peer reviewed and may be re‐organized for online delivery, but are not copy‐edited or typeset. Technical support issues arising from supporting information (other than missing files) should be addressed to the authors.

Supporting Information

## Data Availability

The data that support the findings of this study are available from the corresponding author upon reasonable request.
