# Supplementary material for: Exploring the Electrochemistry of Iron Dithiolene and Its Potential for Electrochemical Homogeneous Carbon Dioxide Reduction
Source: ChemElectroChem. 2022 Aug 4;9(17):e202200610. doi: 10.1002/celc.202200610 (PMC9546257; doi:10.1002/celc.202200610)
Supplement: Supplementary file 1 — Supporting Information [file CELC-9-0-s001.pdf]

ChemElectroChem

Supporting Information

Exploring the Electrochemistry of Iron Dithiolene and Its Potential for Electrochemical Homogeneous Carbon Dioxide Reduction

Craig G. Armstrong, Mark Potter, Thomas Malcomson, Ross W. Hogue, Sapphire M. Armstrong, Andrew Kerridge, and Kathryn E. Toghill*

Contents

1 Synthesis 2

2 X-Ray diffraction analysis 3

3 Elemental Analysis 12

4 Cyclic voltammetry 13

5 UV/vis Spectra 22

6 Glass bulk electrolysis cell design 22

7 Gas Chromatography calibrations 26

8 Bulk electrolysis 30

9 Calculation of faradaic efficiencies 32

10 Catalyst instability 34

11 Density Functional Theory 35

References 51

1 Synthesis

General synthesis details

All chemicals were used as received from the supplier without further purification. NMR spectra were recorded on a Bruker Ultrashield 400 Plus spectrometer at 298 K. CHNS elemental analyses were performed on an Elementar vario MICRO cube. High resolution mass assignment was performed using a Shimadzu LCMS-IT-TOF with electrospray ionisation (ESI).

Disodium *cis*-1,2-dicyanoethylene-1,2-dithiolate (Na_2mnt). The synthesis is adapted from that previously described,[1] with modifications. To a suspension of NaOH (9.54 g, 238 mmol, crushed into a fine powder by use of a pestle and mortar) in 30 mL DMF was slowly added sulphur (7.65 g, 238 mmol) over 5 min. The mixture was then stirred for 30 min at 100 °C, before cooling to 15 °C. A solution of chloroacetonitrile (6.05 g, 80 mmol) in 6 mL DMF was added dropwise over 20 min. The tan-brown reaction mixture was allowed to slowly warm to room temperature and was stirred for 1 h. Isobutanol (50 mL) was added and the reaction warmed to ~60°C, hot filtered, and washed with 200 mL of boiling isobutanol until a clear filtrate was obtained. To the cooled filtrate was added 100 mL of diethyl ether, and the solution was cooled to -20°C. Scratching the flask with a spatula caused crystallisation of the sodium cyanodiformate intermediate (**$NCCS_2Na{\cdot}2.5DMF$** solvate, 7.67 g, 31 %), which was isolated by filtration, washed with 100 mL diethyl ether, and air dried. $NCCS_2Na{\cdot}2.5DMF$ was dissolved in 60 mL H_2O to give a brown solution which was stirred for 48 h, in which time dimerization of the $NCCS_2Na$ intermediate results in a light brown solution with a sulphur precipitate. The mixture was filtered through celite and washed with 30 mL H_2O. The light brown filtrate was dried to a brown oil by rotary evaporator, taken up in 200 mL EtOH and again evaporated to give an orange paste. The paste was dissolved in 40 mL boiling EtOH and hot-filtered using 100 mL boiling EtOH to wash the filtered solid. The filtrate was reduced to ¼ volume by use of rotary evaporation and then 60 mL diethyl ether was added, giving a yellow/orange precipitate. The mixture was stirred at 0 °C for 30 min and then filtered to give a yellow/green powder which was dried under high-vacuum at 60 °C for 1 h, to yield **$Na_2mnt{\cdot}3H_2O$** (1.2 g, 21 %). ^{13}C NMR (D_2O, 100 MHz): δ = 126.30, 122.74. Anal calcd. for $C_4N_2Na_2S_2{\cdot}3H_2O$: C 20.00, H 2.52, N 11.66, S 26.70 %; found: C 19.89, H 2.15, N 9.67, S 22.05 %. ^{13}C NMR data was consistent with our previous work.[2]

(TEA)[Fe(mnt)$_2$]. The synthesis is adapted from that previously described[1] with modifications. To a yellow solution of $Na_2mnt{\cdot}3H_2O$ (800 mg, 4.30 mmol) in 20 mL 1:1 H_2O/EtOH was added a solution of $FeCl_3$ (341 mg, 2.10 mmol) in 6 mL H_2O resulting immediately in a dark brown-red solution. The reaction was stirred for 30 min, filtered through celite and washed with 10 mL 1:1 H_2O/EtOH. To the filtrate was added an 8 mL EtOH solution of TEACl (670 mg, 4.30 mmol) dropwise to give a dark precipitate. After stirring for 30 min, the sticky black solid mass was filtered, washed with 10 mL water then 10 mL ice cold 1:1 H_2O/EtOH, then recrystalised by dissolving in 15 mL hot acetone, adding 20 mL hot isopropanol then reducing the volume to 20 mL by heating, and cooling to 0°C. The resulting black microcrystalline solid was filtered, washed with 20 mL ice cold isopropanol, and dried in air then in vacuo to yield **(TEA)[Fe(mnt)$_2$]•2H_2O** (662 mg, 66 %). Anal calcd. for $C_{16}H_{20}FeN_5S_4{\bullet}2H_2O$: C 38.24, H 4.81, N 11.15, S 25.52 %; found: C 38.68, H 4.37, N 15.04, S 25.68 %; HRMS (ESI$^-$): m/z = 335.8354 $[Fe(mnt)_2]^-$ (calcd = 335.8361). UV-vis: λ_{max} / cm (ε / L mol^{-1} cm^{-1}) = 241.0 (19904), 270.0 (26699), 300.0 (15561), 358.5 (12060), 451.0 (9118).

2 X-Ray diffraction analysis

Experimental

Single crystals of $(TEA)_2[Fe_2(mnt)_4]$ ($C_{32}H_{40}Fe_2N_{10}S_8$) were grown by slow recrystallisation of $(TEA)[Fe(mnt)_2]\bullet 2H_2O$ from EtOH. A suitable crystal was selected and mounted on a Mitegen loop using Paratone-N oil on a SuperNova, Dual, Cu at home/near, AtlasS2 diffractometer. The crystal was kept at 200.00 K during data collection. Using Olex2,[3] the structure was solved with the SHELXT[4] structure solution program using Intrinsic Phasing and refined with the SHELXL[5] refinement package using Least Squares minimisation.

Crystal structure determination of $(TEA)_2[Fe_2(mnt)_4]$

Crystal Data for $C_{32}H_{40}Fe_2N_{10}S_8$ (M =932.92 g mol^{-1}): monoclinic, space group $P2_1/n$ (no. 14), a = 14.6221(5) Å, b = 18.4119(4) Å, c = 16.9898(5) Å, β = 112.002(3)°, V = 4240.9(2) Å^3, Z = 4, T = 200.00(10) K, μ(Cu Kα) = 9.469 mm^{-1}, $Dcalc$ = 1.461 g cm^{-3}, 15530 reflections measured (7.386° ≤ 2Θ ≤ 152.02°), 15530 unique (R_{int} = N/A, R_{sigma} = 0.0312) which were used in all calculations. The final R_1 was 0.0825 (I > 2σ(I)) and wR_2 was 0.2540 (all data).

Refinement model description

Number of restraints - 56, number of constraints - unknown. Details:

1. Twinned data refinement
Scales: 0.6116(16) 0.3884(16)

2. Fixed Uiso
At 1.2 times of: All C(H,H) groups. At 1.5 times of: All C(H,H,H) groups, All C(H,H,H,H,H,H) groups

3. Restrained distances
N10-C29A ≈ N10-C29 ≈ N10-C27 ≈ N10-C25A ≈ N10-C25 ≈ N10-C31A ≈ N10-C31 ≈ N10-C27A with sigma of 0.02. C30-C29A ≈ C30-C29 ≈ C32-C31 ≈ C32-C31A ≈ C26-C25 ≈ C26-C25A ≈ C28-C27 ≈ C28-C27A with sigma of 0.02

4. Others
Sof(H26D)=Sof(H26E)=Sof(H26F)=Sof(H28D)=Sof(H28E)=Sof(H28F)=Sof(H30D)=Sof(H30E)=Sof(H30F)=Sof(H32D)=Sof(H32E)=Sof(H32F)=Sof(C25A)=Sof(H25C)=Sof(H25D)=Sof(C27A)=Sof(H27C)=Sof(H27D)=Sof(C29A)=Sof(H29C)=Sof(H29D)=Sof(C31A)=Sof(H31C)=Sof(H31D)=1-FVAR(1)
Sof(H26A)=Sof(H26B)=Sof(H26C)=Sof(H28A)=Sof(H28B)=Sof(H28C)=Sof(H30A)=Sof(H30B)=Sof(H30C)=Sof(H32A)=Sof(H32B)=Sof(H32C)=Sof(C25)=Sof(H25A)=Sof(H25B)=Sof(C27)=Sof(H27A)=Sof(H27B)=Sof(C29)=Sof(H29A)=Sof(H29B)=Sof(C31)=Sof(H31A)=Sof(H31B)=FVAR(1)

5.a Secondary CH2 refined with riding coordinates:
C17(H17A,H17B), C19(H19A,H19B), C21(H21A,H21B), C23(H23A,H23B), C25(H25A, H25B), C27(H27A,H27B), C29(H29A,H29B), C31(H31A,H31B), C25A(H25C,H25D), C27A(H27C,H27D), C29A(H29C,H29D), C31A(H31C,H31D)

5.b Me refined with riding coordinates:
C26(H26A,H26B,H26C), C26(H26D,H26E,H26F), C28(H28A,H28B,H28C), C28(H28D,H28E,H28F), C30(H30A,H30B,H30C), C30(H30D,H30E,H30F), C32(H32A,H32B,H32C), C32(H32D,H32E,H32F)

5.c Idealised Me refined as rotating group:
C18(H18A,H18B,H18C), C20(H20A,H20B,H20C), C22(H22A,H22B,H22C), C24(H24A,H24B,H24C)

Table S 1. Crystal data and structure refinement for $(TEA)_2[Fe_2(mnt)_4]$

Identification code	**$(TEA)_2[Fe_2(mnt)_4]$**
Empirical formula	$C_{32}H_{40}Fe_2N_{10}S_8$
Formula weight	932.92
Temperature/K	200.00(10)
Crystal system	monoclinic
Space group	$P2_1/n$
a/Å	14.6221(5)
b/Å	18.4119(4)
c/Å	16.9898(5)
α/°	90
β/°	112.002(3)
γ/°	90
Volume/Å³	4240.9(2)
Z	4
$\rho_{calc}g/cm^3$	1.461
μ/mm^{-1}	9.469
F(000)	1928.0
Crystal size/mm³	0.1 × 0.08 × 0.03
Radiation	Cu Kα (λ = 1.54184)
2Θ range for data collection/°	7.386 to 152.02
Index ranges	-18 ≤ h ≤ 16, -23 ≤ k ≤ 23, -21 ≤ l ≤ 21
Reflections collected	15530
Independent reflections	15530 [R_{int} = N/A, R_{sigma} = 0.0312]
Data/restraints/parameters	15530/56/511
Goodness-of-fit on F²	1.038
Final R indexes [I>=2σ (I)]	R_1 = 0.0825, wR_2 = 0.2296
Final R indexes [all data]	R_1 = 0.1070, wR_2 = 0.2540
Largest diff. peak/hole / e Å⁻³	0.80/-0.66

Table S 2. Fractional Atomic Coordinates (×10^4) and Equivalent Isotropic Displacement Parameters ($Å^2$×10^3) for $(TEA)_2[Fe_2(mnt)_4]$. U_{eq} is defined as 1/3 of of the trace of the orthogonalised U_{IJ} tensor.

**Atom**	**x**	**y**	**z**	**U(eq)**
Fe1	1692.4(9)	5364.9(6)	5411.7(7)	28.6(3)
Fe2	3247.1(8)	4611.9(6)	4836.1(7)	28.6(3)
S1	1426.0(13)	4756.9(9)	4203.2(11)	28.5(4)
S2	1689.0(15)	6404.6(10)	4753.3(12)	34.2(4)
S3	1145.5(15)	4404.0(10)	5898.5(12)	33.8(4)
S4	1508.1(17)	6036.2(11)	6421.5(13)	39.7(5)
S5	3507.9(14)	5203.0(10)	6056.2(11)	30.2(4)
S6	3344.7(16)	3562.0(10)	5507.4(13)	39.0(5)
S7	3798.9(14)	5589.8(11)	4387.6(12)	34.2(4)
S8	3402.2(16)	3978.1(11)	3785.0(13)	39.7(5)
N1	1012(7)	5067(5)	1931(5)	58(2)
N2	1436(7)	7205(5)	2717(6)	57(2)
N3	1025(7)	3722(5)	7918(6)	61(2)
N4	1328(7)	5888(6)	8528(6)	67(3)
N5	3842(7)	4890(5)	8311(5)	57(2)
N6	3494(9)	2755(5)	7517(7)	86(4)
N7	4086(7)	6330(6)	2457(6)	65(2)
N8	3790(9)	4207(6)	1758(6)	77(3)
C1	1322(6)	5446(4)	3463(5)	32.7(16)
C2	1453(6)	6150(4)	3712(5)	31.5(16)
C3	1141(6)	5233(4)	2613(5)	37.0(18)
C4	1420(7)	6726(5)	3139(5)	40.6(19)
C5	1181(6)	4692(5)	6887(5)	33.9(17)
C6	1319(6)	5404(5)	7106(5)	39.6(19)
C7	1086(6)	4156(5)	7469(6)	42(2)
C8	1321(7)	5665(5)	7901(6)	43(2)
C9	3601(6)	4515(5)	6789(5)	34.7(17)
C10	3514(6)	3811(5)	6536(5)	37.5(18)
C11	3739(7)	4721(5)	7642(5)	43(2)
C12	3530(8)	3224(5)	7099(6)	52(2)
C13	3829(6)	5334(5)	3411(5)	37.4(18)
C14	3662(6)	4633(5)	3158(5)	37.3(18)
C15	3975(7)	5880(6)	2881(6)	46(2)
C16	3733(7)	4390(6)	2378(6)	50(2)
N9	-499(5)	6782(3)	9916(4)	31.3(14)
C17	-351(8)	6659(6)	9095(6)	55(3)
C18	-1249(9)	6853(6)	8292(6)	63(3)
C19	-589(8)	7583(5)	10041(6)	51(2)
C20	-630(7)	7805(5)	10891(6)	46(2)
C21	-1375(7)	6408(5)	9940(7)	51(2)
C22	-1389(8)	5575(5)	9860(7)	56(3)
C23	408(7)	6482(5)	10633(6)	49(2)
C24	1371(7)	6855(6)	10737(7)	59(3)
N10	5522(5)	3222(3)	10121(4)	36.9(15)
C26	6492(8)	4215(6)	9721(7)	65(3)
C28	3706(7)	3401(7)	9849(8)	71(3)
C30	6019(9)	2364(6)	9164(7)	69(3)
C32	5815(10)	2935(7)	11683(7)	75(4)
C25	6479(9)	3624(7)	10380(8)	43(3)
C27	4736(9)	3775(6)	10115(9)	45(3)
C29	5203(10)	2857(7)	9288(8)	48(4)
C31	5618(12)	2651(8)	10798(9)	58(4)
C25A	5464(14)	3855(10)	9519(14)	35(6)
C27A	4535(12)	2858(10)	9762(17)	37(6)
C29A	6315(16)	2700(12)	10055(12)	43(6)
C31A	5840(20)	3487(12)	11005(10)	45(7)

Table S 3. Anisotropic Displacement Parameters ($Å^2×10^3$) for $(TEA)_2[Fe_2(mnt)_4]$. The Anisotropic displacement factor exponent takes the form: $-2\pi^2[h^2a^{*2}U_{11}+2hka^*b^*U_{12}+…]$.

Atom	U_{11}	U_{22}	U_{33}	U_{23}	U_{13}
Fe1	31.8(7)	25.5(6)	28.5(6)	-1.2(4)	11.3(5)
Fe2	27.7(6)	27.8(6)	28.1(6)	-2.7(4)	8.1(5)
S1	29.5(9)	26.1(8)	28.6(8)	-2.8(6)	9.3(7)
S2	41.3(11)	24.6(8)	34.4(9)	-0.6(7)	11.3(8)
S3	35.7(10)	30.7(9)	34.7(9)	-1.0(7)	12.8(8)
S4	53.2(13)	30.8(9)	40.8(10)	-6.4(8)	24.1(10)
S5	30.2(10)	30.5(9)	27.4(8)	-2.6(7)	7.8(7)
S6	48.7(12)	26.7(9)	37.1(10)	-0.3(7)	10.8(9)
S7	30.3(10)	37.7(10)	35.3(9)	-3.8(8)	13.0(8)
S8	44.8(12)	36.3(10)	39.7(10)	-7.3(8)	17.7(9)
N1	78(6)	54(5)	38(4)	-4(4)	16(4)
N2	64(6)	47(5)	52(5)	13(4)	12(4)
N3	76(6)	58(5)	51(5)	16(4)	27(5)
N4	70(6)	90(7)	46(5)	-12(5)	28(5)
N5	68(6)	66(6)	33(4)	-2(4)	12(4)
N6	121(10)	55(6)	61(6)	25(5)	11(6)
N7	57(6)	80(7)	59(5)	19(5)	24(5)
N8	100(8)	85(7)	57(6)	-19(5)	42(6)
C1	28(4)	43(4)	25(3)	-3(3)	8(3)
C2	29(4)	25(3)	39(4)	4(3)	13(3)
C3	39(5)	34(4)	33(4)	-1(3)	8(3)
C4	41(5)	37(4)	36(4)	4(4)	5(4)
C5	25(4)	50(5)	29(4)	0(3)	13(3)
C6	34(5)	51(5)	36(4)	-3(4)	16(3)
C7	35(5)	53(5)	39(4)	-1(4)	16(4)
C8	36(5)	54(5)	40(5)	-5(4)	15(4)
C9	27(4)	45(4)	27(4)	-1(3)	4(3)
C10	27(4)	44(5)	36(4)	8(3)	5(3)
C11	46(5)	41(5)	32(4)	0(3)	1(4)
C12	58(6)	43(5)	42(5)	9(4)	4(4)
C13	25(4)	56(5)	34(4)	0(4)	14(3)
C14	24(4)	56(5)	29(4)	-3(3)	8(3)
C15	36(5)	59(6)	41(5)	0(4)	13(4)
C16	44(5)	63(6)	44(5)	-10(4)	16(4)
N9	30(3)	32(3)	34(3)	6(3)	15(3)
C17	67(7)	58(6)	49(5)	10(5)	34(5)
C18	76(8)	71(7)	36(5)	8(5)	15(5)
C19	53(6)	40(5)	56(6)	6(4)	17(5)
C20	52(6)	38(5)	46(5)	-3(4)	17(4)
C21	46(6)	55(6)	60(6)	5(5)	28(5)
C22	71(7)	38(5)	62(6)	-6(4)	29(5)
C23	54(6)	42(5)	51(5)	11(4)	20(5)
C24	40(6)	73(7)	61(6)	4(5)	17(5)
N10	32(4)	28(3)	44(4)	4(3)	8(3)
C26	61(7)	71(7)	62(6)	5(5)	23(5)
C28	34(6)	93(9)	87(9)	14(7)	24(6)
C30	79(8)	55(6)	63(7)	-4(5)	18(6)
C32	86(9)	74(8)	63(7)	29(6)	25(7)
C25	35(7)	47(8)	48(7)	-1(6)	14(6)
C27	54(9)	33(7)	50(8)	5(6)	21(7)
C29	49(8)	36(7)	51(8)	-8(6)	9(6)
C31	42(8)	53(9)	71(10)	21(8)	13(7)
C25A	40(14)	24(11)	39(12)	7(9)	13(10)
C27A	20(12)	28(12)	62(16)	11(10)	14(11)
C29A	48(16)	32(13)	49(15)	17(11)	19(12)
C31A	38(15)	48(16)	44(14)	10(12)	9(12)

Table S 4. Bond Lengths for $(TEA)_2[Fe_2(mnt)_4]$.

Atom	*Atom*	Length [Å]	*Atom*	*Atom*	Length [Å]
Fe1	*S1*	2.241(2)	*C6*	*C8*	1.433(12)
Fe1	*S2*	2.216(2)	*C9*	*C10*	1.357(12)
Fe1	*S3*	2.225(2)	*C9*	*C11*	1.438(11)
Fe1	*S4*	2.211(2)	*C10*	*C12*	1.438(12)
Fe1	*S5*	2.481(2)	*C13*	*C14*	1.354(12)
Fe2	*S1*	2.485(2)	*C13*	*C15*	1.417(13)
Fe2	*S5*	2.244(2)	*C14*	*C16*	1.439(12)
Fe2	*S6*	2.222(2)	*N9*	*C17*	1.507(11)
Fe2	*S7*	2.221(2)	*N9*	*C19*	1.502(11)
Fe2	*S8*	2.215(2)	*N9*	*C21*	1.469(11)
S1	*C1*	1.752(8)	*N9*	*C23*	1.529(11)
S2	*C2*	1.735(8)	*C17*	*C18*	1.540(14)
S3	*C5*	1.744(8)	*C19*	*C20*	1.524(13)
S4	*C6*	1.739(9)	*C21*	*C22*	1.539(13)
S5	*C9*	1.745(8)	*C23*	*C24*	1.516(13)
S6	*C10*	1.732(9)	*N10*	*C25*	1.497(11)
S7	*C13*	1.741(8)	*N10*	*C27*	1.533(12)
S8	*C14*	1.743(9)	*N10*	*C29*	1.476(12)
N1	*C3*	1.145(11)	*N10*	*C31*	1.526(12)
N2	*C4*	1.141(12)	*N10*	*C25A*	1.531(15)
N3	*C7*	1.129(12)	*N10*	*C27A*	1.497(15)
N4	*C8*	1.138(12)	*N10*	*C29A*	1.543(16)
N5	*C11*	1.132(12)	*N10*	*C31A*	1.479(17)
N6	*C12*	1.132(13)	*C26*	*C25*	1.567(13)
N7	*C15*	1.149(13)	*C26*	*C25A*	1.558(17)
N8	*C16*	1.138(13)	*C28*	*C27*	1.561(13)
C1	*C2*	1.354(11)	*C28*	*C27A*	1.620(17)
C1	*C3*	1.421(11)	*C30*	*C29*	1.573(14)
C2	*C4*	1.429(11)	*C30*	*C29A*	1.538(18)
C5	*C6*	1.357(12)	*C32*	*C31*	1.515(15)
C5	*C7*	1.441(12)	*C32*	*C31A*	1.546(17)

Table S 5. Bond Angles for $(TEA)_2[Fe_2(mnt)_4]$.

Atom	*Atom*	*Atom*	Angle [˚]	*Atom*	*Atom*	*Atom*	Angle [˚]
S1	*Fe1*	*S5*	97.72(8)	*N4*	*C8*	*C6*	178.4(12)
S2	*Fe1*	*S1*	90.13(8)	*C10*	*C9*	*S5*	119.6(6)
S2	*Fe1*	*S3*	160.29(10)	*C10*	*C9*	*C11*	122.2(8)
S2	*Fe1*	*S5*	97.25(8)	*C11*	*C9*	*S5*	118.2(6)
S3	*Fe1*	*S1*	88.86(8)	*C9*	*C10*	*S6*	122.3(6)
S3	*Fe1*	*S5*	102.39(8)	*C9*	*C10*	*C12*	122.1(8)
S4	*Fe1*	*S1*	163.78(10)	*C12*	*C10*	*S6*	115.6(7)
S4	*Fe1*	*S2*	86.04(8)	*N5*	*C11*	*C9*	179.2(11)
S4	*Fe1*	*S3*	89.48(9)	*N6*	*C12*	*C10*	176.5(12)
S4	*Fe1*	*S5*	98.39(8)	*C14*	*C13*	*S7*	119.6(7)
S5	*Fe2*	*S1*	97.55(8)	*C14*	*C13*	*C15*	121.8(8)
S6	*Fe2*	*S1*	99.67(8)	*C15*	*C13*	*S7*	118.5(7)
S6	*Fe2*	*S5*	89.52(8)	*C13*	*C14*	*S8*	121.4(6)
S7	*Fe2*	*S1*	103.45(8)	*C13*	*C14*	*C16*	121.5(9)
S7	*Fe2*	*S5*	88.05(8)	*C16*	*C14*	*S8*	117.1(7)
S7	*Fe2*	*S6*	156.87(10)	*N7*	*C15*	*C13*	179.0(11)
S8	*Fe2*	*S1*	97.16(8)	*N8*	*C16*	*C14*	179.1(13)
S8	*Fe2*	*S5*	165.27(9)	*C17*	*N9*	*C23*	107.5(7)
S8	*Fe2*	*S6*	87.09(9)	*C19*	*N9*	*C17*	109.3(7)
S8	*Fe2*	*S7*	89.45(9)	*C19*	*N9*	*C23*	109.7(7)
Fe1	*S1*	*Fe2*	82.35(7)	*C21*	*N9*	*C17*	112.6(8)
C1	*S1*	*Fe1*	103.6(3)	*C21*	*N9*	*C19*	109.4(7)
C1	*S1*	*Fe2*	100.3(3)	*C21*	*N9*	*C23*	108.3(7)
C2	*S2*	*Fe1*	104.1(3)	*N9*	*C17*	*C18*	114.5(8)
C5	*S3*	*Fe1*	103.4(3)	*N9*	*C19*	*C20*	115.8(8)
C6	*S4*	*Fe1*	103.9(3)	*N9*	*C21*	*C22*	116.5(8)
Fe2	*S5*	*Fe1*	82.36(7)	*C24*	*C23*	*N9*	114.8(8)
C9	*S5*	*Fe1*	100.7(3)	*C25*	*N10*	*C27*	106.9(8)
C9	*S5*	*Fe2*	104.4(3)	*C25*	*N10*	*C31*	107.9(8)
C10	*S6*	*Fe2*	104.2(3)	*C29*	*N10*	*C25*	115.6(9)
C13	*S7*	*Fe2*	104.5(3)	*C29*	*N10*	*C27*	109.3(8)
C14	*S8*	*Fe2*	103.9(3)	*C29*	*N10*	*C31*	108.4(9)
C2	*C1*	*S1*	120.3(6)	*C31*	*N10*	*C27*	108.7(9)
C2	*C1*	*C3*	122.1(7)	*C25A*	*N10*	*C29A*	106.3(13)
C3	*C1*	*S1*	117.5(6)	*C27A*	*N10*	*C25A*	105.4(12)
C1	*C2*	*S2*	121.9(6)	*C27A*	*N10*	*C29A*	109.1(12)
C1	*C2*	*C4*	122.2(8)	*C31A*	*N10*	*C25A*	110.3(12)
C4	*C2*	*S2*	115.9(6)	*C31A*	*N10*	*C27A*	116.4(15)
N1	*C3*	*C1*	178.8(11)	*C31A*	*N10*	*C29A*	108.9(14)
N2	*C4*	*C2*	176.2(10)	*N10*	*C25*	*C26*	113.1(9)
C6	*C5*	*S3*	120.3(6)	*N10*	*C27*	*C28*	110.4(9)
C6	*C5*	*C7*	121.1(8)	*N10*	*C29*	*C30*	114.1(10)
C7	*C5*	*S3*	118.5(7)	*C32*	*C31*	*N10*	116.1(10)
C5	*C6*	*S4*	120.5(6)	*N10*	*C25A*	*C26*	111.7(12)
C5	*C6*	*C8*	122.0(8)	*N10*	*C27A*	*C28*	109.1(12)
C8	*C6*	*S4*	117.5(7)	*C30*	*C29A*	*N10*	112.3(13)
N3	*C7*	*C5*	178.1(10)	*N10*	*C31A*	*C32*	117.1(14)

Table S 6. Torsion Angles for $(TEA)_2[Fe_2(mnt)_4]$.

A	B	C	D	Angle [˚]	A	B	C	D	Angle [˚]
Fe1	S1	C1	C2	3.1(7)	C17	N9	C19	C20	-173.6(8)
Fe1	S1	C1	C3	-179.6(6)	C17	N9	C21	C22	60.8(11)
Fe1	S2	C2	C1	-0.2(7)	C17	N9	C23	C24	62.5(10)
Fe1	S2	C2	C4	-179.4(6)	C19	N9	C17	C18	-67.4(11)
Fe1	S3	C5	C6	12.2(7)	C19	N9	C21	C22	-177.5(9)
Fe1	S3	C5	C7	-166.3(6)	C19	N9	C23	C24	-56.2(10)
Fe1	S4	C6	C5	-8.8(8)	C21	N9	C17	C18	54.4(11)
Fe1	S4	C6	C8	170.8(6)	C21	N9	C19	C20	62.7(10)
Fe1	S5	C9	C10	85.0(7)	C21	N9	C23	C24	-175.6(8)
Fe1	S5	C9	C11	-93.2(7)	C23	N9	C17	C18	173.5(9)
Fe2	S1	C1	C2	-81.4(7)	C23	N9	C19	C20	-55.9(10)
Fe2	S1	C1	C3	95.8(6)	C23	N9	C21	C22	-57.9(11)
Fe2	S5	C9	C10	0.3(7)	C25	N10	C27	C28	180.0(10)
Fe2	S5	C9	C11	-178.0(6)	C25	N10	C29	C30	50.5(14)
Fe2	S6	C10	C9	-2.6(8)	C25	N10	C31	C32	61.5(14)
Fe2	S6	C10	C12	175.6(6)	C27	N10	C25	C26	-65.1(13)
Fe2	S7	C13	C14	-8.2(7)	C27	N10	C29	C30	171.0(10)
Fe2	S7	C13	C15	168.4(6)	C27	N10	C31	C32	-54.0(14)
Fe2	S8	C14	C13	7.0(7)	C29	N10	C25	C26	56.7(14)
Fe2	S8	C14	C16	-175.5(6)	C29	N10	C27	C28	54.3(13)
S1	C1	C2	S2	-2.1(10)	C29	N10	C31	C32	-172.6(12)
S1	C1	C2	C4	177.1(6)	C31	N10	C25	C26	178.2(11)
S3	C5	C6	S4	-2.4(10)	C31	N10	C27	C28	-63.9(12)
S3	C5	C6	C8	178.0(7)	C31	N10	C29	C30	-70.7(13)
S5	C9	C10	S6	1.7(10)	C25A	N10	C27A	C28	-67.4(18)
S5	C9	C10	C12	-176.5(7)	C25A	N10	C29A	C30	-64.5(19)
S7	C13	C14	S8	0.8(10)	C25A	N10	C31A	C32	173.1(17)
S7	C13	C14	C16	-176.6(7)	C27A	N10	C25A	C26	-170.8(15)
C3	C1	C2	S2	-179.2(6)	C27A	N10	C29A	C30	49(2)
C3	C1	C2	C4	0.0(13)	C27A	N10	C31A	C32	53(2)
C7	C5	C6	S4	176.0(6)	C29A	N10	C25A	C26	-55.1(18)
C7	C5	C6	C8	-3.6(13)	C29A	N10	C27A	C28	178.9(14)
C11	C9	C10	S6	179.8(7)	C29A	N10	C31A	C32	-71(2)
C11	C9	C10	C12	1.7(14)	C31A	N10	C25A	C26	63(2)
C15	C13	C14	S8	-175.6(7)	C31A	N10	C27A	C28	55(2)
C15	C13	C14	C16	7.0(13)	C31A	N10	C29A	C30	176.8(15)

Table S 7. Hydrogen Atom Coordinates (Å×10^4) and Isotropic Displacement Parameters (Å^2×10^3) for $(TEA)_2[Fe_2(mnt)_4]$.

Atom	x	y	z	U(eq)
H17A	-185	6141	9063	66
H17B	220	6952	9102	66
H18A	-1080	6786	7790	94
H18B	-1434	7360	8324	94
H18C	-1803	6535	8250	94
H19A	-1194	7761	9583	61
H19B	-21	7831	9976	61
H20A	-690	8335	10910	69
H20B	-25	7648	11352	69
H20C	-1201	7577	10959	69
H21A	-1962	6603	9475	61
H21B	-1445	6533	10481	61
H22A	-1217	5438	9375	84
H22B	-2049	5393	9773	84
H22C	-908	5365	10380	84
H23A	301	6525	11173	59
H23B	469	5959	10529	59
H24A	1464	6846	10195	88
H24B	1919	6599	11169	88
H24C	1353	7359	10914	88
H26A	7134	4460	9924	97
H26B	6378	3984	9173	97
H26C	5971	4573	9654	97
H26D	6428	4617	9325	97
H26E	6740	4402	10304	97
H26F	6954	3854	9662	97
H28A	3212	3760	9848	107
H28B	3524	3194	9279	107
H28C	3737	3013	10253	107
H28D	3059	3166	9615	107
H28E	3865	3515	10449	107
H28F	3693	3850	9535	107
H30A	5755	2141	8600	103
H30B	6595	2662	9217	103
H30C	6213	1983	9598	103
H30D	6542	2037	9152	103
H30E	5404	2090	9028	103
H30F	5921	2752	8745	103
H32A	5864	2526	12066	113
H32B	6436	3208	11886	113
H32C	5274	3254	11670	113
H32D	6037	3173	12239	113
H32E	5141	2754	11538	113
H32F	6255	2527	11703	113
H25A	7017	3272	10455	52
H25B	6611	3861	10935	52
H27A	4704	4172	9711	54
H27B	4917	3991	10687	54
H29A	5000	3231	8836	57
H29B	4619	2554	9217	57
H31A	5002	2363	10612	70
H31B	6160	2317	10828	70
H25C	4996	4223	9569	42
H25D	5210	3675	8926	42
H27C	4548	2402	10074	45
H27D	4376	2737	9157	45
H29C	6943	2967	10193	51

H29D	6426	2306	10477	51
H31C	5407	3901	11012	55
H31D	6516	3676	11176	55

Table S 8. Atomic Occupancy for $(TEA)_2[Fe_2(mnt)_4]$.

Atom	Occupancy	*Atom*	Occupancy	*Atom*	Occupancy
H26A	0.666(11)	*H26B*	0.666(11)	*H26C*	0.666(11)
H26D	0.334(11)	*H26E*	0.334(11)	*H26F*	0.334(11)
H28A	0.666(11)	*H28B*	0.666(11)	*H28C*	0.666(11)
H28D	0.334(11)	*H28E*	0.334(11)	*H28F*	0.334(11)
H30A	0.666(11)	*H30B*	0.666(11)	*H30C*	0.666(11)
H30D	0.334(11)	*H30E*	0.334(11)	*H30F*	0.334(11)
H32A	0.666(11)	*H32B*	0.666(11)	*H32C*	0.666(11)
H32D	0.334(11)	*H32E*	0.334(11)	*H32F*	0.334(11)
C25	0.666(11)	*H25A*	0.666(11)	*H25B*	0.666(11)
C27	0.666(11)	*H27A*	0.666(11)	*H27B*	0.666(11)
C29	0.666(11)	*H29A*	0.666(11)	*H29B*	0.666(11)
C31	0.666(11)	*H31A*	0.666(11)	*H31B*	0.666(11)
C25A	0.334(11)	*H25C*	0.334(11)	*H25D*	0.334(11)
C27A	0.334(11)	*H27C*	0.334(11)	*H27D*	0.334(11)
C29A	0.334(11)	*H29C*	0.334(11)	*H29D*	0.334(11)
C31A	0.334(11)	*H31C*	0.334(11)	*H31D*	0.334(11)

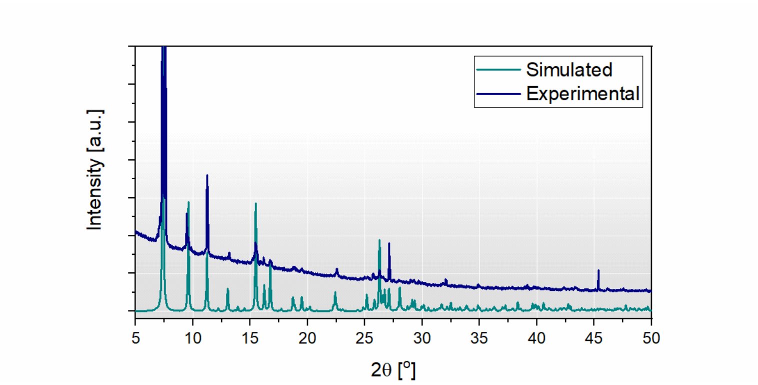

Figure S 1. Comparison of the experimentally measured powder X-ray diffraction pattern with the simulated pattern calculated by use of the parameters obtained from single-crystal XRD analysis.

3 Elemental Analysis

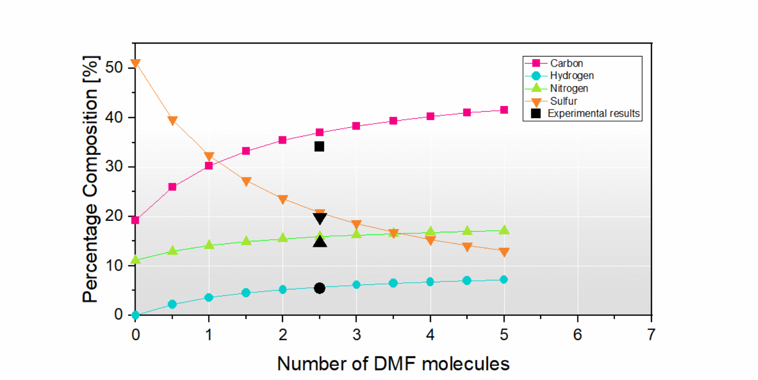

Figure S 2. Elemental analysis of $NCCS_2Na$ intermediate. The experimentally determined CHNS content (black symbols) is compared to calculated compositions of the intermediate with varying numbers of stoichiometric DMF molecules.

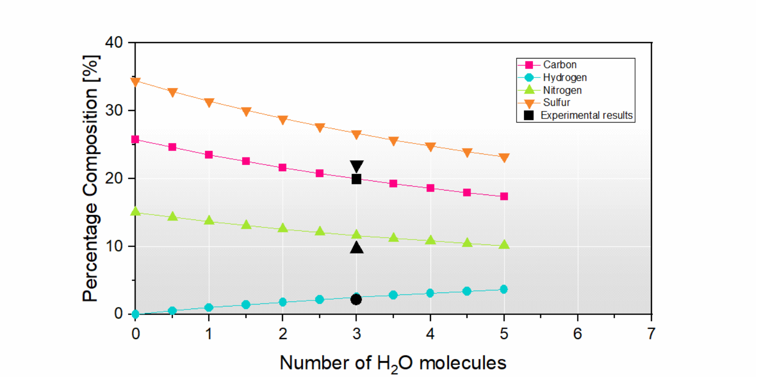

Figure S 3. Elemental analysis of Na_2mnt ligand. The experimentally determined CHNS content (black symbols) is compared to calculated compositions of the ligand with varying numbers of stoichiometric H_2O molecules.

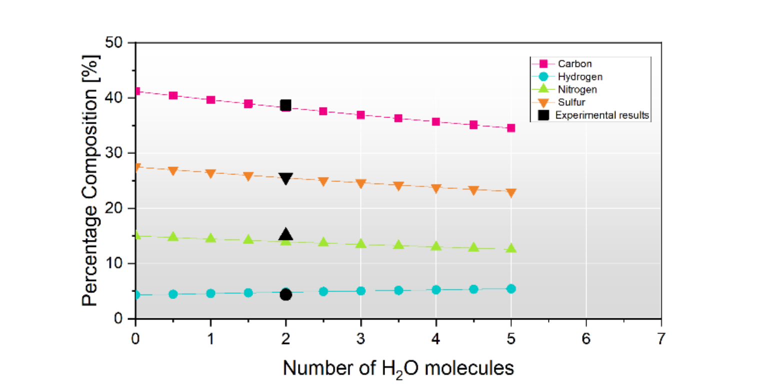

Figure S 4. Elemental analysis of (TEA)[Fe(mnt)$_2$] complex. The experimentally determined CHNS content (black symbols) is compared to calculated compositions of the ligand with varying numbers of stoichiometric H_2O molecules.

4 Cyclic voltammetry

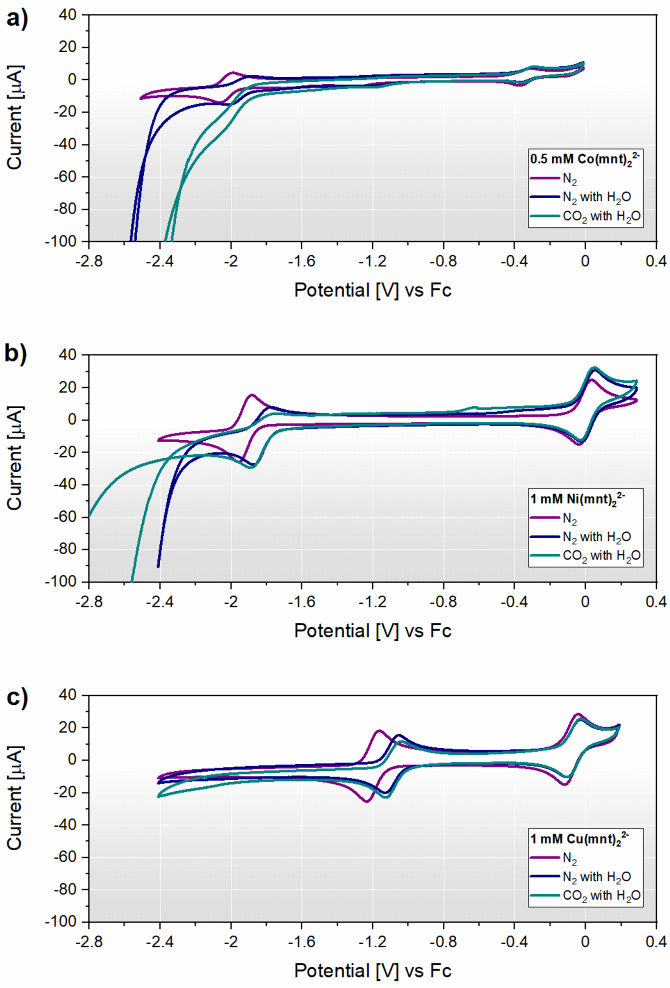

Figure S 5. Cyclic voltammetry of a) 0.5 mM $(TEA)_2[Co(mnt)_2]$, b) 1 mM $(TEA)_2[Ni(mnt)_2]$ and c) 1 mM $(TEA)_2[Cu(mnt)_2]$ in 100 mM $TBAPF_6$ MeCN electrolyte, under different conditions. The electrochemical response in the absence of water proton source (purple) is compared to solutions containing 5.1 M H_2O under N_2 (navy) and CO_2 (teal). Hydrous electrolytes were prepared by addition of 1 mL of H_2O to 10 mL electrolytes causing dilution of the complexes to 0.45, 0.91 and 0.91 mM, respectively, and $TBAPF_6$ dilution to 91 mM. Second scans shown at 100 mV s^{-1}.

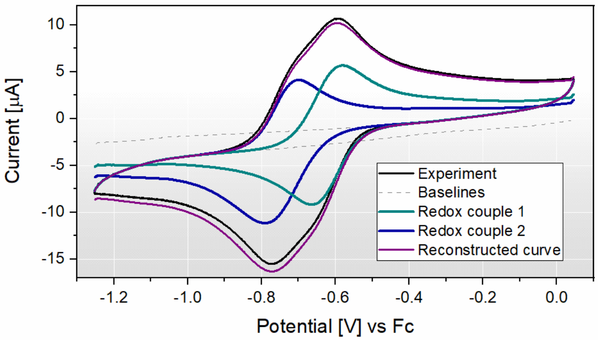

Figure S 6. Cyclic voltammetry of the $Fe(mnt)_2^{1-/2-}$ redox couple (black) at 100 mV s^{-1} at a GC electrode, under N_2 (second scan shown). Peak deconvolution analysis of the voltammogram into two diffusion-limited redox couples is shown. The

reconstructed curve is also given as a comparison to the experimental data for quality indication. The electrolyte was composed of 1 mM (TEA)[Fe(mnt)$_2$] with 100 mM TBAPF$_6$ supporting electrolyte in MeCN solvent.

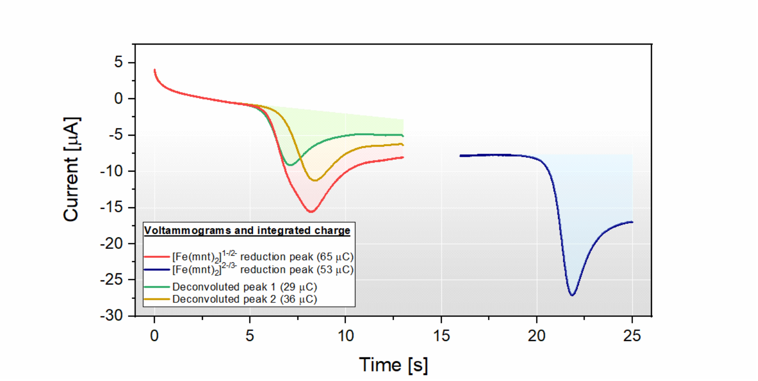

Figure S 7. Peak integration of the [Fe(mnt)$_2$]$^{1-/2-}$ and [Fe(mnt)$_2$]$^{2-/3-}$ and cathodic peaks. The deconvoluted peaks of the [Fe(mnt)$_2$]$^{1-/2-}$ waveform are also shown with corresponding charges given for comparison. Voltammetry recorded at 100 mV s^{-1} at a GC electrode, under N$_2$. The electrolyte was composed of 1 mM (TEA)[Fe(mnt)$_2$] with 100 mM TBAPF$_6$ supporting electrolyte in MeCN solvent.

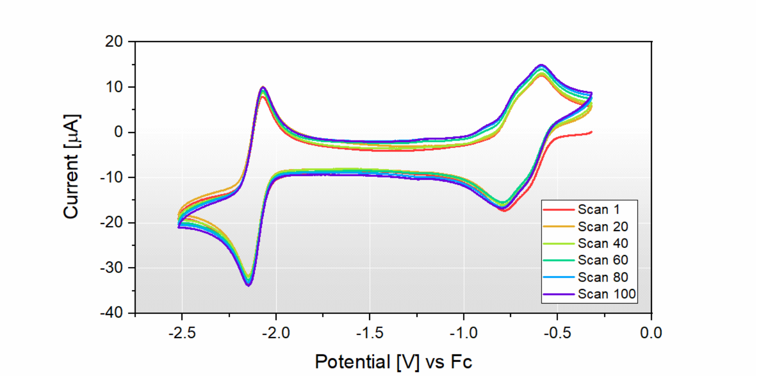

Figure S 8. 100 voltammetry scans of the Fe(mnt)$_2$ reversible redox couples recorded at 100 mV s^{-1} at a GC electrode, under N$_2$. The electrolyte was composed of 1 mM (TEA)[Fe(mnt)$_2$] with 100 mM TBAPF$_6$ supporting electrolyte in MeCN solvent. The total experiment time was 1h13min.

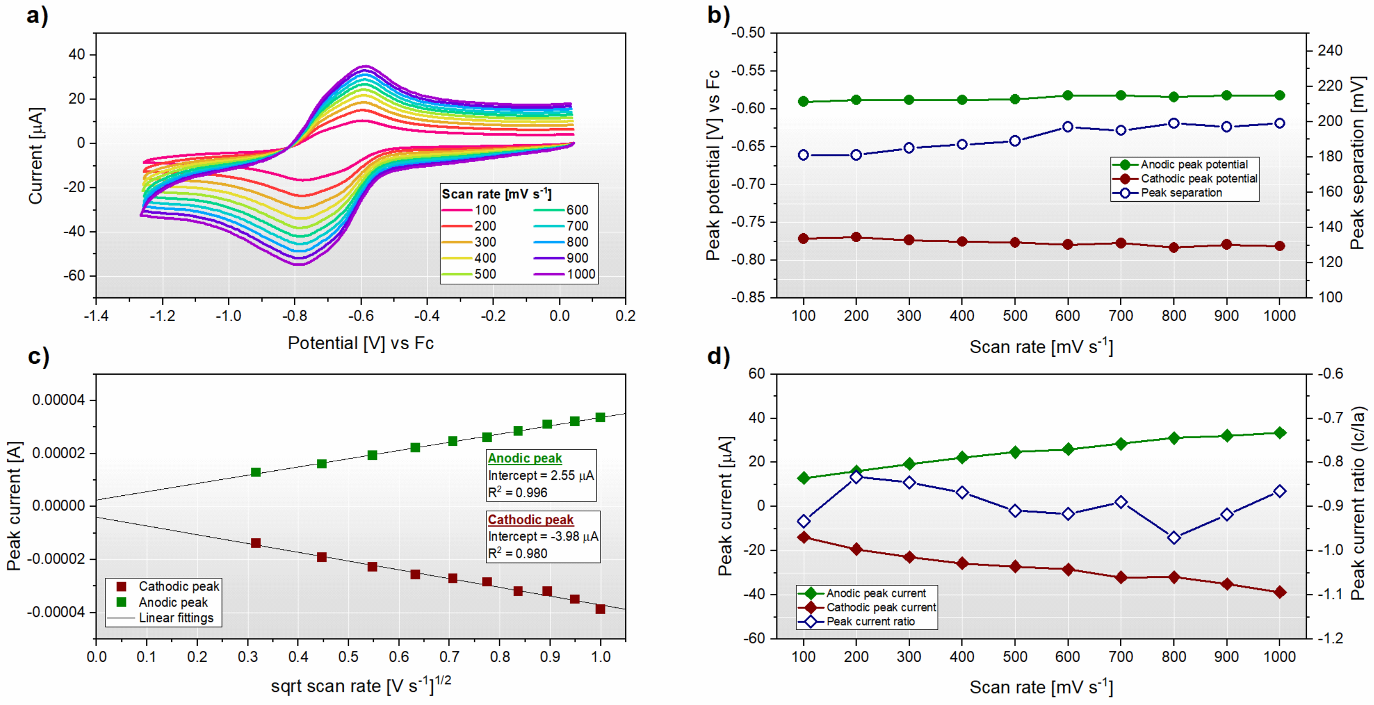

Figure S 9. Peak analysis of the $Fe(mnt)_2^{1-/2-}$ redox couple as a function of scan rate in the range of 0.1 to 1 V s^{-1}. a) Cyclic voltammograms recorded under N_2 at a GC electrode. First scans are shown. b) Peak potentials and peak separation. c) Randles-Sevcik analysis. d) Peak currents and ratio. The electrolyte was composed of 1 mM (TEA)[$Fe(mnt)_2$] with 100 mM $TBAPF_6$ in MeCN.

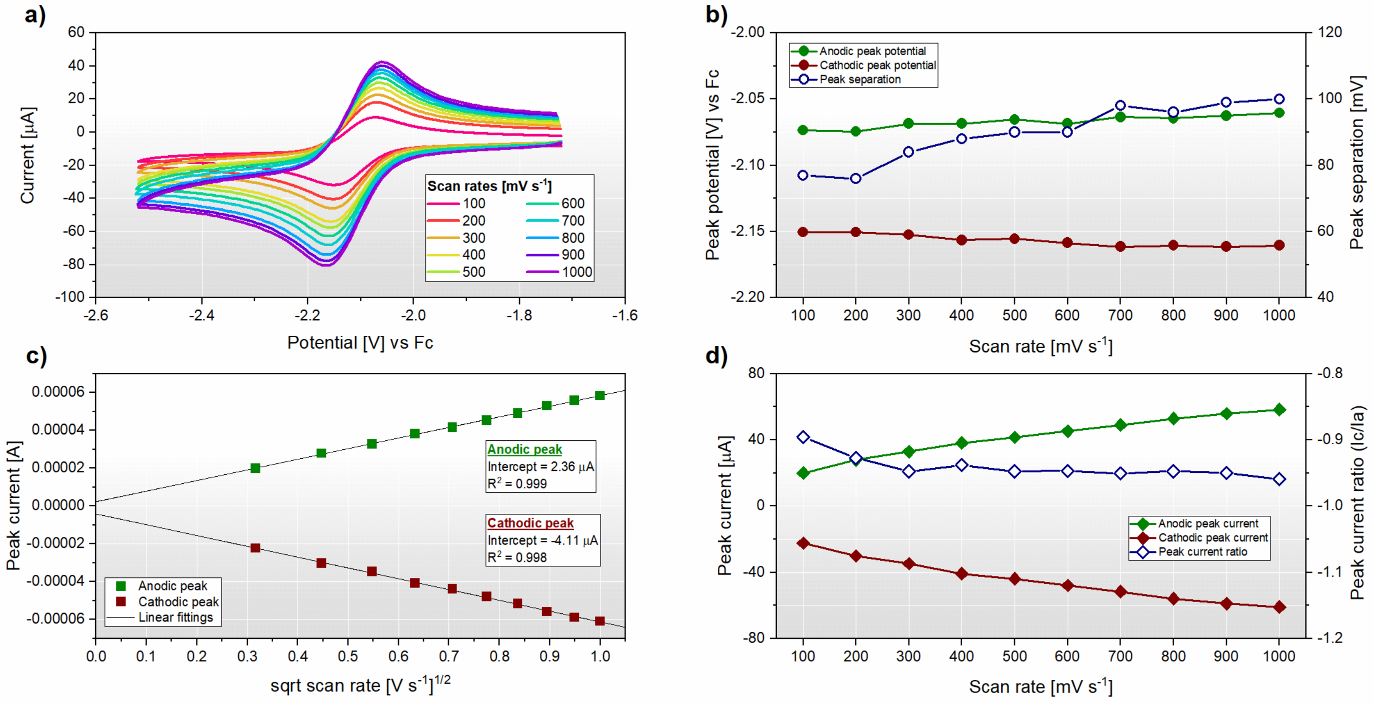

Figure S 10. Peak analysis of the $Fe(mnt)_2^{2-/3-}$ redox couple as a function of scan rate in the range of 0.1 to 1 V s^{-1}. a) Cyclic voltammograms recorded under N_2 at a GC electrode. First scans are shown. b) Peak potentials and peak separation. c) Randles-Sevcik analysis. d) Peak currents and ratio. The electrolyte was composed of 1 mM (TEA)[$Fe(mnt)_2$] with 100 mM $TBAPF_6$ in MeCN.

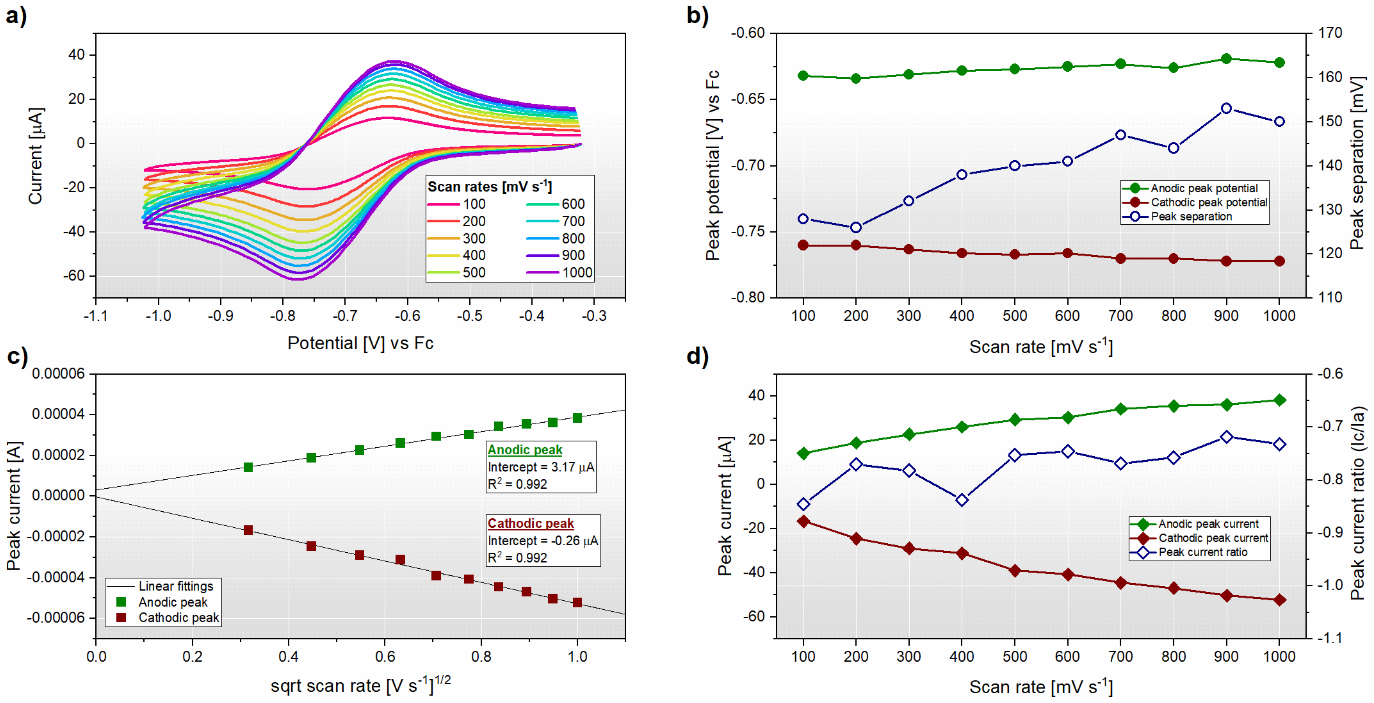

Figure S 11. Peak analysis of the $Fe(mnt)_2^{1-/2-}$ redox couple in hydrous electrolyte as a function of scan rate in the range of 0.1 to 1 V s^{-1}. a) Cyclic voltammograms recorded under N_2 at a GC electrode. First scans are shown. b) Peak potentials and peak separation. c) Randles-Sevcik analysis. d) Peak currents and ratio. The electrolyte was composed of 1 mM (TEA)[$Fe(mnt)_2$] with 3 M H_2O and 100 mM $TBAPF_6$ in MeCN.

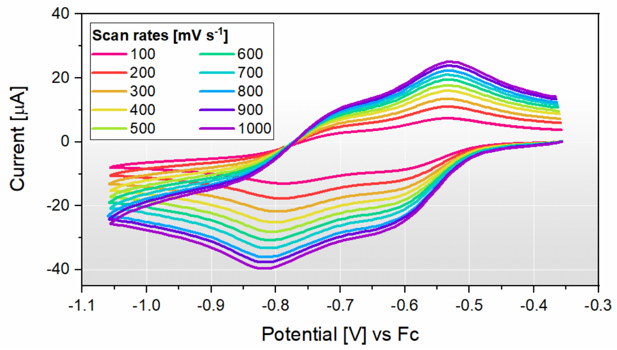

Figure S 12. Scan rate dependence of the $Fe(mnt)_2^{1-/2-}$ redox couple at a GC electrode under N_2 in concentrated TFE electrolyte. The electrolyte was composed of 1 mM (TEA)[$Fe(mnt)_2$] with 5 M TFE and 100 mM $TBAPF_6$ in MeCN. First scans are shown.

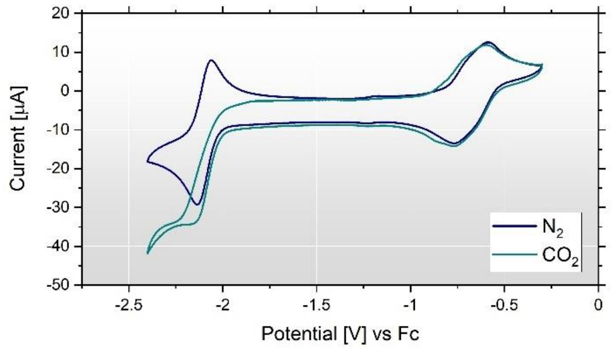

Figure S 13. Cyclic voltammetry of $Fe(mnt)_2^{1-}$ as a function of saturated gas (N_2 and CO_2) at a GC electrode. The electrolytes were composed of 1 mM (TEA)[$Fe(mnt)_2$] with 0.1 M $TBAPF_6$ in MeCN. Second scans shown at 100 mV s^{-1}. As with all electrolytes studied, the solution possessed at minimum 2 mM of water originating from the synthesised catalyst and hygroscopic electrolyte and solvent.

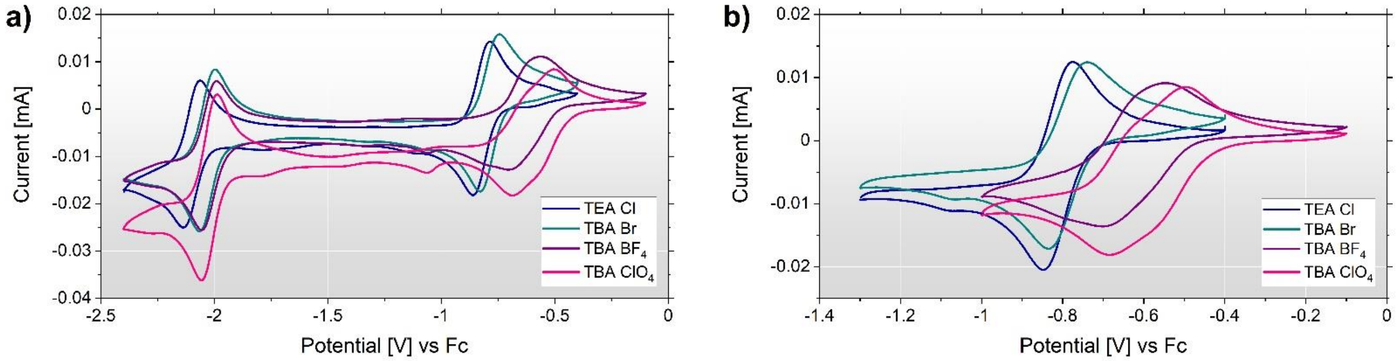

Figure S 14. Cyclic voltammetry of $Fe(mnt)_2^{1-}$ as a function of electrolyte composition under N_2 at a GC electrode. The electrolytes were composed of 1 mM (TEA)[$Fe(mnt)_2$] with 0.1 M supporting electrolyte in MeCN. Second scans shown at 100 mV s^{-1}. a) wide potential range including both the $Fe(mnt)_2^{1-/2-}$ and $Fe(mnt)_2^{2-/3-}$ redox couples. b) narrow potential range showing the $Fe(mnt)_2^{1-/2-}$ redox couple only.

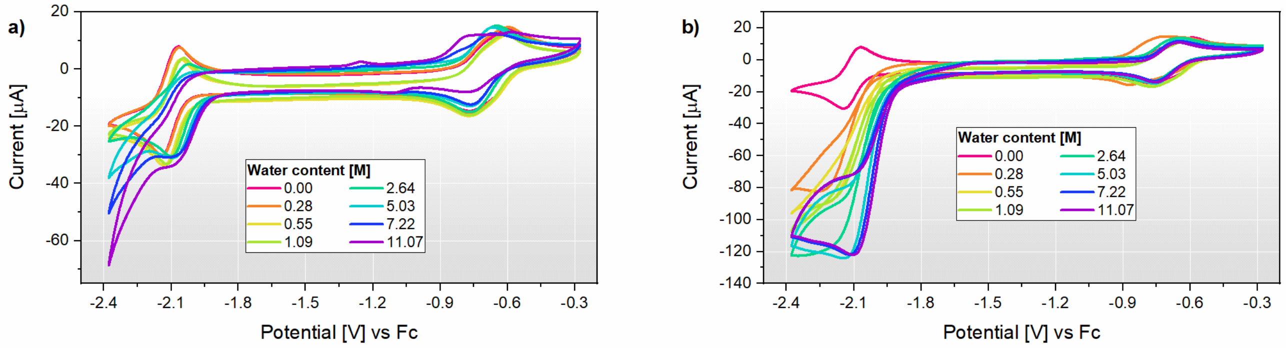

Figure S 15. Cyclic voltammetry of $Fe(mnt)_2^{1-}$ as a function of water content at a GC electrode under a) N_2 and b) CO_2. Second scans shown at 100 mV s^{-1}. Water was added sequentially by use of a volumetric pipettor. The calculated concentrations of $Fe(mnt)_2^{1-}$ and $TBAPF_6$ are given in Table S 9.

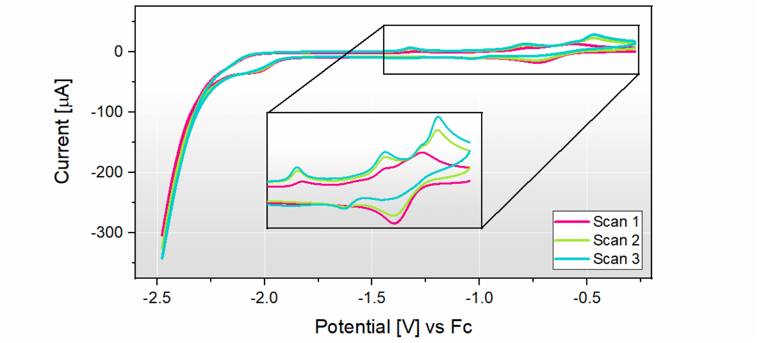

Figure S 16. Cyclic voltammetry of $Fe(mnt)_2$ as a function of scan number at a GC electrode under N_2. The electrolyte was composed of 1 mM (TEA)[$Fe(mnt)_2$] with 7 M H_2O and 100 mM $TBAPF_6$ in MeCN. A magnified view of the boxed region is given in the inset graph.

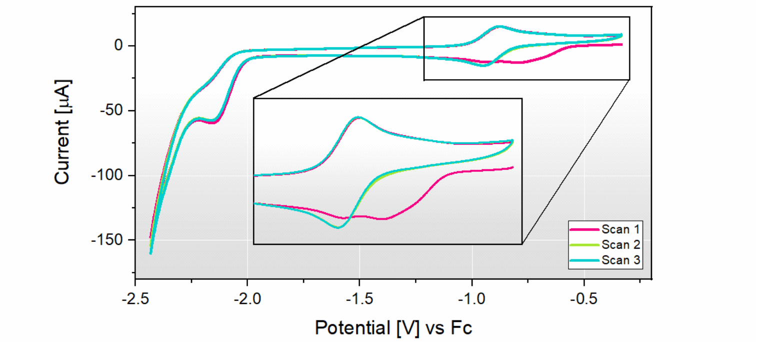

Figure S 17. Cyclic voltammetry of $Fe(mnt)_2$ as a function of scan number at a GC electrode under N_2. The electrolyte was composed of 1 mM (TEA)[$Fe(mnt)_2$] with 0.5 M TFE and 100 mM $TBAPF_6$ in MeCN. A magnified view of the boxed region is given in the inset graph.

Table S 9. Calculated water, $Fe(mnt)^{1-}$ and $TBAPF_6$ concentrations within the electrolytes used in Figure S 13. Dilution of the electrolyte is due to sequential addition of water to the anhydrous electrolyte by use of a volumetric pipettor.

Water concentration [M]	**$Fe(mnt)^{1-}$ concentration [mM]**	**$TBAPF_6$ concentration [M]**
0.00	1.000	0.100
0.28	0.995	0.100
0.55	0.990	0.099
1.09	0.980	0.098
2.64	0.952	0.095
5.03	0.909	0.091
7.22	0.870	0.087
11.07	0.800	0.080

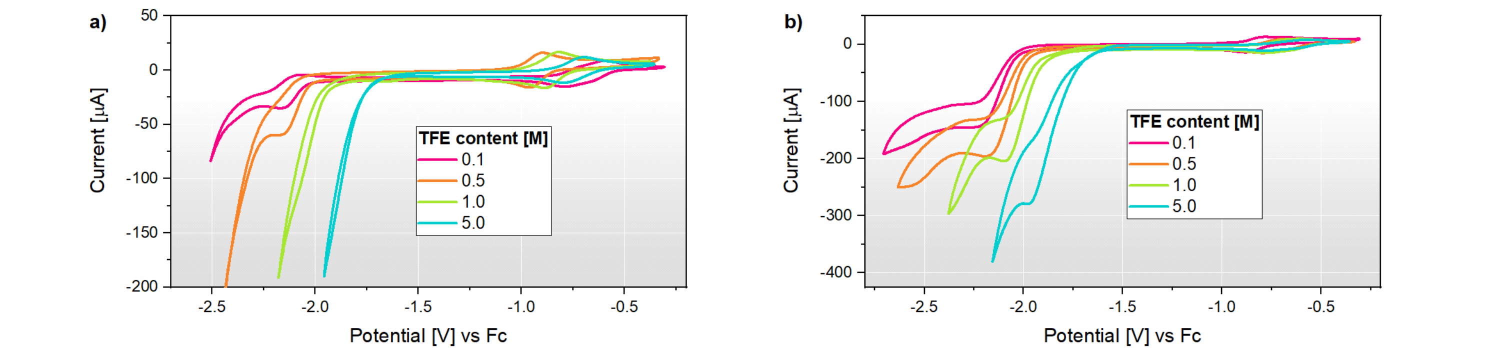

Figure S 18. Cyclic voltammetry of $Fe(mnt)_2^{1-}$ as a function of 2,2,2-triflouroethanol content at a GC electrode under a) N_2 and b) CO_2. Second scans shown at 100 mV s^{-1}. Each solution consisted of 1 mM $Fe(mnt)_2^{1-}$ with 100 mM $TBAPF_6$ in MeCN.

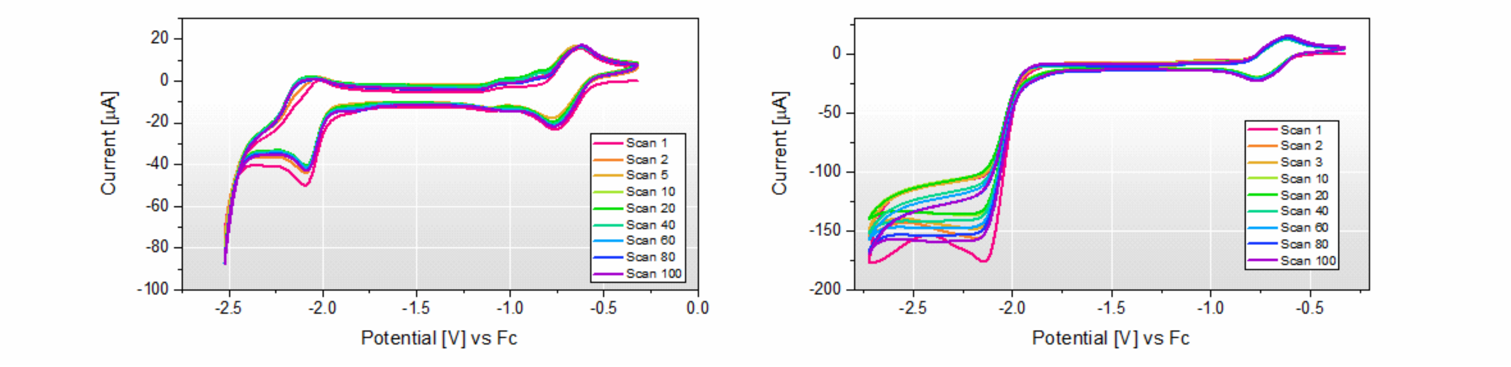

Figure S 19. 100 voltammetry scans of the $Fe(mnt)_2$ electrochemical response towards a) HER under N_2 and b) CO_2R under CO_2. The electrolyte was composed of 1 mM $(TEA)[Fe(mnt)_2]$ with 3 M H_2O and 100 mM $TBAPF_6$ supporting electrolyte in MeCN solvent. Scans recorded at 100 mV s^{-1} at a GC electrode. The total experiment times were 1 h 13 min and 1 h 20 min respectively for a) and b).

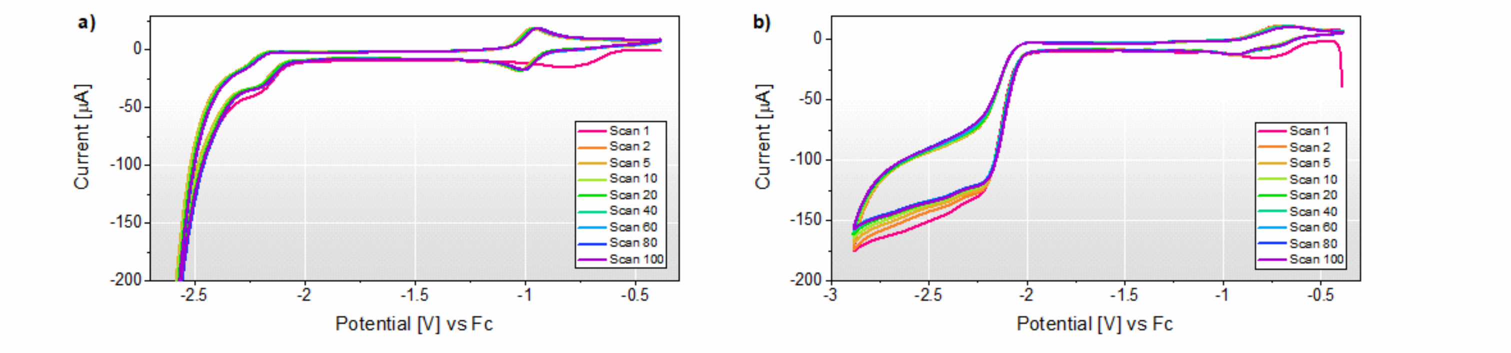

Figure S 20. 100 voltammetry scans of the $Fe(mnt)_2$ electrochemical response towards a) HER under N_2 and b) CO_2R under CO_2. The electrolyte was composed of 1 mM $(TEA)[Fe(mnt)_2]$ with 100 mM TFE and 100 mM $TBAPF_6$ supporting electrolyte in MeCN solvent. Scans recorded at 100 mV s^{-1} at a GC electrode. The total experiment times were 1h13min and 1h23min respectively for a) and b).

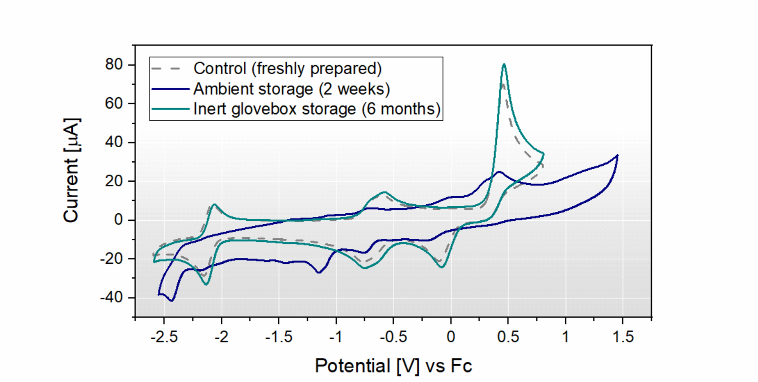

Figure S 21. Cyclic voltammetry of aged catalyst solutions stored under different conditions. The electrolytes were composed of 1 mM (TEA)[Fe(mnt)$_2$] with 100 mM TBAPF$_6$ in MeCN solvent. Scans recorded at 100 mV s^{-1} at a GC electrode. The solutions were prepared with identical chemicals before storage under inert and ambient conditions.

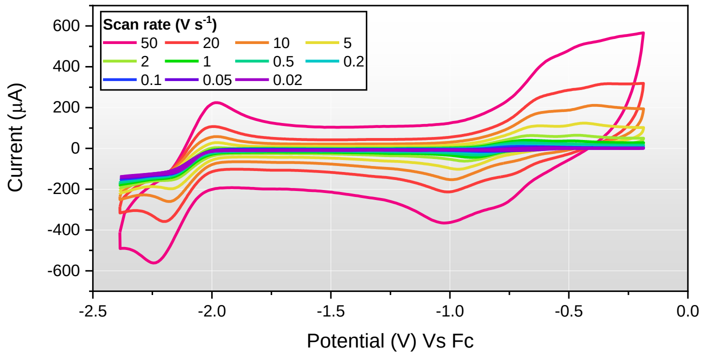

Figure S 22. Scan rate dependence of Fe(mnt)$_2$ at a GC electrode under CO_2 with TFE proton source. The electrolyte was composed of 1 mM (TEA)[Fe(mnt)$_2$] with 0.1 M TFE and 100 mM TBAPF$_6$ in MeCN. Scan rate was varied between 50 and 0.02 V s^{-1}.

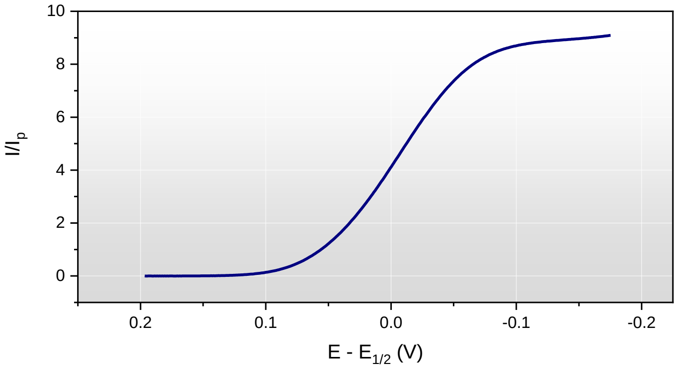

Figure S 23. Plot of the diffusion normalised peak current I/I_p vs $E - E_{1/2}$ calculated from the reduction wave of a cyclic voltammogram of CO_2 saturated 1 mM (TEA)[Fe(mnt)$_2$] with 0.1 M TFE and 100 mM TBAPF$_6$ in MeCN taken at 0.1 V s^{-1}, where I_p is the diffusion limited peak current of a one-electron reduction wave of the same complex at the same scan rate under reversible conditions.

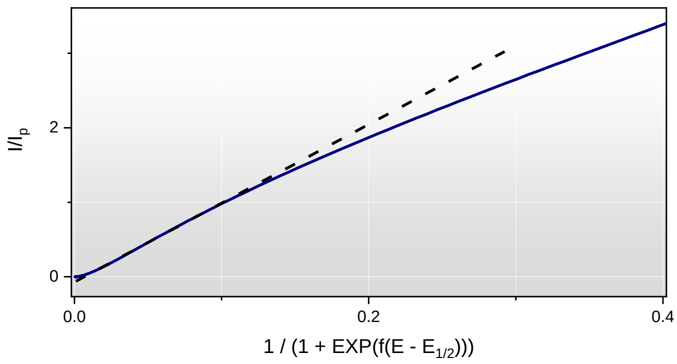

Figure S 24. Plot of the diffusion normalised peak current I/I_p vs 1 / (1 + EXP[f(E – $E_{1/2}$)]) for the system described in Figure S 21 for use in front of wave analysis. The gradient of the initial linear portion (dashed black line) can be used to determine the TOF_{max} using equation 1 as discussed in the main text.

5 UV/vis Spectra

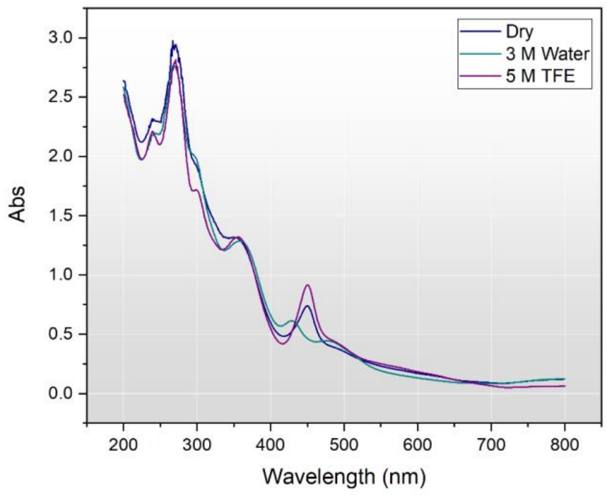

Figure S 25. Room temperature UV/Vis spectra of 1 mM (TEA)[Fe(mnt)$_2$] with 100 mM TBAPF$_6$ in MeCN and no proton source (blue), 3 M water (cyan) or 5 M TFE (purple).

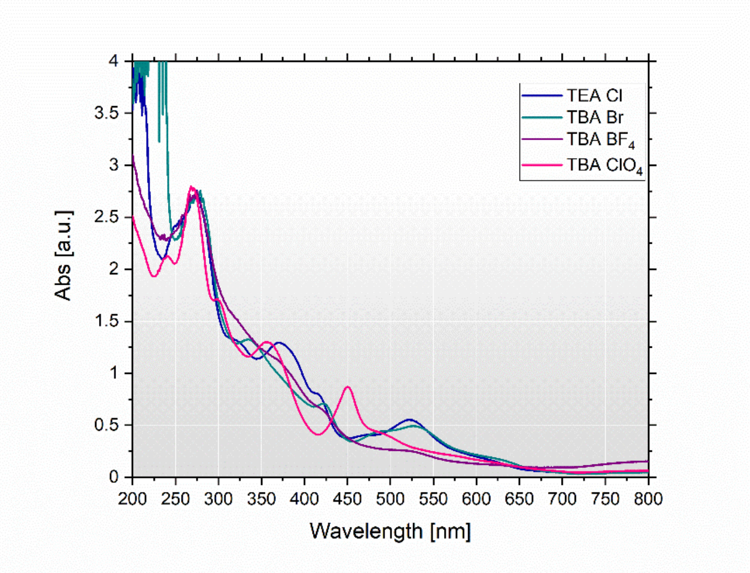

Figure S 26. Room temperature UV/Vis spectra of 1 mM (TEA)[Fe(mnt)$_2$] in varying supporting electrolyte at 100 mM concentration in MeCN. TEACl (Navy), TBABr (Cyan), TBABF$_4$ (Purple) and TBAClO$_4$ (Pink).

6 Glass bulk electrolysis cell design

A custom glass electrochemical cell was designed for conducting bulk electrolysis experiments with the homogeneous catalyst. The whole electrochemical cell is comprised of two glass half-cells which are combined to create a traditional glass H-cell, whereby a membrane separates each half-cell. Each half-cell design was first created using conventional 3D modelling software (Autodesk Fusion 360), as shown in Figure S 28, and then prototyped using a 3D printer (Anycubic Photon laser

3D printer). Glass equivalents of the design were then manufactured via glass-blowing techniques by Scientific Glass Laboratories Ltd. To combine the two glass half-cells, a glass-flange ‘knuckle clamp’ and a polytetrafluoroethylene (PTFE) spacer (EVAC AG, Switzerland) were employed. To ensure a pressure-tight and leak-proof connection, additional gaskets composed of ethylene propylene diene monomer (EPDM) rubber were used to tightly hold the Nafion® 117 membrane. The fully-assembled glass cell is shown in Figure S 27.

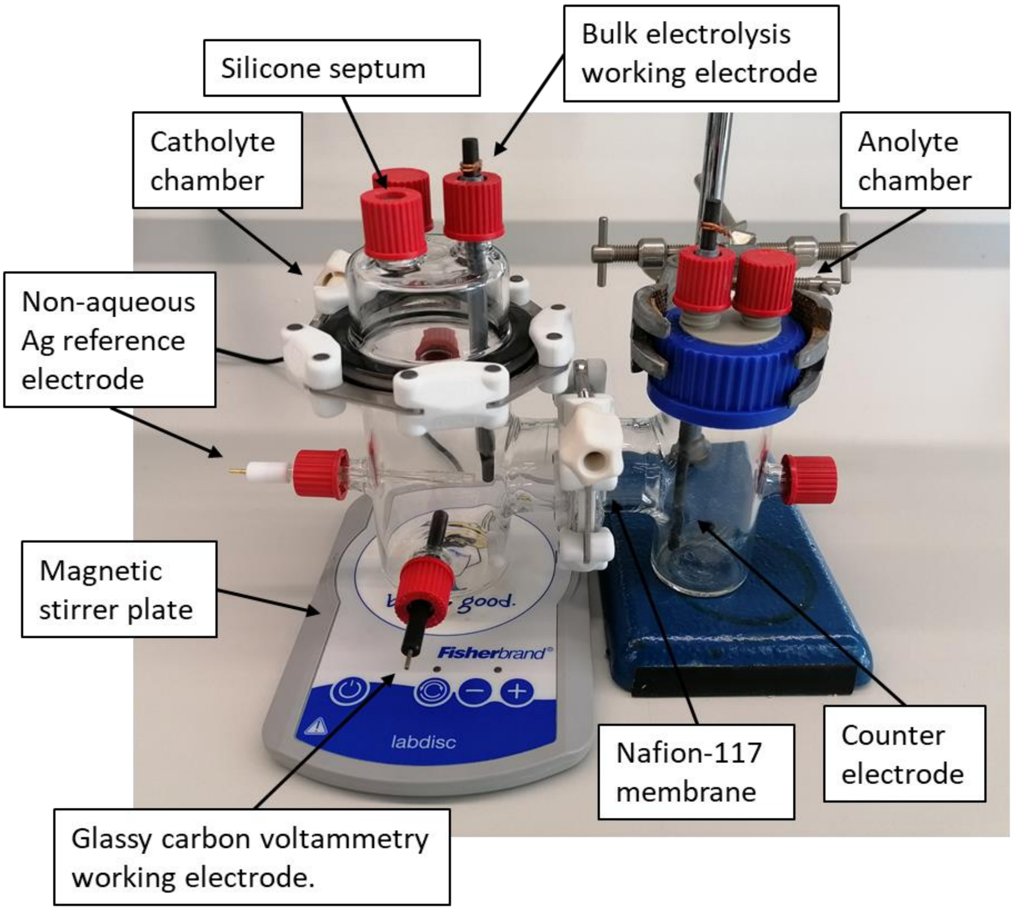

Figure S 27. Photograph of the bulk electrolysis glass cell used for product analysis studies.

Standard plastic threaded fittings (sizes GL 14 and GL 45, sourced from Duran®) and pipe fittings (Cole Parmer) were employed for gas-tight electrode and tubing access. For gas phase analysis, a silicone septum was attached to the top of the catholyte half-cell. In each electrolyte chamber a graphite rod electrode (Goodfellow, 99.997 % purity) was inserted to serve as the working and counter electrode in the catalyst and sacrificial electrolytes respectively. Within the catholyte a non-aqueous silver quasi-reference electrode (Ag wire submerged in supporting electrolyte, separated from the catholyte by an ion-conductive glass frit) was positioned within 2 mm of the working electrode. A glassy carbon working electrode (3 mm diameter) was also introduced into the electrolyte for conducting voltammetry experiments. A magnetic stirrer bar was placed within the catholyte below the electrodes and stirred continuously during electrolysis. In contrast, nitrogen or argon gas was continuously bubbled through the sacrificial electrolyte to provide convection. In addition, the inert gas was also employed to protect the electrochemically generated ferrocenium ion from undesirable decomposition via oxygen reactivity.[6]

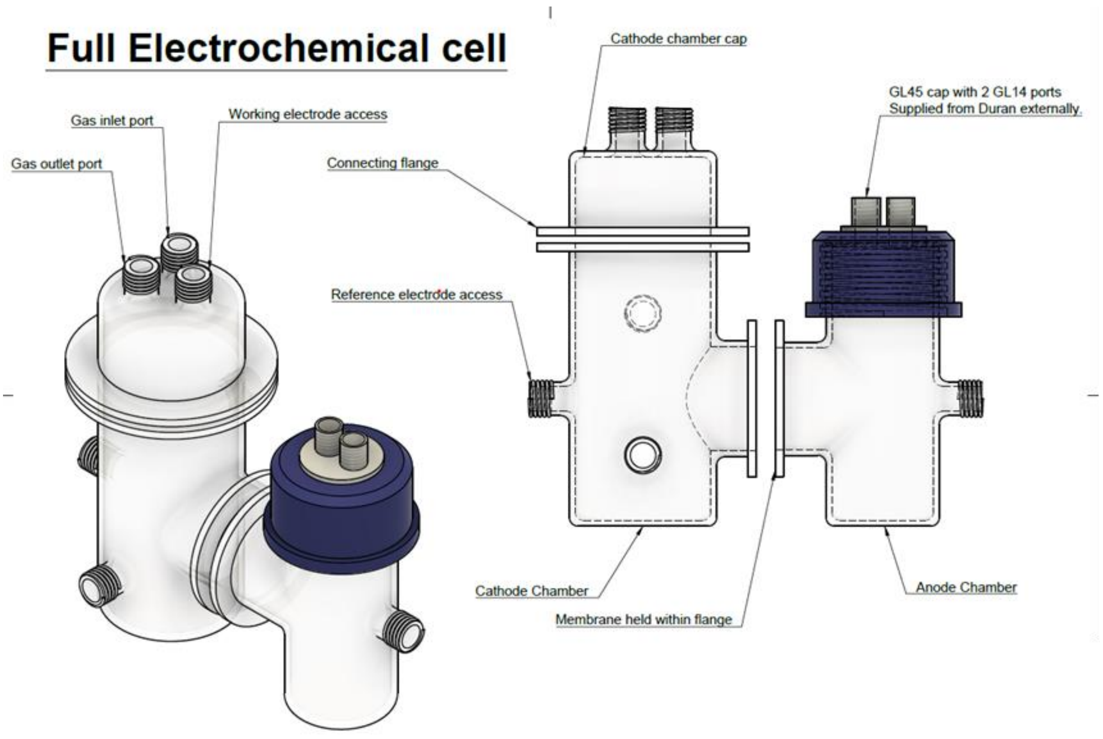
Full Electrochemical cell
Gas inlet port
Working electrode access
Gas outlet port
Connecting flange
Reference electrode access
Cathode chamber cap
GL45 cap with 2 GL14 ports
Supplied from Duran externally.
Cathode Chamber
Membrane held within flange
Anode Chamber

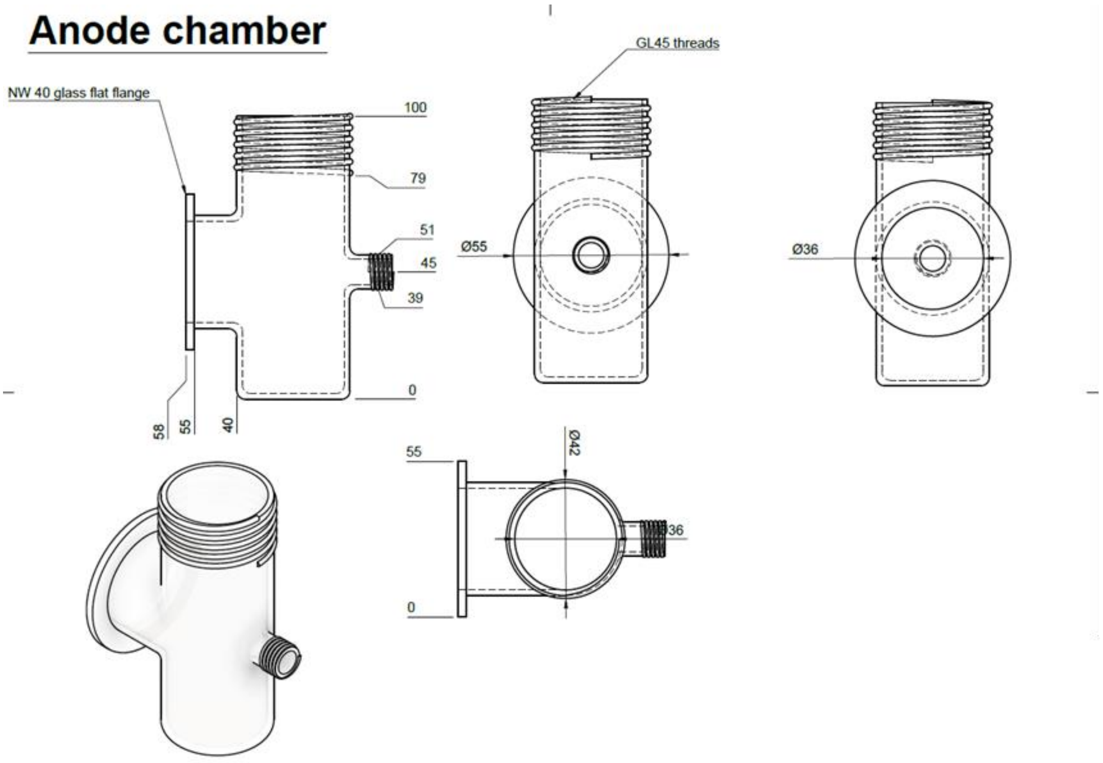
Anode chamber
NW 40 glass flat flange
GL45 threads
100
79
51
45
39
0
58
55
40
Ø55
Ø36
Ø42
55
0
36

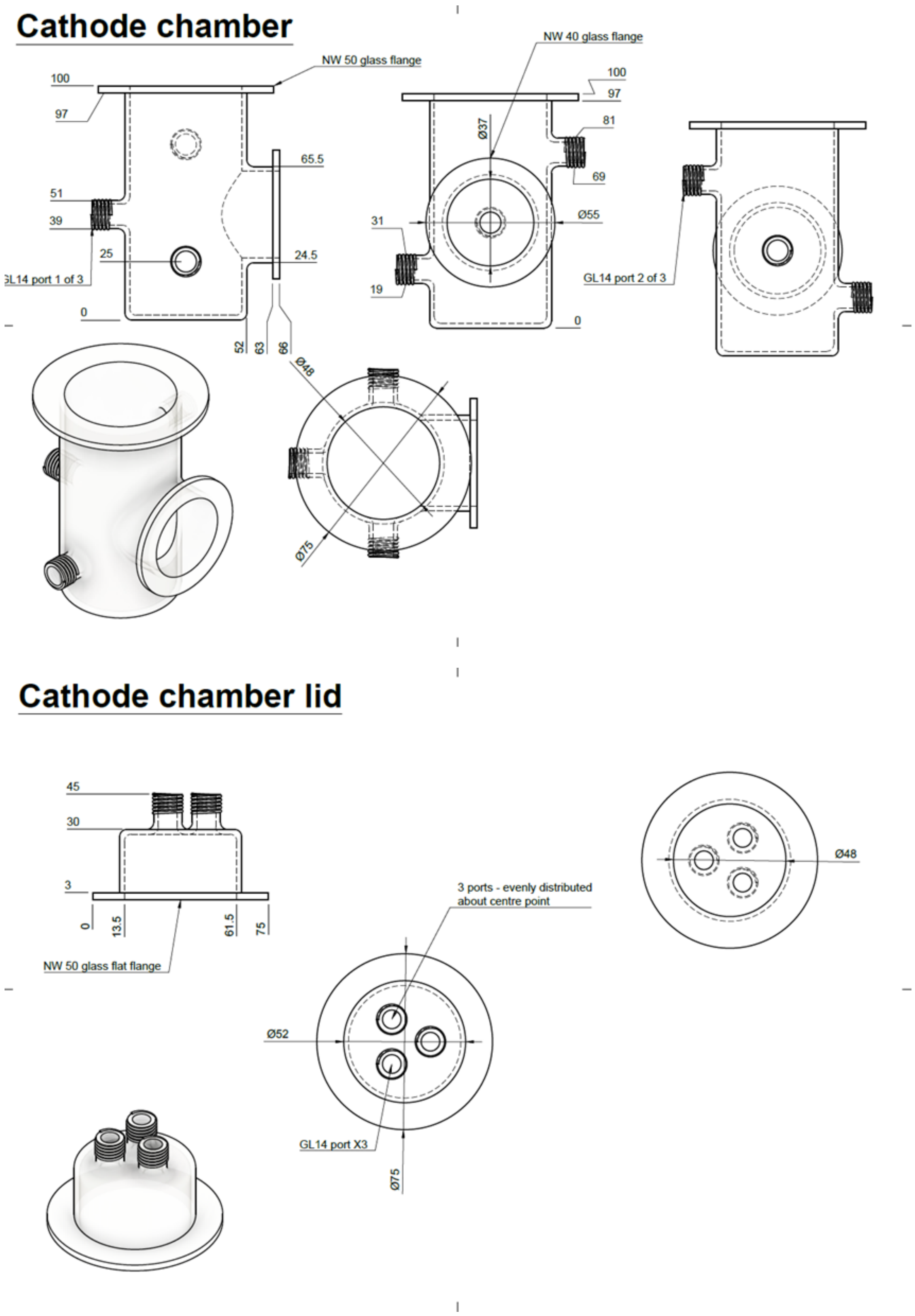

Figure S 28. Schematic drawings of the glass bulk electrolysis cell components. Measurements are given in units of millimetres.

7 Gas Chromatography calibrations

Before electrolysis experiments were performed, the gas chromatograph (GC), equipped with a barrier-ionisation detector, was calibrated by use calibration gas supplied by BOC Ltd. The gas was comprised of 976 ppm H_2, 1009 ppm CH_4, 1040 ppm CO and 1018 ppm C_2H_4, with He to balance. Dilution of the calibration gas was performed by use of a pressure vessel assembled out of stainless steel fittings (Swagelok Ltd.), shown in Figure S 29. The vessel was equipped with a 150 mL volume gas storage bottle, two valve inlets and a positive pressure gauge. The total internal volume of the vessel was measured to be 160 mL and the operating pressure range was 0 to 3 bar.

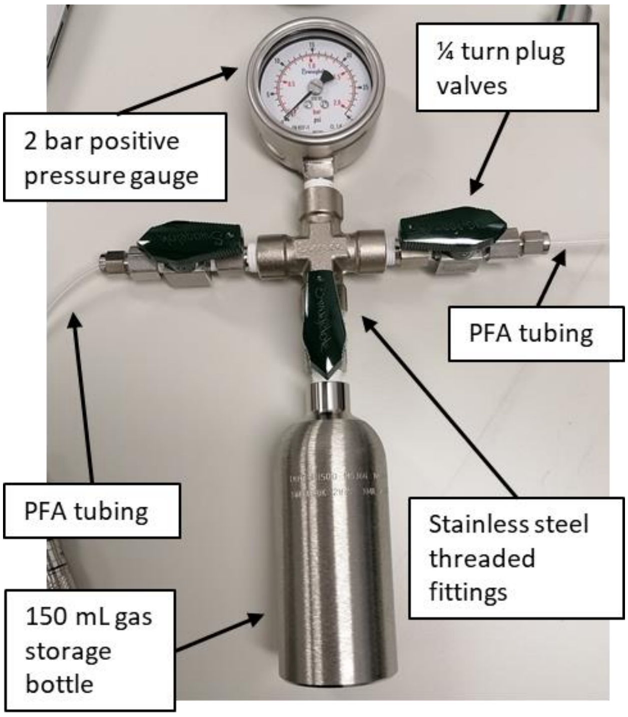

Figure S 29. Schematic diagram of the gas pressure vessel employed for the collection and analysis of the electrolysis cell headspace. The flexible plastic tubing is composed of perfluoroalkoxy alkane polymer (PFA).

Dilutions were performed by filling the pressure vessel with additional CO_2 and recording the vessel pressure. Series dilutions were prepared by venting the vessel to atmospheric pressure and refilling with CO_2 in repetition as needed. The diluted concentration of each sample were then calculated by use of;

$$[\mathrm{X}]_{\mathrm{diluted}} = [\mathrm{X}]_{\mathrm{undiluted}} \times \frac{\mathrm{P}_{\mathrm{initial}}}{\mathrm{P}_{\mathrm{final}}}$$

where [X] is the concentration of a component in ppm and P is the pressure of the sample. Injection of the vessel sample was performed by venting the vessel pressure into the GC inlet after flushing the GC sample loop with CO_2 (Figure S 30). To ensure reliable and reproducible sample injection into the GC, the vessel pressure was at minimum +0.8 bar which corresponds to >100 mL of gas flushed through the GC loop. This ensured saturation of the GC manifold and sample loop with a representative electrolysis sample.

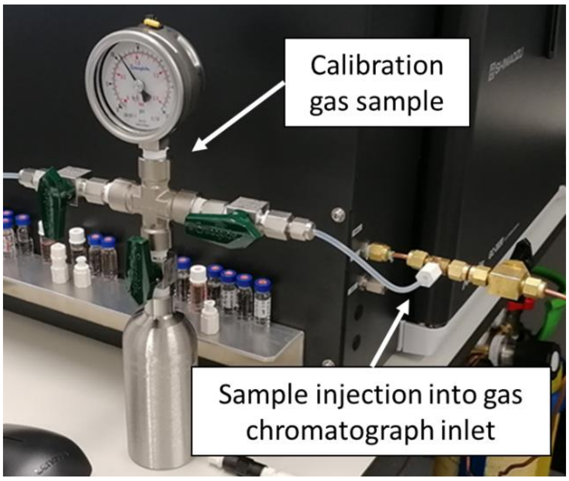

Figure S 30. Schematic diagram illustrating the connection of the gas pressure vessel to the gas chromatograph for calibration.

Calibration samples were prepared in the range of ~15 to 1000 ppm for each gas component and each sample was prepared and analysed in triplicate. The corresponding 12 point calibrations for each gas component are given in Figure S 32**Error! Reference source not found.** to Figure S 35. y-axis errors were calculated from the standard deviation in the mean peak integration (across the three runs) for each dilution. The corresponding X-axis errors were calculated from the uncertainty in the analogue pressure gauge readings. This uncertainty was estimated to be ±0.02 bar given that the gauge has increments of 0.1 bar and intermediate values can be estimated to an approximate accuracy of 0.2 of each division. Note that in all samples, the vessel was filled to marked increments on the gauge, for example +1.2 bar. The error in the sample concentration was then calculated by use of standard error propagation rules. X and Y errors were used to fit the data with linear relationships relating the GC-BID peak integration to the sample concentration (Figure S 31). In each case significant non-linearity in the data was observed which originated from the BID detector. Therefore, each dataset was fit to give low (0 to 110 ppm) and high (110 to 1000 ppm) concentration linear regions.

Limits of detection and quantification (LOD and LOQ respectively) were calculated from the GC-BID calibrations by use of the following relations

$$\mathrm{LOD} = 3.3\,\frac{\sigma_{\mathrm{intercept}}}{|\mathrm{gradient}|}$$

$$\mathrm{LOQ} = 10\,\frac{\sigma_{\mathrm{intercept}}}{|\mathrm{gradient}|}$$

where $|\mathrm{gradient}|$ is the magnitude of the gradient and $\sigma_{\mathrm{intercept}}$ is the standard deviation in the intercept. In turn this is related to the standard error of the intercept (SE) and N, which is the number of replicates in the dataset (3 in this work).

$$\sigma_{\mathrm{intercept}} = SE_{intercept} \times \sqrt{N}$$

Corresponding LOD and LOQ values are given in Table S 10. In all samples analysed, the analyte concentrations significantly exceeded the LOD and were typically no less than 50 ppm.

Table S 10. Calculated limits of detection and quantification in the GC-BID calibration.

Analyte	LOD [ppm]	LOQ [ppm]
H_2	8.5	25.9
CO	11.3	34.2
CH_4	12.7	38.4
C_2H_4	11.7	35.6

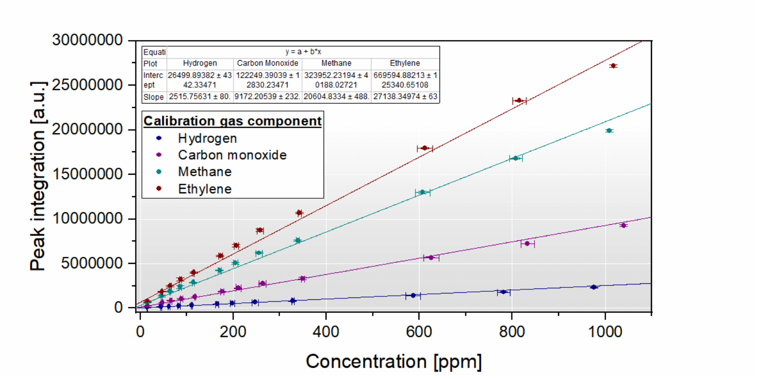

Figure S 31. GC-BID calibration curves for H_2, CO, CH_4 and C_2H_4 analytes.

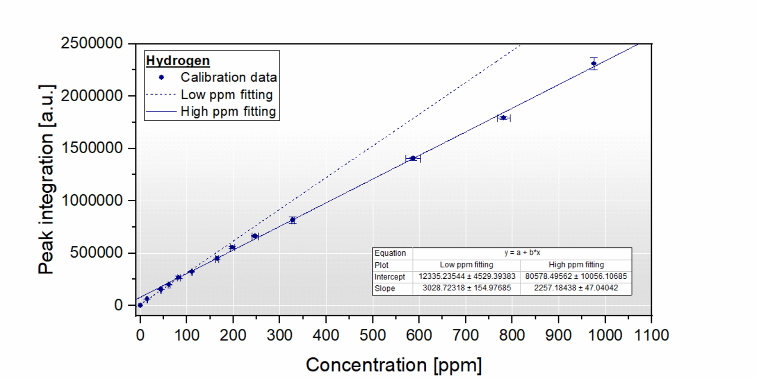

Figure S 32. GC-BID calibration curve for H_2 gas.

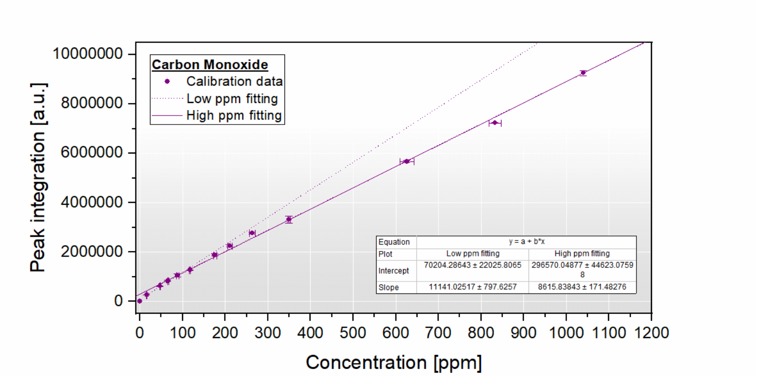

Figure S 33. GC-BID calibration curve for CO gas.

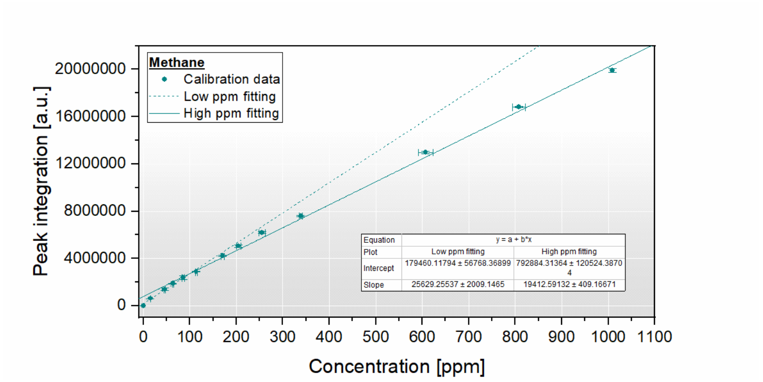

Figure S 34. GC-BID calibration curve for CH_4 gas.

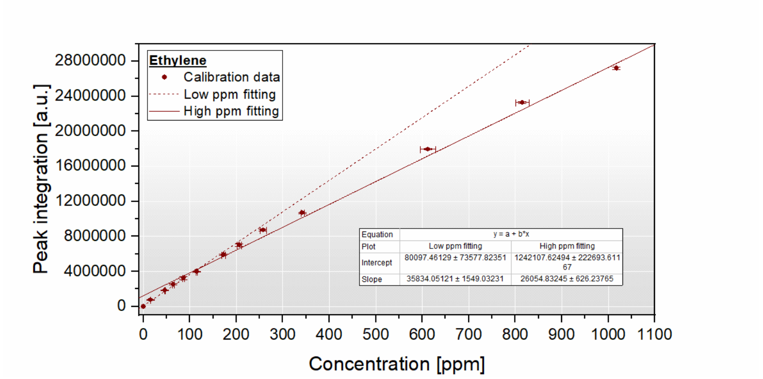

Figure S 35. GC-BID calibration curve for C_2H_4 gas.

8 Bulk electrolysis

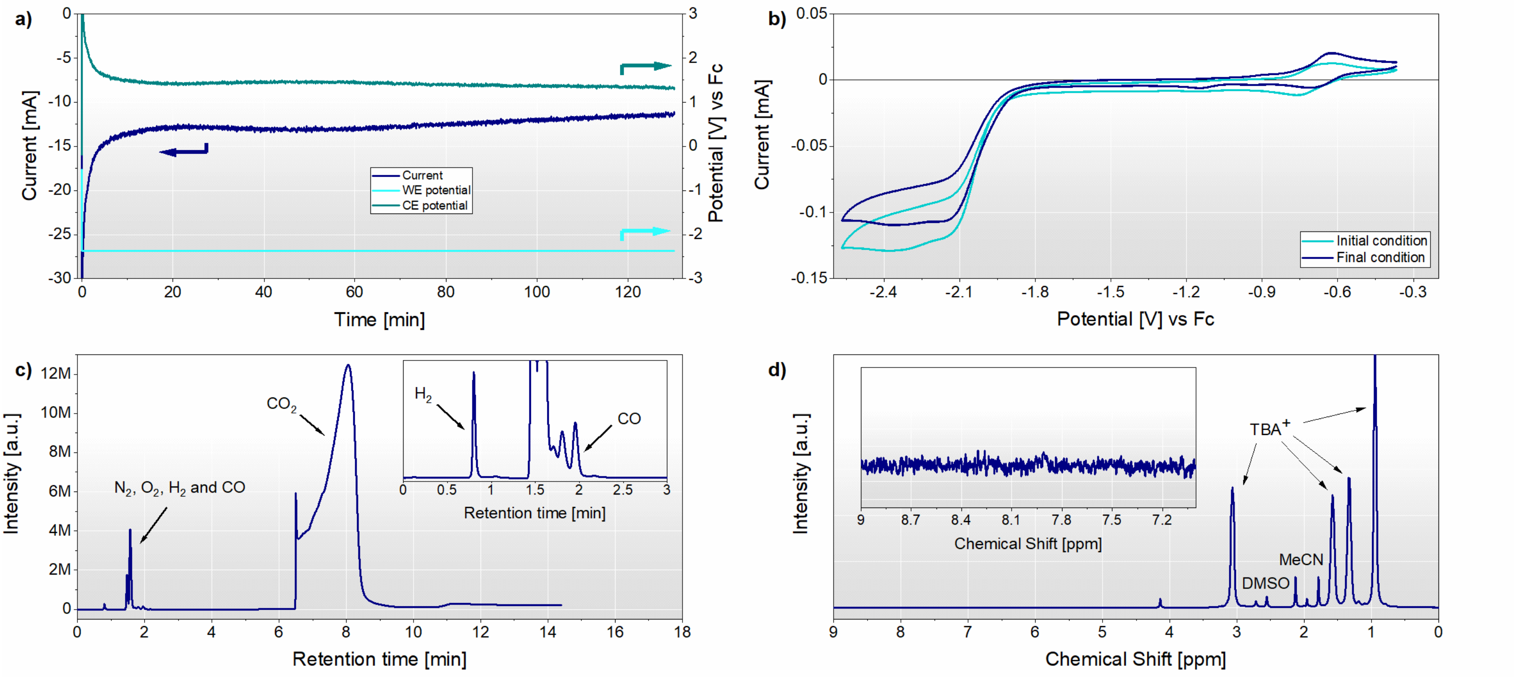

Figure S 36. Representative CO_2R bulk electrolysis and analysis of 1 mM $Fe(mnt)_2$ with 3 M H_2O proton source and 100 mM $TBAPF_6$ supporting electrolyte in MeCN solvent. a) Electrolysis current response as a function of time at -2.36 V vs Fc. b) CV response of the catalyst under CO_2 immediately before and after electrolysis at 100 mV s^{-1}. c) GC analysis of the cell headspace after a hundredfold sample dilution. The inset graph highlights peak features before three minute retention time. d) NMR analysis of the post-electrolysis catalyst solution with 10 mM DMSO internal standard. The inset graph highlights the lack of formic acid peak.

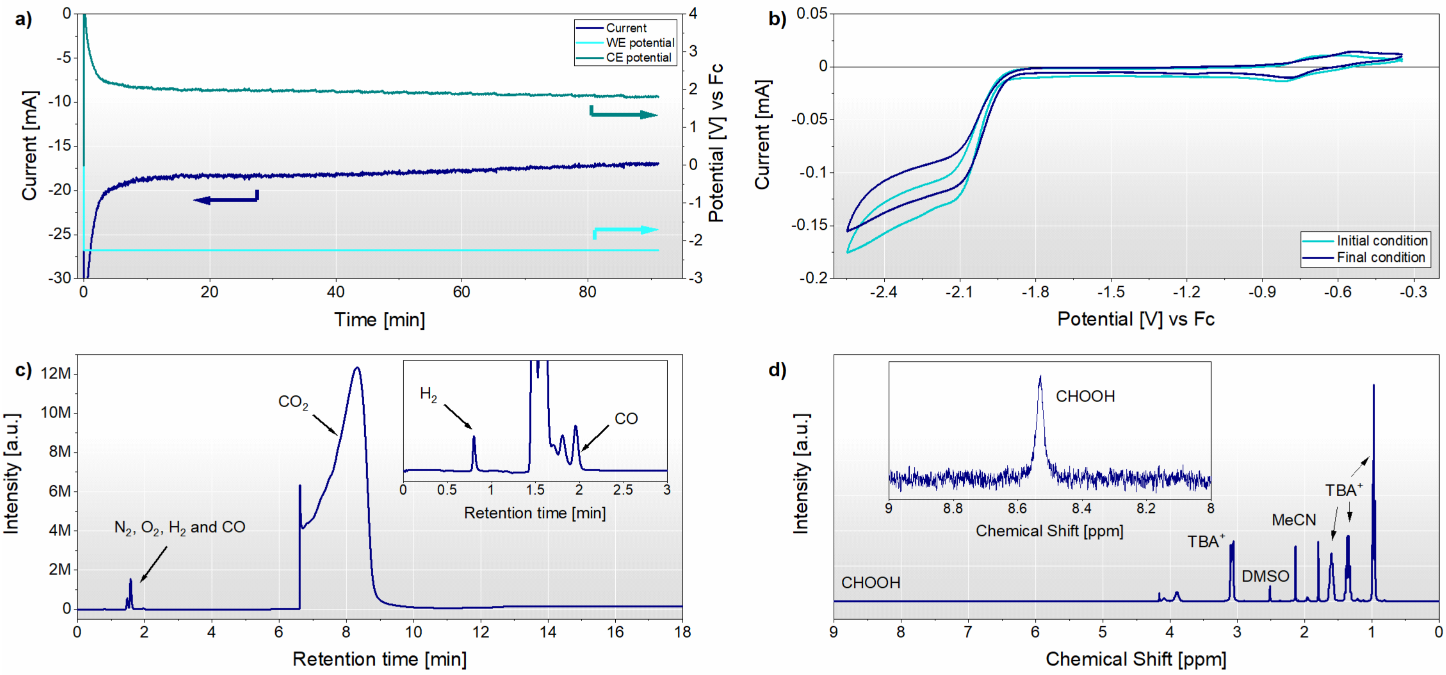

Figure S 37. Representative CO_2R bulk electrolysis and analysis of 1 mM $Fe(mnt)_2$ with 100 mM TFE proton source and 100 mM $TBAPF_6$ supporting electrolyte in MeCN solvent. a) Electrolysis current response as a function of time at -2.25 V vs Fc. b) CV response of the catalyst under CO_2 immediately before and after electrolysis at 100 mV s^{-1}. c) GC analysis of the cell headspace after a tenfold sample dilution. The inset graph highlights peak features before three minute retention time. d) NMR analysis of the post-electrolysis catalyst solution with 10 mM DMSO internal standard. The inset graph highlights the formic acid peak compared to the background signal.

9 Calculation of faradaic efficiencies

Gaseous products – H_2 and CO

After completion of a catalyst electrolysis experiment, the cell gaseous headspace was sampled by use of a 100 mL gas-tight syringe (VICI® precision sampling Ltd.). Gas samples were then diluted a hundredfold to dilute the sample analyte concentrations into the calibration range of the GC-BID. Dilutions were performed by firstly extracting a 10 mL sample of the cell headspace and then filling the syringe by addition of 90 mL of background gas (either CO_2 or Ar). The syringe was then emptied to 10 mL and refilled as before. The diluted sample was then directly injected into the GC-BID inlet and analysed. By use of the appropriate calibration fitting, the sample concentration of analytes (H_2 and CO) were then calculated to give concentrations in units of ppm. The undiluted concentration was then calculated by use of;

$$Headspace\ concentration\ [ppm] = Diluted\ concentration\ [ppm] \times 100$$

The total moles of electrolysis product (H_2 or CO) was then calculated by use of the numerical ratio and ideal gas equation;

$$Moles\ product\ [mol] = \frac{Headspace\ conentration\ [ppm]}{10^6} \times Moles\ of\ gas\ in\ headspace\ [mol]$$

where;

$$Total\ moles\ of\ gas\ in\ headspace = \frac{Headspace\ pressure\ [Pa] \times Headspace\ volume\ [m^3]}{Ideal\ gas\ constant\ [J\ K^{-1}\ mol^{-1}] \times Temperature\ [K]}$$

Here the headspace volume was accurately measured to be 120 mL whereas the pressure and temperature were assumed to be atmospheric (101325 Pa and 298.15 K), such that additional pressure produced by the CO_2R reaction is negligible.

The charge required to produce the quantity of detected product was then calculated by use of;

$$Charge\ required\ [C] = Moles\ product\ [mol] \times Stoichiometry \times Faraday\ constant\ [C\ mol^{-1}]$$

where the stoichiometry is the number of electrons per CO_2R product (2 electrons for H_2 and CO) and the Faraday constant is 96485.4 C mol^{-1}.

The faradaic efficiency was then calculated by use of;

$$Faradaic\ efficiency = \frac{Charge\ required\ [C]}{Electrolysis\ charge\ [C]} \times 100$$

Liquid product - CHOOH

After completion of a catalyst electrolysis experiment, the concentration of CHOOH in the catalyst solution was measured by H^1 NMR. To quantify the CHOOH concentration, the $HCOO^-$ singlet peak integration was compared to that of the DMSO singlet, added post-experiment as an internal standard. Each NMR sample was prepared by addition of 2 mL of 100 mM DMSO solution (in MeCN) to 18 mL of catalyst solution in a 20 mL volumetric flask, giving a 10 mM DMSO concentration. NMR spectra were then obtained using a solvent suppression method (dual solvent suppression used for samples containing water as the proton source). The DMSO peak integration was then measured relative to the CHOOH peak and normalised on a proton basis by use of;

$$Numerical\ ratio = \frac{Proton\ basis\ ratio}{6\ protons}$$

Thus, the CHOOH concentration was then calculated by use of;

$$[CHOO^-]_{NMR\ sample}\ [M] = \frac{[DMSO]_{NMR\ sample}\ [M]}{Numerical\ ratio}$$

and

$$[CHOO^-]_{Electrolyte}[M] = [CHOO^-]_{NMR\ sample}[M] \times Dilution\ ratio$$

where the dilution ratio is

$$Dilution\ ratio = \frac{NMR\ solution\ volume\ [mL]}{Electrolyte\ diluted\ volume\ [mL]} = \frac{20\ mL}{18\ mL} = \frac{10}{9}$$

The moles of CHOOH produced is then calculated by use of;

$$Moles\ CHOO^-\ [mol] = [CHOO^-]_{Electrolyte}\ [M] \times Electrolyte\ volume\ [dm^3]$$

Correspondingly, the charge required to produce the formic acid is;

$$Charge\ required\ [C] = Moles\ CHOO^-\ [mol] \times Stoichiometry \times Faraday\ constant\ [C\ mol^{-1}]$$

where the stoichiometry is the number of electrons per CO_2R product (2 electrons for CHOOH) and the Faraday constant is 96485.4 C mol^{-1}.

The faradaic efficiency was then calculated by use of;

$$Faradaic\ efficiency = \frac{Charge\ required\ [C]}{Electrolysis\ charge\ [C]} \times 100$$

10 Catalyst instability

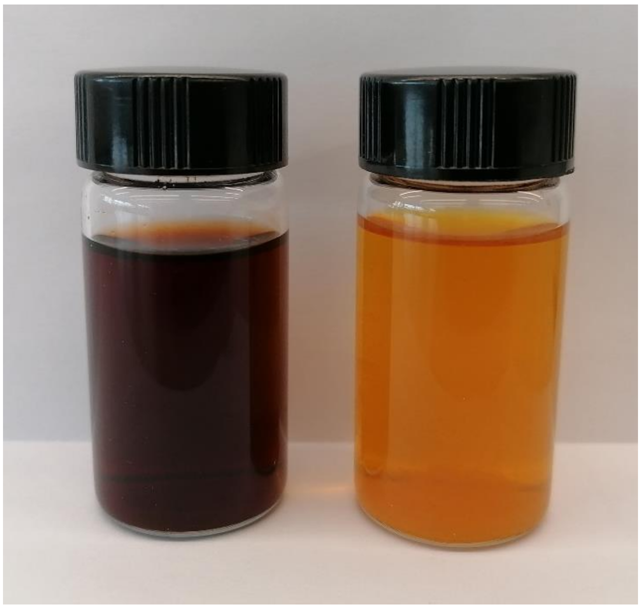

Figure S 38. A photograph comparing a freshly prepared $Fe(mnt)_2$ solution (left) to one that was stored for one week (right). A fine precipitate is visible in the bottom of the aged solution. The solution compositions were 1 mM catalyst with 100 mM $TBAPF_6$ and 3 M H_2O.

11 Density Functional Theory

Methodology

DFT simulations were performed using the Gaussian 09 program (Revision E.01)[7] with B3LYP exchange-correlation functional [8–12] and cc-pVDZ basis set.[13,14] Structures were optimised using an implicit solvent model (SMD),[15,16] for which acetonitrile (ε = 35.688) was chosen as the solvent, consistent with the experiment. Minima and transition state geometries were obtained as defined as points on the potential energy surface possessing solely positive curvature or those possessing negative curvature in a single direction, respectively. This was confirmed by computing harmonic vibrational frequencies such that each minimum possessed only real frequencies, whereas transition states possessed a single imaginary frequency reminiscent of the transition.

Initial geometries for the $Fe(mnt)_2$ catalyst were obtained from either the literature[17] or the experimental crystal structure. This geometry was then optimised with quartet, triplet and doublet multiplicities for the anionic, dianionic and trianionic oxidation states, respectively.[17] To account for the free energy of a solvated proton, the equilibria among the H_2O and TFE homoconjugates

$$\mathrm{H_2O} \rightleftharpoons \mathrm{OH^-} + \mathrm{H^+}$$

$$\mathrm{TFEH} \rightleftharpoons \mathrm{TFE^-} + \mathrm{H^+}$$

were calculated at the same level of theory and added explicitly to reaction states. To stabilise the electronic structure of the trianionic structures, a counterion was explicitly included in calculations in the form of tetraethylammonium (TEA^+). The energy of this counterion was then compensated in mechanism pathways by addition of the TEA^+ free energy to other reaction states. Associated free energy changes and barriers were computed relative to the initial reactants.

Transition states for the binding of H^+ and CO_2 to $Fe(mnt)_2^{3-}$ could not be obtained due to the relative flatness of the associated potential energy surface. As an alternative approximation of these states, scan-coordinate calculations were performed along the Fe-H and Fe-C bond lengths. From which, the associated energy of the structures with Fe-H bond length of 2.68 Å and Fe-C bond length of 3.16 Å were taken for energy analysis. Considering the transition state for the dissociation of $CHOO^-$ from $Fe(mnt)_2^{1-}$ a minor imaginary vibrational mode of 2 cm^{-1} was encountered that could not be removed. This mode was deemed negligible in using the obtained structure in energetic analysis. Considering the transition state for the dissociation of H_2 from $Fe(mnt)_2^{1-}$, a structure could not be obtained with cc-pVDZ basis set. Instead, a suitable structure was obtained using the aug-cc-pVTZ basis set and then the associated energy was calculated using the cc-pVDZ basis set.

Computation of equilibrium redox potential

Absolute reduction potentials were calculated by use of a Born-Haber cycle shown in Figure S 39. Here the cycle is constructed by firstly calculating minima geometries for both the oxidised and reduced form of the redox couple in both vacuum and with SMD implicit solvation. From which, the free energy changes of solvation are calculated in addition to the free energy of reduction in vacuum. The free energy of reduction in solution phase is then obtained by use of the equation;

$$\Delta G_{red\,(aq)} = \left(\Delta G_{red\,(g)} + \Delta G_{solv}\,[Fe(mnt)_2]^{2-}\right) - \Delta G_{solv}\,[Fe(mnt)_2]^{1-}$$

This method circumvents the need to calculate the energy of the solvated free electron through the neglect of the free electron energy in gas phase free-energy change and free energy of solvation. Note that at 0 K, the gas-phase free-energy change would typically correspond to electron affinity and ionisation potential which are analogues for reduction and oxidation potentials, respectively.

Once determined, the half-cell standard reduction potential can be calculated by use of the equation;

$$E^0_{abs} = \frac{-\Delta G_{red\,(aq)}}{nF} - 0.03766\ eV$$

where -0.03766 eV represents a free electron standard-state correction at 298 K, from 1 atm pressure to 1 M concentration.[18] Here F is the Faraday constant, whereas n is the electron stoichiometry. Computed absolute reduction potentials are reported as well as relative potentials referenced against the ferrocene/ferrocenium ion redox couple as in the experimental methodology. Here, the absolute reduction potential of the ferrocenium ion is computed at the same level of theory in the same manor described above, and then subtracted from the catalyst absolute reduction potential.

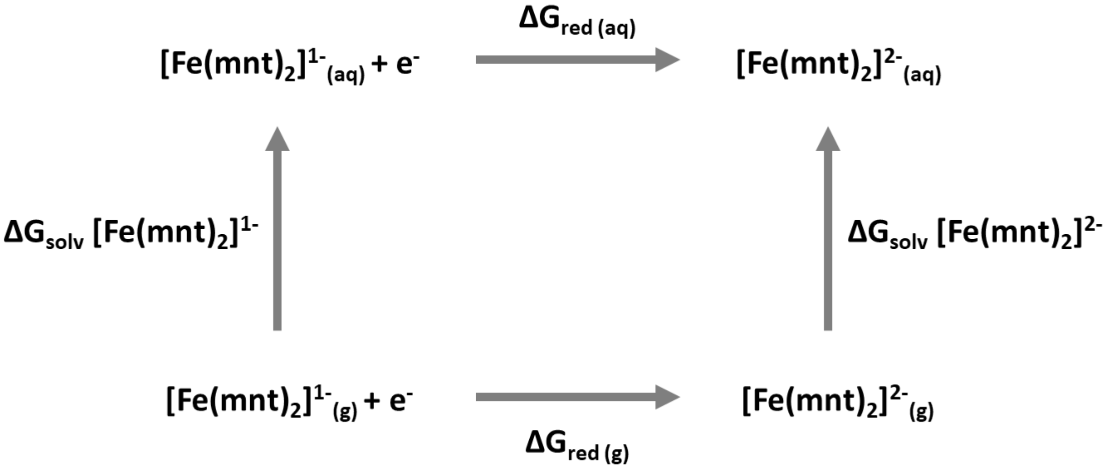

Figure S 39. Born-Haber cycle for the calculation of redox potential.

Absolute standard reduction potentials of 4.05 and 3.19 V were obtained for the ferrocene/ferrocenium ion and $[Fe(mnt)_2]^{1-/2-}$ redox couple, respectively. Correspondingly, the calculation of the $[Fe(mnt)_2]^{1-/2-}$ relative redox potential yielded a value of -0.87 V vs Fc which is consistent with the experimental value of -0.69 V vs Fc. Due to the difficulties in obtaining a realistic wavefunction for the trianionic $[Fe(mnt)_2]^{3-}$ oxidation state, and consequently the use of a charge-balancing TEA^+ counterion, a realistic $[Fe(mnt)_2]^{2-/3-}$ redox potential could not be obtained.

Reaction pathways

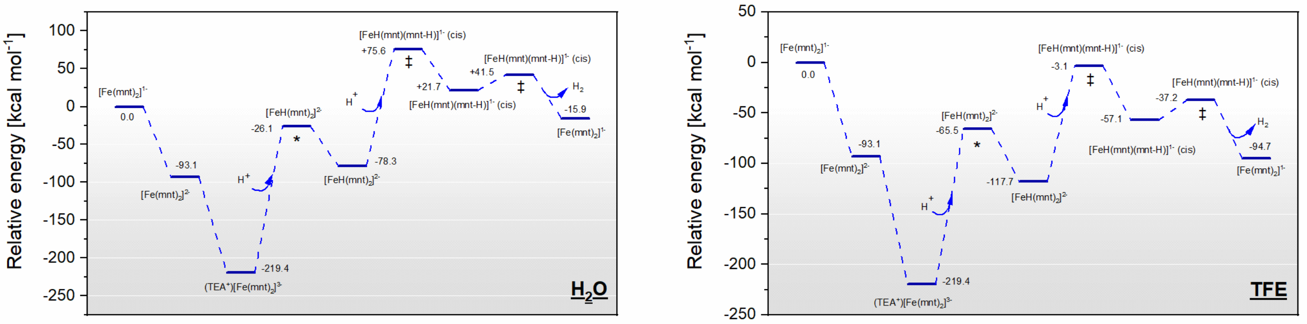

Figure S 40. Reaction pathway for the H_2 production mechanism with respect to H_2O and TFE proton source. Intermediate and transition state energies are given relative to the reactants and denoted on the y-axis and adjacent to states. Transition states are indicated by ‡ whereas * denotes the protonation transition state which was approximated by a Fe-H bond scan coordinate calculation, for which the structure with 2.68 Å bond length was used.

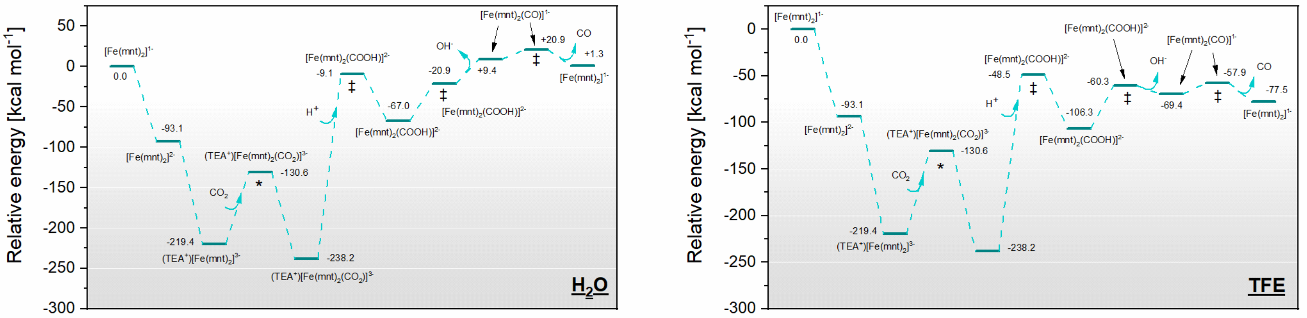

Figure S 41. Reaction pathway for the CO production mechanism with respect to H_2O and TFE proton source. Intermediate and transition state energies are given relative to the reactants and denoted on the y-axis and adjacent to states. Transition states are indicated by ‡ whereas * denotes the CO_2 binding transition state which was approximated by a Fe-C bond scan coordinate calculation, for which the structure with 3.16 Å bond length was used.

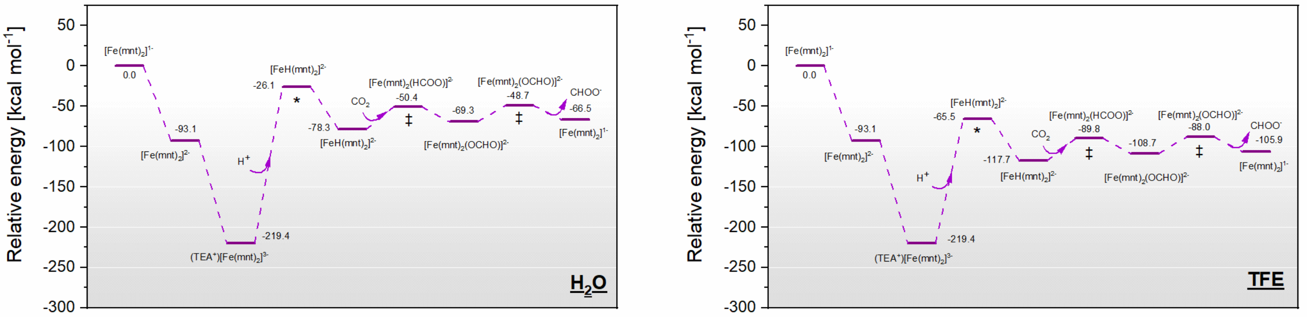

Figure S 42. Reaction pathway for the CHOOH production mechanism with respect to H_2O and TFE proton source. Intermediate and transition state energies are given relative to the reactants and denoted on the y-axis and adjacent to states. Transition states are indicated by ‡ whereas * denotes the protonation transition state which was approximated by a Fe-H bond scan coordinate calculation, for which the structure with 2.68 Å bond length was used.

Computed energies

Table S 11. Computed energies (the sum of electronic and thermal free energies) by use of B3LYP functional and cc-pvDZ basis set with SMD implicit solvent model (acetonitrile).

Structure	***Type***	**Free energy [kcal mol^{-1}]**
Catalyst oxidation states		
$[Fe(mnt)_2]^{1-}$	*intermediate – vacuum*	-2121352.563
$[Fe(mnt)_2]^{1-}$	*intermediate – SMD*	-2121388.84
$[Fe(mnt)_2]^{2-}$	*intermediate – vacuum*	-2121353.277
$[Fe(mnt)_2]^{2-}$	*intermediate – SMD*	-2121481.924
$(TEA^+)[Fe(mnt)_2]^{2-}$	*intermediate – vacuum*	-2354374.265
$(TEA^+)[Fe(mnt)_2]^{2-}$	*intermediate – SMD*	-2354443.909
$(TEA^+)[Fe(mnt)_2]^{3-}$	*intermediate – vacuum*	-2354329.107
$(TEA^+)[Fe(mnt)_2]^{3-}$	*intermediate – SMD*	-2354576.551
H_2 mechanism		
$[FeH(mnt)_2]^{2-}$	*Fe-H bond scan (2.68 Å length) – SMD*	-2121775.622
$[FeH(mnt)_2]^{2-}$	*intermediate – SMD*	-2121827.795
$[Fe(mnt)(mnt\text{-}H)]^{2-}$	*protonation – transition state – SMD*	not obtained
$[Fe(mnt)(mnt\text{-}H)]^{2-}$	*intermediate – SMD*	-2121813.071
$[Fe(mnt)(mnt\text{-}H)]^{2-}$	*H rearrangement – transition state – SMD*	-2121810.326
$[FeH(mnt)(mnt\text{-}H)]^{1-}$	*sulfur (cis) protonation – transition state – SMD*	-2122034.475
$[FeH(mnt)(mnt\text{-}H)]^{1-}$	*(cis) intermediate – SMD*	-2122088.423
$[FeH(mnt)(mnt\text{-}H)]^{1-}$	*sulfur (trans) protonation – transition state – SMD*	-2122040.727
$[FeH(mnt)(mnt\text{-}H)]^{1-}$	*(trans) intermediate – SMD*	-2122088.517
$[FeH(mnt)(mnt\text{-}H)]^{1-}$	*H-S-Fe-H torsion scan cis-trans barrier – SMD*	-2122068.086
$[FeH(mnt)(mnt\text{-}H)]^{1-}$	*H_2 dissociation – transition state – SMD*	-2122068.58
CO mechanism		
$(TEA^+)[Fe(mnt)_2(\eta^1\text{-}CO_2)]^{3-}$	*Fe-C bond scan (3.16 Å length) – SMD*	-2472838.931
$(TEA^+)[Fe(mnt)_2(\eta^1\text{-}CO_2)]^{3-}$	*intermediate – SMD*	-2472946.518
$(TEA^+)[Fe(mnt)_2(\eta^1\text{-}OCO)]^{3-}$	*CO_2 binding – transition state – SMD*	not obtained
$(TEA^+)[Fe(mnt)_2(\eta^1\text{-}OCO)]^{3-}$	*intermediate – SMD*	not obtained
$(TEA^+)[Fe(mnt)_2(\eta^2\text{-}CO_2)]^{3-}$	*CO_2 binding – transition state – SMD*	not obtained
$(TEA^+)[Fe(mnt)_2(\eta^2\text{-}CO_2)]^{3-}$	*intermediate – SMD*	not obtained
$[Fe(mnt)_2(C(O)OH)]^{2-}$	*protonation – transition state – SMD*	-2240109.813
$[Fe(mnt)_2(C(O)OH)]^{2-}$	*intermediate – SMD*	-2240167.647
$[Fe(mnt)_2(C(O)OH)]^{2-}$	*OH^- dissociation – transition state – SMD*	-2240121.587
$[Fe(mnt)_2(CO)]^{1-}$	*intermediate – SMD*	-2192495.639
$[Fe(mnt)_2(CO)]^{1-}$	*CO dissociation – transition state – SMD*	-2192484.169
CHOOH mechanism		
$[Fe(mnt)_2(H\text{-}CO_2)]^{2-}$	*concerted H^- abstraction – transition state – SMD*	not obtained
$[Fe(mnt)_2(H\text{-}CO_2)]^{2-}$	*H^- insertion – transition state – SMD*	-2240151.121
$[Fe(mnt)_2(OC(H)O)]^{2-}$	*intermediate – SMD*	-2240169.956
$[Fe(mnt)_2(OC(H)O)]^{2-}$	*$CHOO^-$ dissociation – transition state – SMD*	-2240149.348
Ferrocene/Ferrocenium ion		
Fc	*intermediate – vacuum*	-1035849.688
Fc	*intermediate – SMD*	-1035858.324
Fc^+	*intermediate – vacuum*	-1035689.093
Fc^+	*intermediate – SMD*	-1035745.304
Substrates/proton sources		
CO	*intermediate – SMD*	-71114.89565
CO_2	*intermediate – SMD*	-118351.1968
$CO_2^{\bullet -}$	*intermediate – SMD*	-118382.1182
OH^-	*intermediate – SMD*	-47595.65098
H_2O	*intermediate – SMD*	-47956.2928

H_2	*intermediate – SMD*	-737.1926475
$CHOO^-$	*intermediate – SMD*	-118778.3524
TFE-H	*intermediate – SMD*	-284110.7867
TFE^-	*intermediate – SMD*	-283789.5243
TEA^+	*intermediate – SMD*	-232968.2999

Geometry coordinates

Table S 12. Cartesian coordinates for $[Fe(mnt)_2]^{1-}$ (intermediate – SMD).

Atom	**x**	**y**	**z**
Fe	0.00000000	0.00000000	0.00000000
S	0.00000000	1.60848800	1.61035800
S	0.00000000	-1.60848800	1.61035800
S	0.00000000	1.60848800	-1.61035800
S	0.00000000	-1.60848800	-1.61035800
C	0.00000000	0.68632200	3.11321100
C	0.00000000	-0.68632200	3.11321100
C	0.00000000	0.68632200	-3.11321100
C	0.00000000	-0.68632200	-3.11321100
C	0.00000000	1.43824500	4.32609600
C	0.00000000	-1.43824500	4.32609600
C	0.00000000	1.43824500	-4.32609600
C	0.00000000	-1.43824500	-4.32609600
N	0.00000000	2.07588400	5.30232400
N	0.00000000	-2.07588400	5.30232400
N	0.00000000	2.07588400	-5.30232400
N	0.00000000	-2.07588400	-5.30232400

Table S 13. Cartesian coordinates for $[Fe(mnt)_2]^{2-}$ (intermediate – SMD).

Atom	**x**	**y**	**z**
Fe	0.00000000	0.00000000	0.00000000
S	0.00000000	1.61624800	1.62193700
S	0.00000000	-1.61624800	1.62193700
S	0.00000000	1.61624800	-1.62193700
S	0.00000000	-1.61624800	-1.62193700
C	0.00000000	0.68856400	3.12379800
C	0.00000000	-0.68856400	3.12379800
C	0.00000000	0.68856400	-3.12379800
C	0.00000000	-0.68856400	-3.12379800
C	0.00000000	1.42719800	4.34358200
C	0.00000000	-1.42719800	4.34358200
C	0.00000000	1.42719800	-4.34358200
C	0.00000000	-1.42719800	-4.34358200
N	0.00000000	2.05853800	5.32631500
N	0.00000000	-2.05853800	5.32631500
N	0.00000000	2.05853800	-5.32631500
N	0.00000000	-2.05853800	-5.32631500

Table S 14. Cartesian coordinates for $(TEA^+)[Fe(mnt)_2]^{2-}$ (intermediate – SMD).

Atom	**x**	**y**	**z**
Fe	-0.02376000	-1.70325100	0.00452900
S	-1.59171500	-1.65549600	1.67509600
S	-1.69926800	-1.70145900	-1.55615600
S	1.64664900	-1.70557200	1.56835800
S	1.54603400	-1.73250300	-1.66224200

C	-3.12344700	-1.63098400	0.79714700
C	-3.16926600	-1.65108200	-0.57881500
C	3.11960000	-1.72371300	0.59512000
C	3.07674200	-1.73695600	-0.78107400
C	-4.31755700	-1.58821700	1.57552500
C	-4.41268600	-1.63355800	-1.27678600
C	4.36132700	-1.72215000	1.29638200
C	4.27311400	-1.75353400	-1.55700400
N	-5.27798000	-1.55131500	2.23916700
N	-5.41559000	-1.61895200	-1.87518600
N	5.36226800	-1.71762900	1.89819400
N	5.23558500	-1.76690600	-2.21856300
N	-0.27221800	3.58153800	-0.10596300
C	0.89957300	2.76597400	-0.65987000
H	0.74659200	1.74739800	-0.27623600
H	0.74877800	2.73490500	-1.74485500
C	2.31233600	3.25588000	-0.38300000
H	2.51534700	4.22921600	-0.85277000
H	2.56292000	3.31159500	0.68338100
C	-0.08172400	5.09423600	-0.23362000
H	-1.09968900	5.50330300	-0.27857300
H	0.38854100	5.25298000	-1.21246200
C	0.68718000	5.82497700	0.85800800
H	1.72090300	5.48055200	0.98177600
H	0.17507300	5.78404700	1.82954200
C	-1.50942400	3.20348100	-0.94268600
H	-2.36960700	3.57037200	-0.36725200
H	-1.52990300	2.10620800	-0.93479800
C	-1.56834000	3.73093100	-2.36730300
H	-1.66878700	4.82406200	-2.41625900
H	-0.70908200	3.42438800	-2.98168100
C	-0.63910000	3.15581800	1.32082800
H	-1.34632200	3.91824000	1.67579700
H	-1.19267200	2.21498500	1.19939700
C	0.48327700	2.93257200	2.32268300
H	1.14061000	2.09990800	2.03565400
H	1.09248500	3.82324300	2.51562300
H	-2.46942400	3.29517100	-2.82783100
H	2.99124200	2.52228600	-0.84758100
H	0.72268600	6.88366200	0.55329800
H	-0.00319500	2.64928100	3.27043300

Table S 15. Cartesian coordinates for $(TEA^{+})[Fe(mnt)_2]^{3-}$ (intermediate – SMD).

Atom	**x**	**y**	**z**
Fe	-1.14722500	1.09009000	0.16897100
S	0.36738400	1.24696500	1.82212100
S	0.25905500	2.10263800	-1.26505900
S	-2.53425200	-0.01094200	1.58596300
S	-2.66805800	0.87917100	-1.50161700
C	1.80064200	1.94341500	1.03589000
C	1.75761000	2.30462700	-0.33251300
C	-3.94545600	-0.38030200	0.59271400
C	-4.00340800	0.00541100	-0.74698900
C	2.97337800	2.04637400	1.79615300
C	2.90145500	2.74157700	-1.01390500
C	-5.01081700	-1.08383600	1.20109200
C	-5.12948600	-0.29355800	-1.54856400
N	3.95890800	2.10861700	2.44121100

N	3.86877800	3.08406000	-1.59607300
N	-5.88345400	-1.66729200	1.72434600
N	-6.05441100	-0.53168100	-2.22935800
N	2.69626600	-1.87431800	-0.21569500
C	1.45910400	-1.32444900	-0.91952900
H	1.05108700	-0.56545800	-0.24101000
H	1.82787600	-0.79895400	-1.80549900
C	0.38437500	-2.30711400	-1.34641800
H	0.73896700	-3.01375900	-2.11035500
H	-0.05589400	-2.86701300	-0.51313100
C	3.22131300	-3.16835500	-0.81952600
H	4.29194400	-3.18285600	-0.57705400
H	3.12354200	-3.05124300	-1.90581300
C	2.59909600	-4.47599500	-0.35844100
H	1.52417300	-4.54853000	-0.55972900
H	2.77903100	-4.66789300	0.70826900
C	3.79556700	-0.81560500	-0.36940100
H	4.57341300	-1.09562900	0.35288900
H	3.33045900	0.11852500	-0.03709300
C	4.39265000	-0.65261200	-1.75526600
H	4.97588600	-1.52610200	-2.07810800
H	3.64056600	-0.42230700	-2.52325900
C	2.47997700	-1.97690400	1.29374300
H	3.32992400	-2.56263600	1.66826800
H	2.58467700	-0.94888100	1.66175300
C	1.15515900	-2.52883000	1.78975200
H	0.31493700	-1.87542800	1.51949800
H	0.94845600	-3.55326000	1.45989200
H	5.07437500	0.21059400	-1.70683600
H	-0.41862200	-1.70307100	-1.79781200
H	3.10089400	-5.27700500	-0.92448500
H	1.21655500	-2.53550200	2.89006000

Table S 16. Cartesian coordinates for $[FeH(mnt)_2]^{2-}$ (Fe-H bond scan (2.68 Å length) – SMD).

Atom	**x**	**y**	**z**
Fe	0.00000800	-0.00003000	0.01835600
S	-1.63087100	1.61836700	-0.02653500
S	-1.63084000	-1.61833600	-0.02530900
S	1.63079900	1.61831100	-0.02537600
S	1.63087900	-1.61836800	-0.02637100
C	-3.12429300	0.69156000	-0.02232600
C	-3.12432700	-0.69156800	-0.02182000
C	3.12431700	0.69158500	-0.02189000
C	3.12429500	-0.69154500	-0.02223100
C	-4.34476900	1.42754600	-0.02021800
C	-4.34483800	-1.42744200	-0.01925000
C	4.34480700	1.42748900	-0.01949400
C	4.34476500	-1.42754500	-0.02016800
N	-5.33101600	2.05420200	-0.01870400
N	-5.33096700	-2.05428700	-0.01781600
N	5.33099000	2.05424600	-0.01754900
N	5.33108800	-2.05408000	-0.01897300
H	-0.00010300	0.00013100	2.69587100

Table S 17. Cartesian coordinates for $[FeH(mnt)_2]^{2-}$ (intermediate – SMD).

Atom	**x**	**y**	**z**
Fe	0.00000000	0.00000000	0.08940100

S	-1.60108800	1.60716200	0.01169500
S	-1.59394100	-1.60005300	-0.10645300
S	1.59394200	1.60005200	-0.10645200
S	1.60108600	-1.60715900	0.01169300
C	-3.09793700	0.69091400	0.00433200
C	-3.09590900	-0.69156600	-0.04622400
C	3.09591100	0.69156600	-0.04623200
C	3.09793700	-0.69091300	0.00432700
C	-4.31709200	1.42820800	0.04320100
C	-4.31215500	-1.43373600	-0.06492700
C	4.31215700	1.43373400	-0.06494000
C	4.31709100	-1.42820900	0.04319300
N	-5.30229700	2.05527700	0.07467900
N	-5.29508100	-2.06511000	-0.08207800
N	5.29508200	2.06511000	-0.08208300
N	5.30229500	-2.05528100	0.07465100
H	0.00000000	-0.00000200	1.57529300

Table S 18. Cartesian coordinates for $[Fe(mnt)(mnt\text{-}H)]^{2-}$ (intermediate – SMD).

Atom	**x**	**y**	**z**
Fe	-0.02098100	0.01408400	0.00631100
S	1.66589800	-1.62024300	0.03461300
S	1.56817400	1.60730400	-0.03721400
S	-1.62975200	-1.61236600	-0.00194900
S	-1.63412700	1.61366600	-0.01152400
C	3.18395100	-0.69349900	-0.03037800
C	3.11892300	0.73905400	0.02801900
C	-3.13483700	-0.69124900	-0.00130700
C	-3.13466700	0.68756400	-0.01407100
C	4.36830300	-1.43066200	-0.10984200
C	4.28117400	1.51810800	0.06441100
C	-4.35151100	-1.43255700	0.00574100
C	-4.35295500	1.42770900	-0.02939900
N	5.36782600	-2.05223200	-0.17698500
N	5.25438600	2.18465000	0.09538200
N	-5.33373400	-2.06517400	0.01286600
N	-5.33545700	2.05935800	-0.04267200
H	1.48097200	-1.81299900	1.39390000

Table S 19. Cartesian coordinates for $[Fe(mnt)(mnt\text{-}H)]^{2-}$ (H rearrangement – transition state – SMD).

Atom	**x**	**y**	**z**
Fe	0.00326600	-0.00037700	-0.05983700
S	1.62029500	-1.59482700	0.09081100
S	1.57482900	1.57975600	-0.32212900
S	-1.62470900	-1.58592700	-0.32151300
S	-1.59069500	1.58929000	0.26016600
C	3.12822900	-0.69635600	0.07583900
C	3.10308000	0.70861400	-0.13710200
C	-3.11380400	-0.67378300	-0.09515700
C	-3.10060600	0.68953700	0.13436700
C	4.30246900	-1.42595000	0.34317000
C	4.29201600	1.46056100	-0.19490400
C	-4.33750400	-1.39913900	-0.16378600
C	-4.31023600	1.42533700	0.28806900
N	5.28217700	-2.04008200	0.54743600
N	5.27543600	2.10274500	-0.24284200
N	-5.32806700	-2.01665300	-0.22254700

N	-5.28723300	2.05385700	0.41655100
H	0.86151100	-1.03486600	1.24523700

Table S 20. Cartesian coordinates for [FeH(mnt)(mnt-H)]$^{1-}$ (sulfur (cis) protonation – transition state – SMD).

Atom	**x**	**y**	**z**
Fe	0.01040500	-0.01571300	0.03292700
S	-1.54750300	1.54332600	-0.09122100
S	-1.53279600	-1.58929000	-0.01259800
S	1.55548500	1.55574500	-0.05536100
S	1.56762900	-1.57678400	-0.01263200
C	-3.04293400	0.66598200	-0.05515700
C	-3.03507000	-0.72408600	-0.01864300
C	3.05650200	0.69111300	-0.02762300
C	3.06185600	-0.70029800	-0.00990600
C	-4.25768100	1.41447600	-0.06939600
C	-4.24384200	-1.48311300	0.00230900
C	4.26605800	1.44904000	-0.03086200
C	4.27701100	-1.44921500	0.00229000
N	-5.23984500	2.04242200	-0.07953800
N	-5.22098300	-2.11860600	0.01899200
N	5.24403200	2.08348600	-0.03416500
N	5.25939100	-2.07676200	0.01063600
H	-0.00118400	0.01138100	1.49763400
H	-1.74397400	2.77201800	2.22570200

Table S 21. Cartesian coordinates for [FeH(mnt)(mnt-H)]$^{1-}$ ((cis) intermediate – SMD).

Atom	**x**	**y**	**z**
Fe	-0.04282300	0.00712600	0.01819500
S	1.64326200	-1.62016600	-0.18030500
S	1.56997400	1.61558800	-0.09839900
S	-1.60554200	-1.58246400	-0.08940700
S	-1.62271900	1.60091400	-0.01267800
C	3.15637200	-0.66221000	0.01527200
C	3.06256400	0.70642600	-0.01772300
C	-3.11893200	-0.68684200	-0.01729300
C	-3.12020800	0.69187800	0.00455300
C	4.37800700	-1.37746000	0.08565600
C	4.26518300	1.49181900	0.00592100
C	-4.32508000	-1.44362900	0.01923400
C	-4.33620200	1.43719000	0.05057700
N	5.36769200	-1.99207200	0.15568100
N	5.22177300	2.15446900	0.02841900
N	-5.29968400	-2.08560200	0.04571000
N	-5.31710200	2.06786800	0.08748000
H	0.09889300	-0.11684000	1.48630100
H	1.67593500	-2.24604200	1.03504600

Table S 22. Cartesian coordinates for [FeH(mnt)(mnt-H)]$^{1-}$ (sulfur (trans) protonation – transition state – SMD).

Atom	**x**	**y**	**z**
Fe	-0.01363100	-0.02036500	-0.05091200
S	1.55510000	1.54737600	-0.01709800
S	1.53689100	-1.60581900	0.03909300
S	-1.56359700	1.56773800	-0.01536700
S	-1.58126100	-1.58333700	0.08803000
C	3.05699100	0.65273900	-0.02637800

C	3.04886700	-0.72753100	-0.00107000
C	-3.07571800	0.68891400	-0.00901900
C	-3.08332600	-0.69073800	0.04016600
C	4.27037800	1.40249300	-0.06520700
C	4.25390500	-1.49148900	-0.00845300
C	-4.28087700	1.45079800	-0.06092600
C	-4.29687900	-1.44118900	0.05092800
N	5.24977200	2.03414700	-0.09578000
N	5.22633600	-2.13457400	-0.01090800
N	-5.25352600	2.09216600	-0.10370200
N	-5.27627500	-2.07347300	0.06278000
H	-0.02844900	-0.05227000	-1.52204100
H	2.24453500	3.27459200	2.84425100

Table S 23. Cartesian coordinates for $[FeH(mnt)(mnt\text{-}H)]^{1-}$ ((trans) intermediate – SMD).

Atom	x	y	z
Fe	-0.04018200	0.00692800	0.04829500
S	1.62911300	-1.63241100	-0.01643300
S	1.57242600	1.61679900	-0.12476900
S	-1.60640100	-1.58849100	-0.01226000
S	-1.61725400	1.59977800	-0.00154600
C	3.15042300	-0.66406400	-0.01662000
C	3.05848200	0.70610800	-0.03002200
C	-3.11658400	-0.68691100	-0.01166600
C	-3.11638900	0.69195100	-0.00731800
C	4.37243100	-1.37975100	0.03924500
C	4.26235300	1.48915300	0.02018100
C	-4.32597800	-1.44002400	-0.00133300
C	-4.33135200	1.43982600	0.00484200
N	5.36481500	-1.99336600	0.07390900
N	5.22094400	2.14809600	0.05926100
N	-5.30315300	-2.07841500	0.00229300
N	-5.31160700	2.07253400	0.01532600
H	0.18650600	-0.06061800	1.51274300
H	1.69476700	-2.02995900	-1.32767400

Table S 24. Cartesian coordinates for $[FeH(mnt)(mnt\text{-}H)]^{1-}$ (H-S-Fe-H torsion scan cis-trans barrier – SMD).

Atom	x	y	z
Fe	0.08743400	-0.05800400	-0.48945400
S	-1.81040300	-1.38721000	-0.71364200
S	-1.43886600	1.51401400	0.02311800
S	1.40614300	-1.42841700	0.55525500
S	1.74973900	1.47976500	-0.60373100
C	-3.23580400	-0.60549000	-0.04034500
C	-3.00666000	0.72827200	0.27287100
C	3.01946500	-0.70352400	0.36861100
C	3.13711500	0.59404900	-0.06871500
C	-4.47395300	-1.26840300	0.01832200
C	-4.02537000	1.54776300	0.80771900
C	4.16671800	-1.45346000	0.68063200
C	4.38683200	1.27154400	-0.05594100
N	-5.50468300	-1.81985700	0.03848200
N	-4.83502700	2.25811500	1.27994500
N	5.10301300	-2.10770300	0.94777100
N	5.40598800	1.86953700	-0.05506000
H	0.53173500	-0.82806500	-1.72469000
H	-2.30592900	-2.57941200	-1.10239900

Table S 25. Cartesian coordinates for $[FeH(mnt)(mnt-H)]^{1-}$ (H_2 dissociation – transition state – SMD).

Atom	x	y	z
Fe	-0.03766100	-0.00128400	-0.00192300
S	1.61615000	-1.59359000	-0.21837000
S	1.56538500	1.59415000	-0.09551000
S	-1.59761500	-1.57214200	-0.08924100
S	-1.60368300	1.58150100	-0.03331600
C	3.14256600	-0.66576000	0.00973900
C	3.04933700	0.68715000	-0.01805200
C	-3.10283100	-0.67973900	-0.00735100
C	-3.10039000	0.68614000	0.00461400
C	4.33525300	-1.39593000	0.09473900
C	4.24489600	1.47061500	0.01971600
C	-4.29797100	-1.43440000	0.04456100
C	-4.29934600	1.43862300	0.05241500
N	5.30350200	-2.02091500	0.17741600
N	5.19113000	2.12690000	0.05053400
N	-5.26036600	-2.07324900	0.08401300
N	-5.26377400	2.07331100	0.08913600
H	0.51704900	-0.42324400	1.57735700
H	1.15580600	-1.08459000	1.44566200

Table S 26. Cartesian coordinates for $(TEA^+)[Fe(mnt)_2(\eta^1-CO_2)]^{3-}$ (Fe-C bond scan (3.16 Å length) – SMD).

Atom	x	y	z
Fe	1.32946700	0.49815900	0.00447400
S	0.04056700	1.46359800	-1.61620500
S	0.17673500	1.61467900	1.57546000
S	2.43468500	-0.67381800	-1.56968100
S	2.56218800	-0.54229000	1.62563400
C	-1.07084300	2.52821900	-0.72609000
C	-1.00264200	2.59674700	0.69486100
C	3.60719200	-1.65992900	-0.68688900
C	3.66902100	-1.60004700	0.73124200
C	-2.03441600	3.23754400	-1.45643900
C	-1.86481800	3.41842100	1.44020500
C	4.45542000	-2.49878300	-1.43082900
C	4.58881700	-2.36967200	1.46237600
N	-2.85051900	3.81127800	-2.09241800
N	-2.58187400	4.10427300	2.08093200
N	5.15941500	-3.19620000	-2.07219000
N	5.35477800	-3.00761000	2.09615400
C	3.51567000	2.77203400	-0.13647300
O	2.77820400	3.68749200	-0.15054500
O	4.45584000	2.06745300	-0.13775200
N	-3.60129100	-1.90720600	-0.13305100
C	-3.44093100	-1.47122200	1.32566400
H	-2.79906400	-0.58087600	1.28931200
H	-2.86675100	-2.26989300	1.80860000
C	-4.69746200	-1.21957800	2.14500600
H	-5.29792600	-2.13113600	2.27694300
H	-5.33667500	-0.42215000	1.74691600
C	-4.71057200	-2.93630600	-0.34561800
H	-4.41120700	-3.49344700	-1.24331600
H	-4.64856900	-3.62094700	0.50979800
C	-6.13606900	-2.43837500	-0.53801500
H	-6.52623000	-1.86666400	0.31255700

H	-6.25138700	-1.84431900	-1.45554400
C	-2.27426600	-2.57358500	-0.54423000
H	-2.31415600	-2.64301100	-1.63954100
H	-1.49298400	-1.84917100	-0.27394800
C	-1.97198400	-3.93678700	0.05681500
H	-2.65984900	-4.72276800	-0.28539600
H	-1.95240000	-3.93297400	1.15651400
C	-3.72557000	-0.70594300	-1.08058000
H	-4.03358400	-1.13293800	-2.04497700
H	-2.70016100	-0.32765600	-1.19274300
C	-4.62796500	0.45029600	-0.67491400
H	-4.28010900	0.94723300	0.24094800
H	-5.68303100	0.17426400	-0.55734900
H	-0.95973900	-4.21033800	-0.28246300
H	-4.35818000	-0.90577400	3.14569100
H	-6.76434300	-3.33700400	-0.65143000
H	-4.56399000	1.19324200	-1.48637500

Table S 27. Cartesian coordinates for $(TEA^+)[Fe(mnt)_2(\eta^1\text{-}CO_2)]^{3-}$ (intermediate – SMD).

Atom	x	y	z
Fe	-0.41549100	1.38892500	0.01119900
S	1.24321600	1.23056800	1.60919900
S	1.19145700	1.63193500	-1.61110300
S	-1.92503000	0.78062500	1.62709500
S	-1.83012600	0.56515700	-1.60298900
C	2.72290900	1.36906200	0.67516000
C	2.69905700	1.53410900	-0.71749400
C	-3.28157900	0.15417600	0.70604500
C	-3.23971700	0.06231600	-0.69186900
C	3.95390100	1.25131000	1.36225300
C	3.90866100	1.57810300	-1.45140700
C	-4.40883300	-0.31554500	1.42087800
C	-4.32263200	-0.50751500	-1.40196600
N	4.95865800	1.13723300	1.95953100
N	4.89659800	1.60298800	-2.08528100
N	-5.32721600	-0.70898200	2.03787900
N	-5.20421100	-0.98329300	-2.01481400
C	-0.92668200	3.27785100	0.06101500
O	-0.02699900	4.10286500	0.38284000
O	-2.12632600	3.54054000	-0.22964700
N	0.65345900	-2.87030400	0.19857600
C	0.95606900	-1.83403300	-0.87829100
H	1.07735100	-0.88637300	-0.34040300
H	0.03804700	-1.73189800	-1.46462900
C	2.11971100	-2.09450200	-1.81562700
H	1.96088400	-2.98107600	-2.44689800
H	3.08782400	-2.18461800	-1.30866300
C	0.80382300	-4.30864600	-0.26862000
H	0.13527800	-4.89105100	0.37874800
H	0.39467200	-4.33474600	-1.28569100
C	2.18691700	-4.93660300	-0.22868200
H	2.92956500	-4.40843000	-0.83746000
H	2.56754100	-5.03689400	0.79688300
C	-0.81613900	-2.66342100	0.59536500
H	-0.93816700	-3.20255900	1.54438600
H	-0.92071900	-1.58620900	0.78625000
C	-1.85283500	-3.12672200	-0.41201700
H	-1.83755000	-4.21230800	-0.58122100

H	-1.76738000	-2.60465400	-1.37472200
C	1.43749800	-2.57576100	1.47412100
H	1.27973800	-3.44690300	2.12448100
H	0.92915700	-1.70843500	1.91484400
C	2.91078100	-2.23167100	1.34123700
H	3.06419100	-1.32512200	0.74402700
H	3.52591000	-3.04357500	0.93744900
H	-2.83682800	-2.86822900	0.00685800
H	2.17007600	-1.21540700	-2.47719700
H	2.07966400	-5.95370300	-0.63878400
H	3.27136700	-2.00820200	2.35792600

Table S 28. Cartesian coordinates for $[Fe(mnt)_2(C(O)OH)]^{2-}$ (protonation – transition state – SMD).

Atom	**x**	**y**	**z**
Fe	0.00914900	-0.00116800	-0.00409400
S	1.63398800	1.56710200	-0.12316000
S	1.52450800	-1.54170300	0.69165200
S	-1.50667100	1.50598200	0.79995300
S	-1.61329000	-1.57869800	-0.14636100
C	3.09856400	0.65977100	0.23750300
C	3.05031100	-0.67455600	0.58355000
C	-3.02719100	0.64261700	0.63847400
C	-3.07640900	-0.67697600	0.23676100
C	4.33478500	1.36785000	0.17555600
C	4.23859600	-1.40609300	0.88077900
C	-4.21902800	1.35922400	0.95918100
C	-4.31422200	-1.37749500	0.13984100
N	5.33342200	1.96977900	0.11739700
N	5.19479800	-2.02811000	1.12886100
N	-5.17806900	1.96813800	1.22809500
N	-5.31442200	-1.97366600	0.05175100
C	-0.01354200	0.01569400	-2.28269200
O	-0.80785700	0.89978100	-2.56158800
O	0.73197900	-0.80661400	-2.72961500
H	-0.92869700	1.02890600	-4.21373600

Table S 29. Cartesian coordinates for $[Fe(mnt)_2(C(O)OH)]^{2-}$ (intermediate – SMD).

Atom	**x**	**y**	**z**
Fe	0.02024300	0.01368100	-0.01678600
S	1.63230900	-1.59571400	-0.18191200
S	1.57079300	1.60596900	-0.47744100
S	-1.56792200	-1.52881800	-0.61087500
S	-1.59143800	1.63705900	0.03656700
C	3.10838500	-0.65972700	-0.31111000
C	3.08586500	0.71719000	-0.43939400
C	-3.07567100	-0.61083500	-0.51301800
C	-3.07214900	0.74383100	-0.24499500
C	4.33907700	-1.38055200	-0.29807100
C	4.29049300	1.46759900	-0.56900500
C	-4.28743800	-1.32266400	-0.74092200
C	-4.29387300	1.48154100	-0.18820500
N	5.33348700	-1.99278900	-0.28619300
N	5.26234900	2.10667900	-0.67761500
N	-5.26645000	-1.93089400	-0.93183800
N	-5.27825400	2.10678500	-0.13926300
C	0.02572400	-0.15324600	1.92688000
O	-0.88922300	-1.01340100	2.45499100

O	0.76940900	0.44543200	2.66841200
H	-1.34808900	-1.43518400	1.69921300

Table S 30. Cartesian coordinates for $[Fe(mnt)_2(C(O)OH)]^{2-}$ (OH^- dissociation – transition state – SMD).

Atom	x	y	z
Fe	0.03810900	0.04080100	0.18904100
S	1.69719700	-1.54910300	-0.22411200
S	1.62456600	1.71137000	-0.17986200
S	-1.55476800	-1.42180600	-0.65243900
S	-1.61014800	1.67539600	0.30873700
C	3.14528600	-0.57667100	-0.41898400
C	3.12108400	0.80554500	-0.39564400
C	-3.02794600	-0.48204500	-0.65873800
C	-3.03754700	0.84224400	-0.23044100
C	4.36855500	-1.28167200	-0.62393600
C	4.31615900	1.55990900	-0.57808200
C	-4.21796200	-1.12976200	-1.09508800
C	-4.25620000	1.58950100	-0.23135900
N	5.35646900	-1.88294900	-0.78950700
N	5.27980700	2.20541700	-0.72315900
N	-5.18041800	-1.68231300	-1.46150700
N	-5.23874700	2.21970700	-0.22447700
C	0.20246900	-0.24514100	2.19961700
O	-1.47180300	-1.85671500	1.70009600
O	0.48962900	-0.34627500	3.30456200
H	-0.84615400	-2.60109400	1.59698600

Table S 31. Cartesian coordinates for $[Fe(mnt)_2(CO)]^{1-}$ (intermediate – SMD).

Atom	x	y	z
Fe	-0.00002000	0.00000200	0.01283700
S	-1.58608200	1.59563500	-0.15207800
S	-1.57359000	-1.59010000	-0.25425900
S	1.57354300	1.59012400	-0.25417400
S	1.58605300	-1.59561900	-0.15218300
C	-3.07299400	0.69051600	-0.21990000
C	-3.06761900	-0.69347600	-0.26797000
C	3.06757700	0.69351000	-0.26790200
C	3.07296000	-0.69048700	-0.21989900
C	-4.29188800	1.43394500	-0.22674200
C	-4.28081500	-1.44309200	-0.33576900
C	4.28077100	1.44314100	-0.33558200
C	4.29185900	-1.43390800	-0.22666200
N	-5.27532000	2.06011200	-0.23320400
N	-5.25953400	-2.07397800	-0.39406100
N	5.25949300	2.07402700	-0.39380400
N	5.27530100	-2.06006100	-0.23310000
C	-0.00003000	-0.00004000	1.77695300
O	0.00040300	-0.00025600	2.92367100

Table S 32. Cartesian coordinates for $[Fe(mnt)_2(CO)]^{1-}$ (CO dissociation – transition state – SMD).

Atom	x	y	z
Fe	-0.00234100	-0.12048300	-0.25526600
S	-1.60784700	1.46681200	-0.52562800
S	-1.60603000	-1.70700700	-0.00094800
S	1.60503600	1.44458000	-0.62856800

S	1.59671400	-1.69236300	0.09472700
C	-3.08471900	0.56943500	-0.35599900
C	-3.08354800	-0.80532400	-0.12826200
C	3.07988100	0.55477000	-0.40845300
C	3.07580500	-0.80176100	-0.08870300
C	-4.31061600	1.29342900	-0.46667400
C	-4.30742900	-1.52914400	0.00564100
C	4.30622600	1.26689900	-0.57732800
C	4.29856900	-1.51558900	0.09933300
N	-5.30112900	1.90147700	-0.56131300
N	-5.29489000	-2.13864600	0.12040600
N	5.29593600	1.86659500	-0.72102200
N	5.28560500	-2.11548500	0.25937100
C	0.01828900	0.89237300	2.61276500
O	0.05019000	1.84901400	3.22094700

Table S 33. Cartesian coordinates for $[Fe(mnt)_2(H\text{-}CO_2)]^{2-}$ (H^- insertion – transition state – SMD).

Atom	**x**	**y**	**z**
Fe	0.00590000	-0.14938100	0.21350900
S	-1.40345800	1.53701600	0.71904400
S	-1.72428100	-1.26965200	-0.80509900
S	1.72586600	0.86894300	1.35814600
S	1.27104800	-0.31068300	-1.63136700
C	-2.95183300	1.02734100	0.05103100
C	-3.08042200	-0.18171500	-0.60243100
C	3.04529600	0.70340500	0.21672600
C	2.86406900	0.18590100	-1.04978100
C	-4.05546900	1.91755100	0.19626500
C	-4.33252400	-0.59333500	-1.15365200
C	4.33325300	1.14226500	0.64983700
C	3.95023800	0.04872000	-1.96071100
N	-4.94196800	2.66545900	0.33072000
N	-5.34410400	-0.95292600	-1.61059400
N	5.37488800	1.50880600	1.02789500
N	4.82259900	-0.07395500	-2.72758600
C	0.21822200	-2.34604100	1.70002000
H	0.59370000	-1.62086200	0.65948700
O	-0.39896900	-1.68358600	2.51754300
O	0.65162000	-3.46159700	1.49631000

Table S 34. Cartesian coordinates for $[Fe(mnt)_2(OC(H)O)]^{2-}$ (intermediate – SMD).

Atom	**x**	**y**	**z**
Fe	-0.00003000	0.09031400	-0.03752600
S	1.58847200	-1.36278100	-0.82529700
S	1.60368700	1.73843700	0.06739000
S	-1.58861500	-1.36297800	-0.82480200
S	-1.60384600	1.73830800	0.06782600
C	3.08609400	-0.48085700	-0.57532400
C	3.09119000	0.84721700	-0.19869600
C	-3.08622500	-0.48096200	-0.57507800
C	-3.09133100	0.84711800	-0.19847400
C	4.30159400	-1.18850100	-0.81219400
C	4.31490500	1.56455500	-0.04356000
C	-4.30172500	-1.18856100	-0.81208800
C	-4.31504100	1.56450000	-0.04350600
N	5.28196500	-1.79048000	-1.01130900
N	5.30212700	2.17377000	0.08726700

N	-5.28210000	-1.79049800	-1.01130800
N	-5.30225600	2.17376000	0.08716400
O	0.00041300	-0.34244800	1.79382800
C	0.00037600	-1.55411200	2.29872300
O	0.00062800	-1.77337300	3.50068800
H	0.00009900	-2.38562800	1.55617600

Table S 35. Cartesian coordinates for $[Fe(mnt)_2(OC(H)O)]^{2-}$ ($CHOO^-$ dissociation – transition state – SMD).

Atom	**x**	**y**	**z**
Fe	-0.09837800	-0.35810300	0.14158100
S	-1.69542500	-1.04933100	-1.30942500
S	-1.69774200	0.35096100	1.58118800
S	1.48325100	-1.26306400	-1.21181800
S	1.49765500	0.15092600	1.66710000
C	-3.18790900	-0.65289300	-0.48793300
C	-3.18700900	-0.04912100	0.75652700
C	2.96993200	-0.94785300	-0.35736300
C	2.97380500	-0.33872200	0.88958000
C	-4.40770800	-0.99171500	-1.14801200
C	-4.40800900	0.27521100	1.42257900
C	4.18558000	-1.36361400	-0.98015400
C	4.19615500	-0.10485600	1.59178300
N	-5.39089600	-1.27686200	-1.70677800
N	-5.39158900	0.54925600	1.98609100
N	5.16552500	-1.71096300	-1.50839500
N	5.18131800	0.09418200	2.18268300
O	1.31711800	4.11432400	-2.16895800
C	0.60412100	3.69517200	-1.23722700
O	0.40004200	2.51767400	-0.83667600
H	0.03248900	4.50388000	-0.64265700

References

(1) Weiher, J. F.; Melby, L. R.; Benson, R. E. 1,2-Dicyanoethylene-1,2-Dithiolate Chelates. *J. Am. Chem. Soc.* **1964**, *86* (20), 4329–4333. https://doi.org/10.1021/ja01074a019.

(2) Hogue, R.; Armstrong, C.; Toghill, K. Dithiolene Complexes of First Row Transition Metals for Symmetric Non-Aqueous Redox Flow Batteries. *ChemSusChem* **2019**, *12*, 4506–4515. https://doi.org/10.1002/cssc.201901702.

(3) Dolomanov, O. V.; Bourhis, L. J.; Gildea, R. J.; Howard, J. A. K.; Puschmann, H. OLEX2: A Complete Structure Solution, Refinement and Analysis Program. *J. Appl. Crystallogr.* **2009**, *42* (2), 339–341. https://doi.org/10.1107/S0021889808042726.

(4) Sheldrick, G. M. SHELXT - Integrated Space-Group and Crystal-Structure Determination. *Acta Crystallogr. Sect. A Found. Crystallogr.* **2015**, *71* (1), 3–8. https://doi.org/10.1107/S2053273314026370.

(5) Sheldrick, G. M. Crystal Structure Refinement with SHELXL. *Acta Crystallogr. Sect. C Struct. Chem.* **2015**, *71* (Md), 3–8. https://doi.org/10.1107/S2053229614024218.

(6) Hurvois, J. P.; Moinet, C. Reactivity of Ferrocenium Cations with Molecular Oxygen in Polar Organic Solvents: Decomposition, Redox Reactions and Stabilization. *J. Organomet. Chem.* **2005**, *690* (7), 1829–1839. https://doi.org/10.1016/j.jorganchem.2005.02.009.

(7) Frisch, M. J.; Trucks, G. W.; Schlegel, H. B.; Scuseria, G. E.; Robb, M. A.; Cheeseman, J. R.; Scalmani, G.; Barone, V.; Petersson, G. A.; Nakatsuji, H.; Li, X.; Caricato, M.; Marenich, A.; Bloino, J.; Janesko, B. G.; Gomperts, R.; Mennucci, B.; Hratchian, H. P.; Ortiz, J. V.; Izmaylov, A. F.; Sonnenberg, J. L.; Williams-Young, D.; Ding, F.; Lipparini, F.; Egidi, F.; Goings, J.; Peng, B.; Petrone, A.; Henderson, T.; Ranasinghe, D.; Zakrzewski, V. G.; Gao, J.; Rega, N.; Zheng, G.; Liang, W.; Hada, M.; Ehara, M.; Toyota, K.; Fukuda, R.; Hasegawa, J.; Ishida, M.; Nakajima, T.; Honda, Y.; Kitao, O.; Nakai, H.; Vreven, T.; Throssell, K.; Montgomery, J. A. J.; Peralta, J. E.; Ogliaro, F.; Bearpark, M.; Heyd, J. J.; Brothers, E.; Kudin, K. N.; Staroverov, V. N.; Keith, T.; Kobayashi, R.; Normand, J.; Raghavachari, K.; Rendell, A.; Burant, J. C.; Iyengar, S. S.; Tomasi, J.; Cossi, M.; Millam, J. M.; Klene, M.; Adamo, C.; Cammi, R.; Ochterski, J. W.; Martin, R. L.; Morokuma, K.; Farkas, O.; Foresman, J. B.; Fox, D. J. Gaussian 09, Revision A.02. *Gaussian, Inc., Wallingford CT, 2016*.

(8) Lee, C.; Yang, W.; Parr, R. G. Development of the Colle-Salvetti Correlation-Energy Formula into a Functional of the Energy Density. *Phys. Rev. B* **1988**, *37* (2), 785–789.

(9) Becke, A. D. A New Mixing of Hartree-Fock and Local Density-Functional Theories. *J. Chem. Phys.* **1993**, *98* (2), 1372–1377. https://doi.org/10.1063/1.464304.

(10) Becke, A. D. Density-Functional Exchange-Energy Approximation with Correct Asymptotic Behaviour. *Phys. Rev. A* **1988**, *38* (6), 3098–3100.

(11) Krishnan, R.; Binkley, J. S.; Seeger, R.; Pople, J. A. Self-Consistent Molecular Orbital Methods. XX. A Basis Set for Correlated Wave Functions. *J. Chem. Phys.* **1980**, *72* (1), 650–654. https://doi.org/10.1063/1.438955.

(12) Miehlich, B.; Savin, A.; Stoll, H.; Preuss, H. Results Obtained with the Correlation Energy Density Functionals of Becke and Lee, Yang and Parr. *Chem. Phys. Lett.* **1989**, *157* (3), 200–206.

(13) Wilson, A. K.; Van Mourik, T.; Dunning, T. H. Gaussian Basis Sets for Use in Correlated Molecular Calculations. VI. Sextuple Zeta Correlation Consistent Basis Sets for Boron through Neon. *J. Mol. Struct. THEOCHEM* **1996**, *388* (1–3), 339–349. https://doi.org/10.1016/s0166-

1280(96)80048-0.

(14) Dunning Jr, T. H. Gaussian Basis Sets for Use in Correlated Molecular Calculations. I. The Atoms Boron through Neon and Hydrogen. *J. Chem. Phys.* **1989**, *90* (2), 1007–1023.

(15) Miertuŝ, S.; Tomasi, J. Approximate Evaluations of the Electrostatic Free Energy and Internal Energy Changes in Solution Processes. *Chem. Phys.* **1982**, *65* (2), 239–245. https://doi.org/10.1016/0301-0104(82)85072-6.

(16) Miertuš, S.; Scrocco, E.; Tomasi, J. Electrostatic Interaction of a Solute with a Continuum. A Direct Utilizaion of AB Initio Molecular Potentials for the Prevision of Solvent Effects. *Chem. Phys.* **1981**, *55* (1), 117–129. https://doi.org/10.1016/0301-0104(81)85090-2.

(17) Waters, T.; Wang, X. Bin; Woo, H. K.; Wang, L. S. Photoelectron Spectroscopy of the Bis(Dithiolene) Anions [M(Mnt) 2]n- (M = Fe - Zn; n = 1, 2): Changes in Electronic Structure with Variation of Metal Center and with Oxidation. *Inorg. Chem.* **2006**, *45* (15), 5841–5851. https://doi.org/10.1021/ic060255z.

(18) Davis, A. P.; Fry, A. J. Experimental and Computed Absolute Redox Potentials of Polycyclic Aromatic Hydrocarbons Are Highly Linearly Correlated over a Wide Range of Structures and Potentials. *J. Phys. Chem. A* **2010**, *114* (46), 12299–12304. https://doi.org/10.1021/jp106088n.
